# An inherited predisposition allele promotes gastric cancer via enhancing deubiquitination-mediated activation of epithelial-to-mesenchymal transition signaling

**DOI:** 10.1172/JCI179617

**Published:** 2025-02-25

**Authors:** Bolin Tao, Zhenning Wang, Xuanyi Wang, Aixia Song, Jiaxian Liu, Jianan Wang, Qin Zhang, Zhaolin Chen, Zixian Wang, Wenjie Xu, Menghong Sun, Yanong Wang, Ping Zhang, Tao Xu, Gong-Hong Wei, Fei Xavier Chen, Mengyun Wang

**Affiliations:** 1 Cancer Institute, Department of Radiation Oncology, Fudan University Shanghai Cancer Center; Department of Oncology, Shanghai Medical College, Institutes of Biomedical Sciences, Shanghai Key Laboratory of Medical Epigenetic, Fudan University, Shanghai, China.; 2 Department of Oncology, Sichuan Provincial People’s Hospital, University of Electronic Science and Technology of China, Chengdu, China.; 3 Guangdong Provincial Key Laboratory of Medical Molecular Diagnostics, the First Dongguan Affiliated Hospital, Guangdong Medical University, Dongguan, China.; 4 Disease Networks Research Unit, Faculty of Biochemistry and Molecular Medicine, Biocenter Oulu, University of Oulu, Oulu, Finland.; 5 Department of Pharmacy, The First Affiliated Hospital of University of Science and Technology of China, Division of Life Sciences and Medicine, University of Science and Technology of China, Anhui Provincial Hospital, Hefei, China.; 6 MOE Key Laboratory of Metabolism and Molecular Medicine, Department of Biochemistry and Molecular Biology, School of Basic Medical Sciences, and Fudan University Shanghai Cancer Center, Shanghai Medical College of Fudan University, Shanghai, China.; 7 Department of Pathology, Fudan University Shanghai Cancer Center; Department of Oncology, Shanghai Medical College, Fudan University, and; 8 Department of Gastric Surgery, Fudan University Shanghai Cancer Center, Fudan University, Shanghai, China.; 9 Inflammation and Immune Mediated Diseases Laboratory of Anhui Province, Anhui Institute of Innovative Drugs, School of Pharmacy, Anhui Medical University, Hefei, China.

**Keywords:** Genetics, Oncology, Gastric cancer, Genetic variation, Transcription

## Abstract

Genome-wide human genetic studies have identified inherited cis-regulatory loci variants that predispose to cancers. However, the mechanisms by which these germline variants influence cancer progression, particularly through gene expression and proteostasis control, remain unclear. By analyzing genomic data from a gastric cancer (GC) case-control study (2,117 individuals), focusing on the ubiquitin-specific protease (USP) family, we identify the SNP rs72856331 (G>A) in the promoter region of the proto-oncogene *USP47* as a putative susceptibility allele for GC. Mechanistically, the risk allele G is associated with enhanced *USP47* expression, mediated by altered recruitment of the transcription factor GLI3 and changes in the epigenetic status at promoter. CRISPR/Cas9-mediated single-nucleotide conversion into risk allele G results in increased GLI3 binding and subsequent *USP47* upregulation. The depletion of GLI3 results in a reduction of cancer-related phenotypes, similar to those observed following *USP47* knockdown. Furthermore, we identify Snai1 as a deubiquitination target of USP47, explaining USP47-dependent activation of the epithelial-mesenchymal transition pathway and tumor progression. Our findings identify an important genetic predisposition that implicates the perturbation of transcription and proteostasis programs in GC, offering insights into prevention and therapeutic strategies for genetically stratified patients.

## Abstract 2

Tao et al. have identified an inherited genetic variant that increase the risk of gastric cancer by regulating gene expression and protein stability.

## Introduction

Gastric cancer (GC) ranks as the fifth most prevalent malignancy and is the fourth leading cause of cancer-related deaths worldwide (1). Approximately half of all new cases are reported in Asia, with China accounting for a significant proportion (2). The interplay of genetic predisposition and environmental exposure is crucial for the pathogenesis and progression of GC. Predominantly, chronic *Helicobacter pylori* infection is identified as a prevalent etiological factor for GC (3). Established environmental risk factors for GC include alcohol consumption, diets deficient in fruit and vegetables, the intake of preserved foods, and potential gene-environment interaction (4, 5). The fact that only a minority of individuals infected with *H*. *pylori* eventually develop GC underscores the important role of genetic susceptibility in the etiology of this disease.

In recent decades, genome-wide association studies (GWAS) have discovered numerous independent SNP loci associated with the risk of GC (6–12). However, the stringent threshold considered to be significant set at a *P* value of 5 × 10^-8^ might overlook some real causal SNPs (13). Additionally, the vast majority of GWAS-identified SNPs are located within noncoding genomic regions; their causal relationships and underlying mechanisms in the pathogenesis of GC remain largely elusive. Thus, a reevaluation of the GWAS data utilizing a hypothesis-driven approach based on biological relevance could be instrumental in deciphering the specific causal roles and mechanistic pathways by which these GWAS SNPs influence the development of GC.

Ubiquitylation and deubiquitylation represent key reversible modalities of protein posttranslational modification (PTM), playing pivotal roles in regulating protein stability and activity under physiological or pathological conditions (14–16). Disruption of this equilibrium can lead to a wide range of diseases, including cancer and inflammatory disorders. As key components of the ubiquitin proteasome system, the deubiquitinase (DUBs) family, ubiquitin-specific protease (USP) has been implicated in numerous cellular functions, encompassing cell-cycle progression, gene transcription, DNA repair, apoptosis, and signal transduction (17–21). These processes collectively are crucial for organismal development and cellular differentiation, as well as tumorigenesis (22, 23). Previous studies have established that genetic variants in USP genes are associated with several types of diseases. For instance, the SNP rs2252257 in the promoter and enhancer regions of *USP18* is linked to familial neuromyelitis optica spectrum disorder (NMOSD), while SNPs rs361553, rs2252257, and rs5746523 demonstrate associations with sporadic NMOSD. Notably, individuals with the rs361553 T/T genotype exhibit a higher recurrence rate compared with those with C/T or C/C genotypes (24). Variants in *USP24* and *USP40* are implicated in the risk for late-onset Parkinson’s disease (PD), supporting the proposed role of ubiquitination pathways in PD pathogenesis (25). Nevertheless, the links between genetic variations of USPs and human cancers are less explored. Specifically, it is unclear whether and how genetic variants in USP genes are associated with GC susceptibility, and the underlying biological mechanisms and clinical implications of such susceptible SNPs in GC development are yet to be elucidated.

In the present study, we identified a GC-associated susceptibility locus, rs72856331 (G>A), located in the promoter region of *USP47* and delineated the molecular mechanism by which the GC risk–associated allele G of rs72856331 modulates the expression of *USP47*. This effect is mediated through altered chromatin binding affinity to GLI3, a novel oncogenic transcriptional regulator with prognostic potential in GC. Additionally, our investigation reveals that *USP47* upregulation potentially contributes to GC pathogenesis and tumor progression via the epithelial-mesenchymal transition (EMT) signaling pathway. Finally, we observed synergistic effects between the rs72856331 genotype and *USP47* expression in predicting GC relapse and patient survival. These findings underscore the clinical significance of this SNP as a potential risk stratification marker for the management of GC patients.

## Results

### Identification of a novel risk variant, rs72856331, associated with GC in the promoter region of USP47.

**Figure 1:**
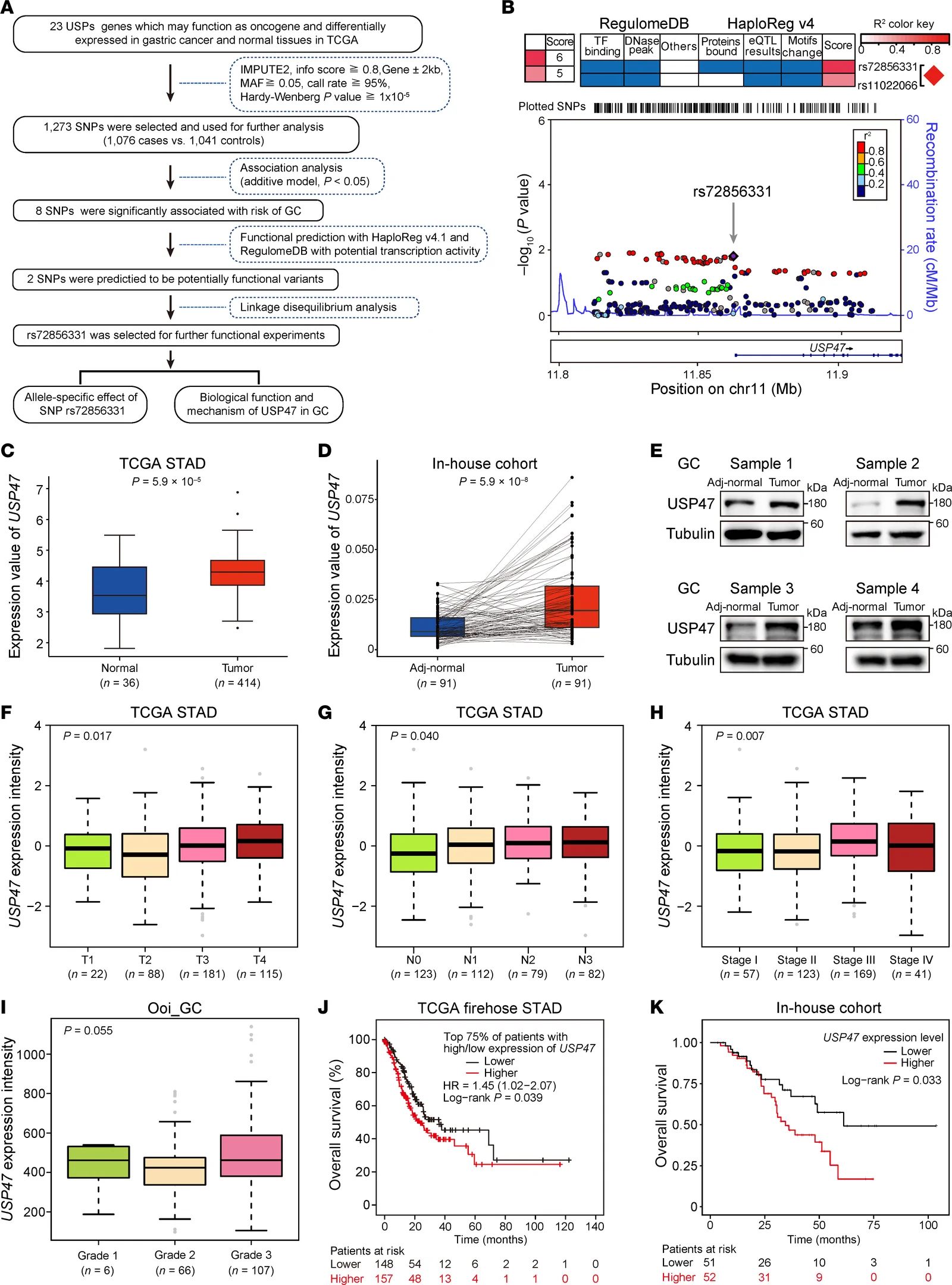
Identification of rs72856331 as a GC risk variant and correlation analysis of *USP47* expression and clinical features. (**A**) Flowchart of the study design. (**B**) LD plots for 2 GC risk-associated SNPs in Asian populations (Han Chinese in Beijing and Japanese in Tokyo from 1000 Genomes Project; top panel). Functional annotations include RegulomeDB and HaploReg, version 4.2, scores. Regional association plot shows SNP associations (−log_10_ *P*) within 50 kb of rs72856331/*USP47*. Logistic regression assessed SNP GC risk associations (*n* = 1,076 cases, 1,041 controls). rs72856331 (index SNP) is highlighted in purple; *r^2^* values of surrounding SNPs are color coded. Gene annotations are shown with directional arrows. (**C**) The TCGA STAD dataset showed significantly elevated *USP47* expression in GC tissues. *P* value assessed by a 2-tailed Student’s *t* test. (**D**) In-house dataset showed significant upregulation of *USP47* mRNA expression in GC tumors compared with matched adjacent normal tissues from the same individual by RT-qPCR. Data are represented as means ± SEM, analyzed using a 2-tailed Student’s *t* test. (**E**) Western blot indicated an apparent increase in USP47 protein levels in GC tumors compared with adjacent normal tissues from the same individual. (**F**–**H**) Elevated *USP47* levels are substantially associated with GC clinical features indicated by increased tumor invasiveness (*n* = 406) (**F**); lymph node metastasis (*n* = 394) (**G**); and advanced tumor stages (*n* = 390) (**H**) in TCGA STAD cohort. Statistical significance determined by Kruskal-Wallis H test. (**I**) Higher *USP47* levels show a moderate association with high-grade tumors in Ooi_Gc cohort, analyzed by Kruskal-Wallis H test. (**J** and **K**) Elevated *USP47* levels correlate with reduced overall survival time in TCGA STAD and in-house GC patient cohorts, as depicted by Kaplan-Meier curves. *P* values calculated using the log-rank test.

To discover novel SNPs and elucidate their potential functions in regulating the USP family genes that confer susceptibility to GC, we focused on 23 USPs characterized as oncogenes with elevated mRNA expression in GC tissues relative to normal gastric tissues, as determined by GEPIA, an interactive web server that analyzes RNA-Seq data from 9,736 tumors and 8,587 normal samples from the TCGA and GTEx projects (http://gepia.cancer-pku.cn/index.html) (26, 27) (Figure 1A and Supplemental Figure 1; supplemental material available online with this article; https://doi.org/10.1172/JCI179617DS1). We examined a genomic interval encompassing 2 kb upstream and downstream of these genes, extracting SNPs from our preexisting GWAS dataset of an Eastern Chinese cohort comprising 1,076 GC patients and 1,041 controls (Supplemental Table 1). Following whole-genome imputation and rigorous quality control with inclusion criteria, a set of 1,273 SNPs were selected for association analysis under an additive model adjusted for age and sex. Of these, 8 SNPs demonstrated a significant association with GC risk after being corrected for false discoveries using the Bayesian false-discovery probability (BFDP) test (28) (Supplemental Table 2). Furthermore, a functional annotation analysis by using an aggregation of HaploReg, version 4, and RegulomeDB was conducted to identify potential causal variants, revealing that 2 SNPs, rs72856331 and rs11022066, exhibited high functional scores (rs72856331: functional score 6, OR = 0.78, 95% CI = 0.64-0.95, *P* = 0.015; rs11022066: functional score 5, OR = 0.79, 95% CI = 0.65-0.96, *P* = 0.017, Figure 1B and Supplemental Tables 3 and 4). Finally, linkage disequilibrium (LD) analysis indicated high LD between these 2 SNPs (*r^2^* > 0.8, Figure 1B). To assess the frequency of rs72856331 across different populations, we examined the minor allele frequency (MAF) of rs72856331 using the Ensembl genome browser dataset (https://asia.ensembl.org/index.html). We observed slight variations in MAF across populations: 0.17 in East Asians, 0.19 in Europeans, and 0.09 in Americans (Supplemental Figure 2). In our study, the MAF of rs72856331 in the Eastern Chinese population was 0.17, which aligns with the MAF reported for East Asian populations in Ensembl, thus confirming the accuracy of our genotyping. Taken together, both statistical and functional annotation analyses suggested rs72856331, located in the promoter region of *USP47*, as a putative causal variant due to its higher functional scores.

### USP47 as an oncogene with prognostic potential in GC.

We next sought to elucidate the functional significance of *USP47*, the putative target of rs72856331, in relation to GC susceptibility. Differential gene expression analysis revealed a marked upregulation of *USP47* in tumor tissues, compared with normal tissues using The Cancer Genome Atlas Stomach Adenocarcinoma (TCGA STAD) cohort and 2 online datasets (UALCAN, a comprehensive and interactive web resource for analyzing cancer OMICS data [https://ualcan.path.uab.edu], and GEPIA) (Figure 1C and Supplemental Figure 3, A and B). To validate the results, we evaluated the mRNA levels of *USP47* in 91 paired GC tissue samples from an in-house dataset and our ongoing in-house early-onset (FUEOGC) and late-onset (FULOGC) GC cohorts, which demonstrated marked higher *USP47* expression in cancer specimens relative to the adjacent normal tissues (Figure 1D and Supplemental Figure 3, C and D). In line with this, elevated USP47 protein levels were observed in GC specimens compared with the corresponding adjacent normal tissues (Figure 1E), implying a potential oncogenic role for *USP47* in GC.

Next, we performed clinical correlation and prognosis analyses to investigate whether *USP47* upregulation correlates with GC progression and indicates any clinical relevance. The results showed that elevated expression levels of *USP47* are markedly correlated with increased tumor invasiveness, lymph node metastasis, and advanced tumor stages in the TCGA and Ooi GSE15459 datasets (https://www.ncbi.nlm.nih.gov/geo/query/acc.cgi?acc=gse15459), with a borderline significance in high-grade tumors (29) (Figure 1, F–I). We performed Dunn’s post hoc tests for pairwise comparisons, which revealed pronounced differences in *USP47* expression across various GC subgroups, including tumor invasiveness, lymph node metastasis, tumor stage, and tumor grade (Supplemental Figure 4). Moreover, patients with higher *USP47* mRNA expression exhibited reduced overall survival in both the public TCGA STAD, our in-house GC cohort, and multiple Gene Expression Omnibus (GEO) cohorts (Figure 1, J and K, and Supplemental Figure 5). Collectively, these observations illustrate that *USP47* is a GC-relevant gene with potential prognostic value for assessing GC risk.

### Effect of USP47 on GC cell proliferation and in vivo tumor growth and metastasis.

**Figure 2:**
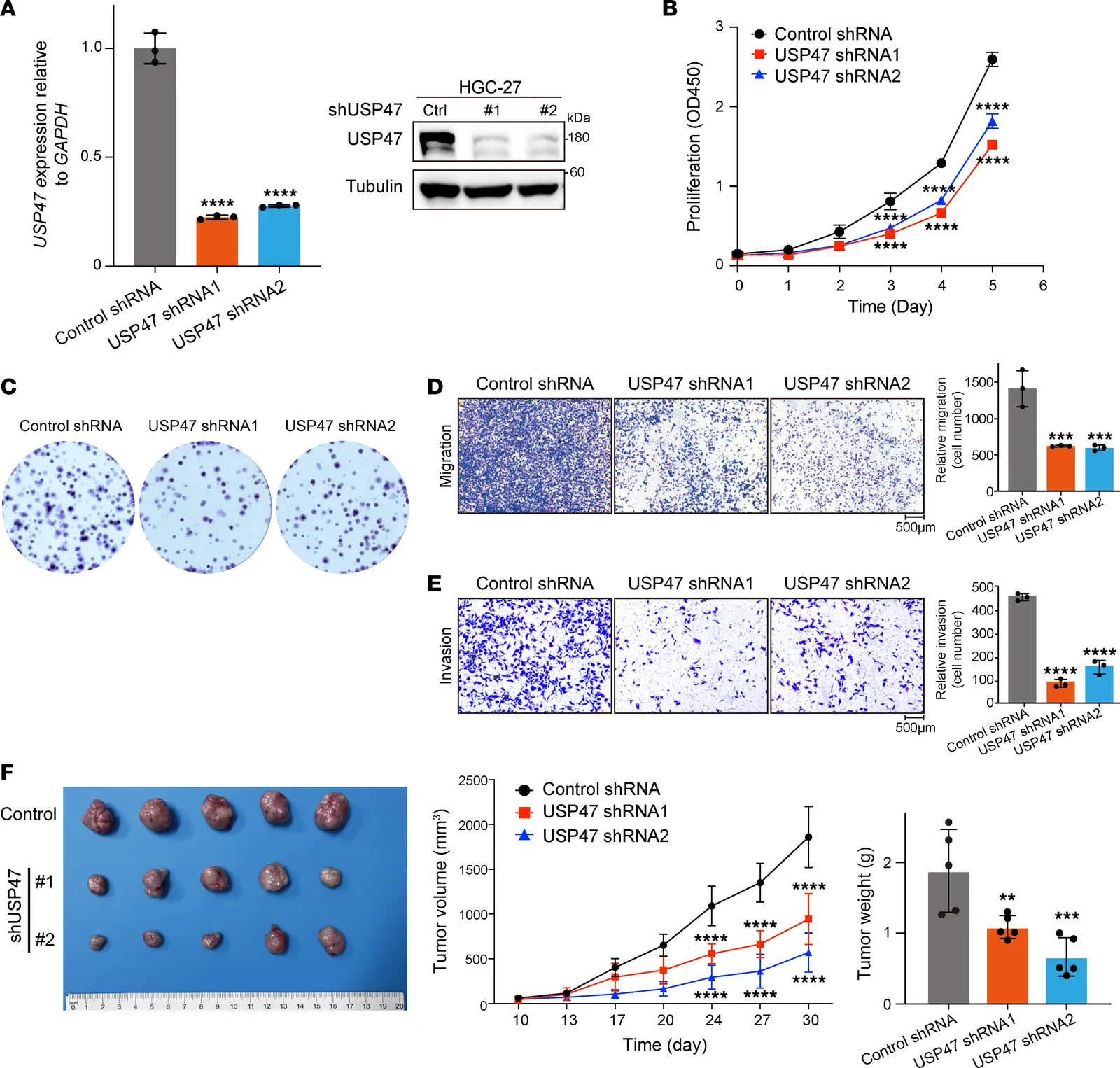
USP47 downregulation compromises cancer-related phenotypes. (**A**) qPCR and Western blot analysis of *USP47* RNA and protein levels in HGC-27 cells after *USP47* knockdown with 2 independent shRNAs. (**B**) CCK-8 assay showing suppressed cell growth in HGC-27 cells with *USP47* knockdown versus control shRNA. (**C**–**E**) Colony formation (**C**), migration (**D**), and invasion (**E**) assays in HGC-27 cells with *USP47* knockdown. Scale bars: 500 μm. Migrated and invaded cells were quantified. Data are represented as means ± SD from 3 independent replicates (**A**–**E**); significance determined by 1-way ANOVA with Holm-Šidák multiple-comparison test for panels **A**, **D**, and **E**, or by 2-way ANOVA and Holm-Šidák post hoc test for panel **B**. (**F**) Xenograft tumor images, growth curves, and weights from nude mice injected with HGC-27 cells (*USP47* knockdown vs. control) after 4 weeks (*n* = 5 per group). Significance assessed by 2-way ANOVA and Holm-Šidák post hoc test for growth curves and 1-way ANOVA with Holm-Šidák multiple comparison test for tumor weights. *P < 0.05; **P < 0.01; ***P < 0.001; ****P < 0.0001.

To investigate the biological impact of USP47 on GC, we established stable *USP47*-knockdown cell lines using 2 independent shRNAs in HGC-27, GT38, SNU719, and SNU668 cell lines, as well as USP47-overexpressing cell lines by transfecting USP47 expression plasmids into HGC-27 and GT38 cells. The efficiency of knockdown and overexpression was verified by quantitative reverse-transcription PCR (RT-qPCR) and Western blotting (Figure 2A, Supplemental Figure 6A, and Supplemental Figure 7A). *USP47* knockdown dramatically impaired cell proliferation and clone-forming ability across all 4 GC cell lines (Figure 2, B and C, and Supplemental Figure 6, B and C), while USP47 overexpression markedly enhanced these phenotypes (Supplemental Figure 7, B–D). We further assessed migration and invasion capabilities using chamber assays, with and without Matrigel. Migration and invasion were substantially diminished in USP47-depleted GC cells (Figure 2, D and E, and Supplemental Figure 6, D and E), whereas these abilities were markedly enhanced in USP47-overexpressing cells (Supplemental Figure 7, E–H). These results strongly support the role of USP47 in promoting aggressive behaviors in GC cells.

To explore USP47’s tumor-promoting capacity in vivo, we generated xenograft mouse models by implanting HGC-27 and GT38 cells with USP47 depletion or overexpression into immunodeficient mice. Consistent with in vitro results, *USP47* knockdown drastically inhibited tumor growth in vivo (Figure 2F and Supplemental Figure 6F). Conversely, USP47 overexpression markedly accelerated tumor growth, yielding larger tumors and higher weights (Supplemental Figure 7, I and J). Additionally, we examined USP47’s role in metastasis using tail-vein injection of HGC-27 and GT38 cells into NOD/SCID mice. *USP47* knockdown dramatically reduced metastasis, as evidenced by lower bioluminescence intensity (Supplemental Figure 6, G and H). In contrast, USP47-overexpressing cells led to a marked increase in lung metastases compared with controls (Supplemental Figure 7, K and L). Taken together, our results reveal an oncogenic function of *USP47* in promoting GC cell proliferation and metastasis, both in vitro and in vivo.

### The promoter variant rs72856331 modulates USP47 expression in an allele-specific manner.

**Figure 3:**
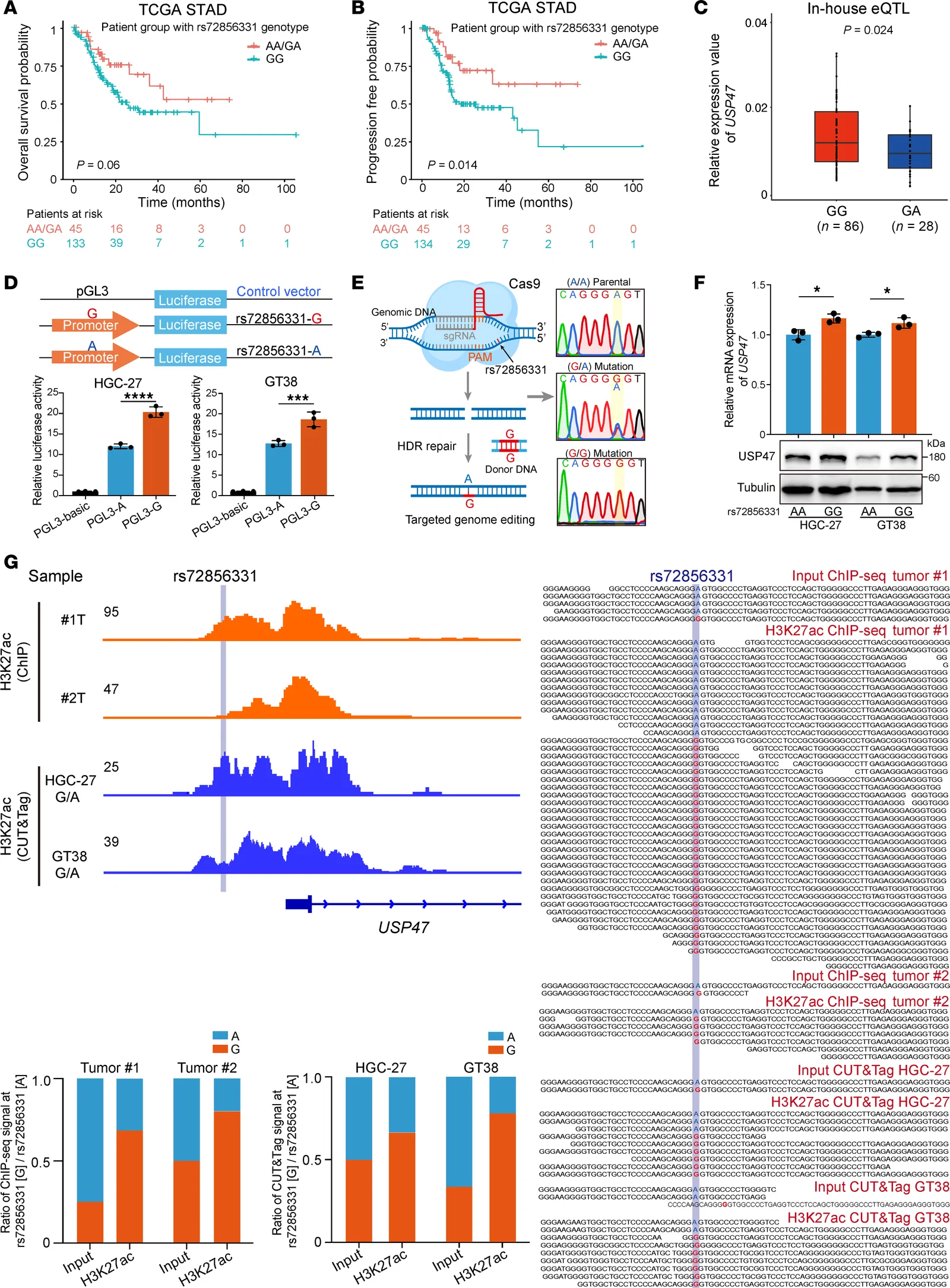
The rs72856331-containing region has an allele-specific promoter activity. (**A** and **B**) TCGA STAD cohort analysis showed GC risk-associated GG genotype carriers had shorter survival and higher progression risk than GA/AA carriers. Statistical significance determined by a log-rank test. (**C**) rs72856331 risk allele G correlated with higher *USP47* expression in our in-house cohort of 114 GC tissues. (**D**) Dual luciferase reporter assay in HGC-27 cells comparing empty vector and rs72856331 risk allele G or nonrisk allele A (*n* = 3). (**E**) Schematic of CRISPR/Cas9-mediated genome editing and Sanger sequencing of parental and converted rs72856331 genotypes in HGC-27 cells. (**F**) *USP47* expression in mutated and parental HGC-27 and GT38 cells analyzed by RT-qPCR and Western blot (*n* = 3). (**G**) Allele-specific enrichment of H3K27ac at rs72856331 in GC cells, assessed by CUT&Tag in edited heterozygous HGC-27-GA and GT38-GA cells and ChIP-Seq in clinical GC samples. Histograms show the ratio of sequencing reads at the risk allele G versus the nonrisk allele A across samples. Data are represented as means ± SD. Statistical significance was calculated using a 2-tailed Student’s *t* test for panels **C** and **F**, or 1-way ANOVA with Holm-Šidák multiple comparison test for panel **D**. *P < 0.05; ***P < 0.001; ****P < 0.0001.

Considering the marked association of rs72856331 with GC susceptibility, we investigated its correlation with disease progression and patient survival. Analysis of a large GC cohort revealed that patients with the homozygous GG genotype at rs72856331 had a significantly shorter overall survival compared with those with AA/GA genotypes (Figure 3A). Furthermore, individuals carrying the homozygous GG risk genotype exhibited an increased probability of cancer progression (Figure 3B), highlighting the significance of the G variant at rs72856331 in both GC risk and disease progression, as well as its impact on clinical outcomes.

To determine whether rs72856331 variants are associated with the expression of its linked gene, *USP47*, we performed an expression quantitative trait locus (eQTL) analysis of an in-house cohort of gastric tissues using quantitative RT-qPCR. This analysis identified a significant correlation between the GC risk allele G at rs72856331 and elevated *USP47* mRNA levels (Figure 3C). To further explore the regulatory mechanisms of this noncoding SNP, dual-luciferase reporter assays were conducted with rs72856331 G or A allele-centered sequences. The results showed that the rs72856331 region harboring the G allele had considerably higher luciferase activity than the A allele in HGC-27 and GT38 GC cell lines (Figure 3D). These data suggest that the rs72856331-containing region may possess the transcriptional regulation activity in an allele-specific manner.

To assess whether the rs72856331 region holds any regulatory activity in cells, we referenced the Encyclopedia of DNA Elements (ENCODE) database (https://www.encodeproject.org) for chromatin location data and found that rs72856331 resides in an open chromatin region particularly in GC cell lines, including BGC823, AGS, and MKN7 (Supplemental Figure 8). To investigate whether rs72856331 is involved in the regulation of endogenous *USP47* expression, we applied CRISPR/Cas9-mediated genome editing of rs72856331 in both HGC-27 and GT38 GC cell lines. In HGC-27 cells, we modified the genotype from homozygous A/A to G/A or G/G (Figure 3E), and in GT38 cells, we converted G/G into G/A or A/A (Supplemental Figure 9A). Subsequent analyses revealed considerably higher *USP47* mRNA and protein expression in the G/G genotype compared with A/A in both cell lines (Figure 3F). To examine the regulatory activities of rs72856331 variants in cells, we performed CUT&Tag using the antibody against an active epigenetic mark H3K27ac followed by next-generation sequencing in the obtained heterozygous HGC-27-GA and GT38-GA cell lines and observed a stronger enrichment of H3K27ac at G allele than A allele of rs72856331 (Figure 3G). Moreover, ChIP-Seq assays for H3K27ac in 2 clinical GC specimens confirmed greater H3K27ac enrichment at the rs72856331 region with a preference for the risk G allele (Figure 3G). Taken together, these findings demonstrated that the rs72856331 region containing the risk allele G exhibits higher transcriptional regulatory activity than the nonrisk A allele.

We next sought to examine the phenotypic effects across HGC-27 and GT38 cells with different rs72856331 genotypes and conducted cellular proliferation and migration assays. Our results revealed that the G/G genotype in both cell lines exhibited considerably enhanced proliferation, colony formation, migration, and invasion compared with the A/A genotype (Supplemental Figure 9, B–E). These findings suggest that the risk G allele plays a role in promoting GC progression.

### The risk allele G of rs72856331 is preferentially bound by transcription factor GLI3.

**Figure 4:**
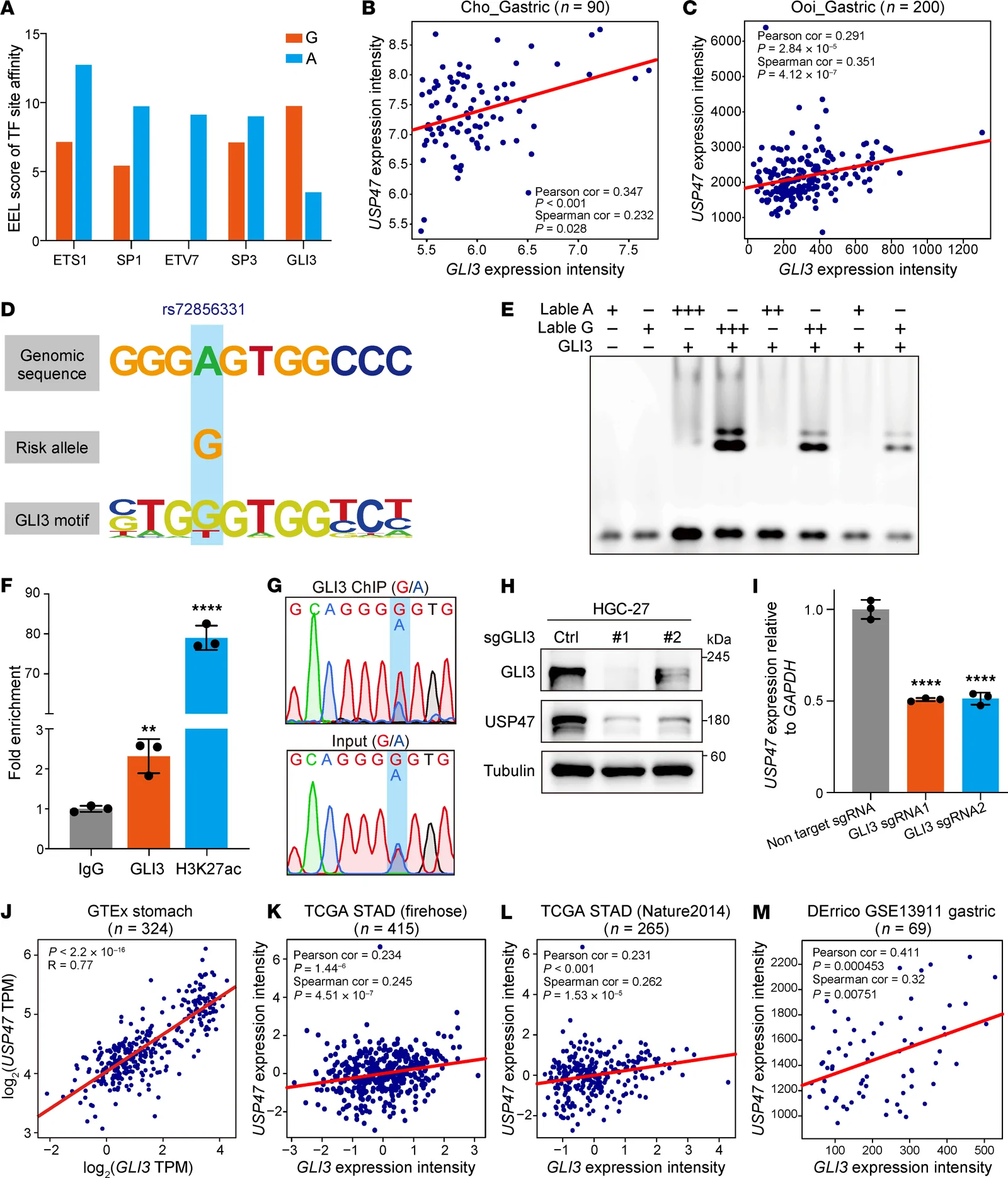
The risk allele G of rs72856331 enhances the binding ability of TF GLI3 to promote *USP47* expression. (**A**) DNA-binding affinity scores of 5 potential TFs toward the rs72856331-containing sequence calculated using the EEL algorithm. (**B** and **C**) Correlation between *GLI3* and *USP47* expression levels across multiple GC datasets. *P* values were calculated using Pearson’s correlation test. (**D**) rs72856331 resides within GLI3 DNA-binding motifs. (**E**) EMSA with purified GLI3 protein indicating a higher affinity for the rs72856331 G allele than the A allele. (**F**) ChIP assay in edited HGC-27-GA cells demonstrating GLI3 enrichment at rs72856331. IgG served as a negative control, while H3K27ac was included as a positive control (*n* = 3). Statistical significance was calculated using 2-tailed Student’s *t* test. **P < 0.01; ****P < 0.0001. (**G**) GLI3 prefers the binding to G allele than A allele at rs72856331 revealed by ChIP Sanger sequencing. (**H** and **I**) *USP47* was downregulated at both protein (**H**) and transcript levels (**I**) after partial *GLI3* knockout via CRISPR/Cas9 in HGC-27 GC cells (*n* = 3). Statistical significance was calculated by 1-way ANOVA with Holm-Šidák multiple-comparison test. ****P < 0.0001. (**J**–**M**) *USP47* expression positively correlated with *GLI3* in GTEx (**J**), TCGA STAD (**K** and **L**), and Derrico GSE13911_Gastric dataset (**M**). *P* values assessed by Pearson’s correlation test.

We hypothesized that variations at rs72856331 might affect *USP47* expression due to changes in the chromosomal association of DNA-binding transcription factors (TFs) that regulate transcription (30). To test this hypothesis, we identified 5 TFs with distinct DNA-binding affinities for the 2 rs72856331 variants by employing computational analysis using the enhancer element locator (EEL) algorithm and human TF DNA-binding motif data (31, 32). Among these TFs, GLI3 was the only one predicted to have a higher affinity for the risk G allele of rs72856331 (Figure 4A). Subsequent expression analysis showed a significant positive correlation between *USP47* and *GLI3*, but not with the other 4 TFs, in both Cho (33) and Ooi GSE15459 cohorts (29) (Figure 4, B and C, and Supplemental Figure 10A). This was further supported by the observed high similarity between the GLI3 motif and the G allele of rs72856331, as opposed to the A allele (Figure 4D). Collectively, we hypothesized that GLI3 is the most plausible TF in mediating the genetic regulatory effects of the rs72856331 locus.

We then conducted an electrophoretic mobility shift assay (EMSA) using purified GLI3 protein (Supplemental Figure 10B) to determine whether GLI3 directly binds to the rs72856331-containing DNA sequence. The results showed an increased intensity of shifted bands in a dose-dependent manner when the protein was incubated with the risk G allele of the SNP-containing sequence, whereas the A allele showed negligible shift (Figure 4E). In contrast, binding affinity assay using purified SP1 protein revealed no considerable differences between the 2 alleles (Supplemental Figure 10C). Consequently, we conclude that GLI3 directly and specifically interacts with the risk G allele of rs72856331.

To investigate whether GLI3 directly binds to the rs72856331-containing region in cells, we carried out a ChIP experiment followed by quantitative PCR, using a GLI3 antibody and IgG as a control. ChIP-qPCR results revealed a considerable enrichment of GLI3 and H3K27ac at the *USP47* promoter region in HGC-27 cells (Figure 4F). To determine whether GLI3 shows a preference for the G allele of rs72856331, we executed GLI3 ChIP in the CRISPR/Cas9-edited heterozygous HGC-27-GA cells. Sanger sequencing of the immunoprecipitated (IP) samples demonstrated a predominant presence of the risk G allele compared with the nonrisk A allele, whereas the levels of G and A alleles were similar in the input sample (Figure 4G). These findings collectively indicate that GLI3 specifically targets the risk G allele of rs72856331 at the *USP47* promoter.

We next sought to examine whether GLI3 regulates gene expression through the G allele of rs72856331 using the luciferase reporter assay. HGC-27 and GT38 cell lines were transfected with pGL3 luciferase constructs containing either the A or G allele of rs72856331, alongside pRL-SV40-Renilla vectors for normalization. These constructs were cotransfected with or without sgRNAs targeting *GLI3*. The results showed that the G allele considerably increased luciferase activity compared with the A allele in both cell lines. Notably, GLI3 depletion substantially reduced the luciferase signal in cells carrying the G allele compared with those with the A allele of rs72856331 (Supplemental Figure 10D). This suggests that GLI3 primarily modulates gene expression through recognizing the G allele of rs72856331.

To elucidate whether GLI3 regulates the expression of *USP47*, we depleted *GLI3* with independent sgRNAs and observed a decline in both RNA and protein levels of *USP47* (Figure 4, H and I). Moreover, the expression of *USP47* was positively correlated with that of *GLI3* across different large cohorts of clinical gastric tissue samples (Figure 4, J–M), indicating that GLI3 may also regulate the expression of *USP47* in the clinical setting.

### GLI3 promotes cancer-related phenotypes.

**Figure 5:**
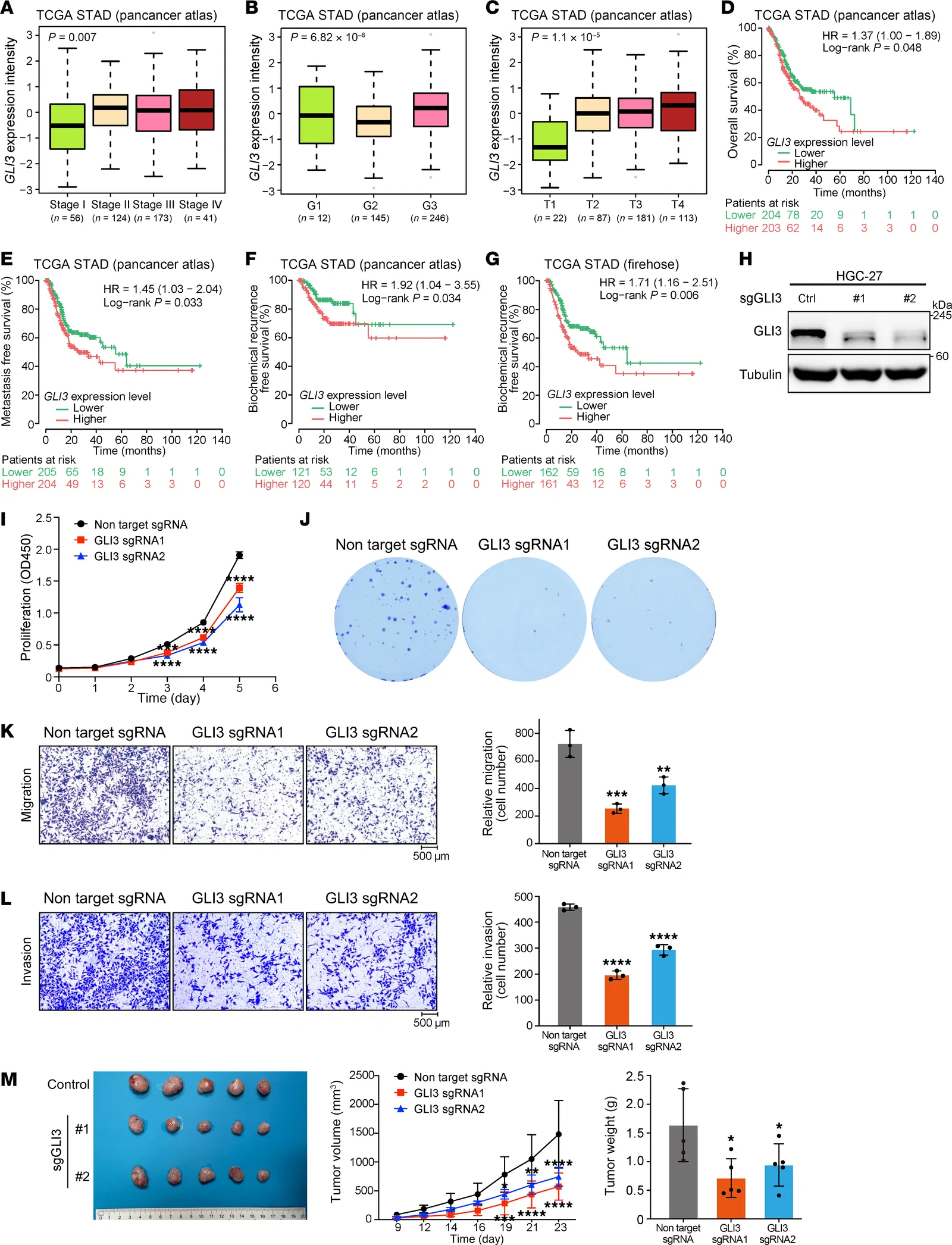
*GLI3* upregulation promotes cancer-related phenotypes and correlates with GC adverse clinical features. (**A**–**C**) Elevated *GLI3* levels are markedly associated with advanced tumor stages (*n* = 394), high-grade tumors (*n* = 403), and increased tumor invasiveness (*n* = 403) in TCGA STAD cohort, analyzed by Kruskal-Wallis H test. (**D** and **E**) Higher GLI3 levels correlate with shorter overall (**D**) and metastasis-free survival (**E**) in TCGA GC patients, as determined by a log-rank test. (**F** and **G**) Elevated *GLI3* levels predict biochemical recurrence in TCGA STAD patients, assessed by a log-rank test. (**H**) Western blot confirms CRISPR/Cas9-mediated GLI3 knockout in HGC-27 cells using 2 sgRNAs. (**I**) GLI3 promotes GC cell growth measured by CCK-8 assay in HGC-27 cells with GLI3-targeting or control sgRNA (*n* = 3). (**J**–**L**) Colony formation (**J**), migration (**K**), and invasion (**L**) assays for HGC-27 cells with GLI3-targeting or control sgRNA (*n* = 3). Scale bars: 500 μm. (**M**) Xenograft tumor images, growth curves, and weights from nude mice injected with HGC-27 cells (*GLI3* knockout vs. control) after 3 weeks (*n* = 5 per group). Significance assessed by 2-way ANOVA and Holm-Šidák post hoc test for growth curves and 1-way ANOVA with Holm-Šidák multiple comparison test for tumor weights. *P < 0.05; **P < 0.01; ***P < 0.001; ****P < 0.0001. Data are represented as means ± SD. Statistical significance was calculated using 2-way ANOVA and Holm-Šidák post hoc test for panel **I** or 1-way ANOVA with Holm-Šidák multiple-comparison test for panels **K** and **L**.

Analysis of TCGA STAD expression data with clinical follow-up outcomes revealed a positive correlation between *GLI3* expression and the progression of human GC. Tumors characterized by higher stages, greater grades, and more extensive lymphatic metastasis exhibited elevated levels of *GLI3* expression, as confirmed by Kruskal-Wallis and Dunn’s post hoc tests (Figure 5, A–C, and Supplemental Figure 10, E–G). Additionally, Kaplan-Meier survival analysis indicated that patients with higher *GLI3* expression experienced reduced overall survival and had increased incidences of metastasis and biochemical recurrence (Figure 5, D–G).

Therefore, to investigate the potential oncogenic role of *GLI3* in GC development, we employed CRISPR/Cas9 technology to generate *GLI3* knockouts in 4 GC cell lines (HGC-27, GT38, SNU719, and SNU668) with 2 separate sgRNAs. The efficiency of these knockouts was validated through Western blotting analysis (Figure 5H and Supplemental Figure 11A). The absence of GLI3 led to a marked reduction in cell growth and colony-formation capabilities in all 4 GC cell lines (Figure 5, I and J, and Supplemental Figure 11, B and C), as well as diminished cell migration and invasion abilities (Figure 5, K and L, and Supplemental Figure 11, D and E). To assess the impact of *GLI3* on tumor growth in vivo, we established a xenograft mouse model using HGC-27 and GT38 cells transduced with GLI3-specific or control sgRNAs. The results showed a substantial decrease in tumor growth in mice implanted with *GLI3*-knockout cells (Figure 5M and Supplemental Figure 11F). Additionally, to investigate GLI3’s role in metastasis, we injected GLI3-knockout HGC-27 and GT38 cells into the tail veins of NOD/SCID mice. GLI3 knockout resulted in a considerably reduction in lung metastasis, indicating its role in promoting metastatic potential (Supplemental Figure 11, G and H).

Taken together, our results indicate that *GLI3* possesses an oncogenic potential to promote GC cell proliferation and invasiveness in vitro and enhances tumor growth and metastasis in vivo. Its upregulation markedly correlates with the development and clinical severity of GC. These findings underscore the importance of GLI3 as a critical TF in influencing the regulatory effect of the rs72856331 locus at the *USP47* promoter, thereby affecting GC susceptibility and progression.

### USP47 regulates the EMT signaling pathway through stabilization of Snai1.

**Figure 6:**
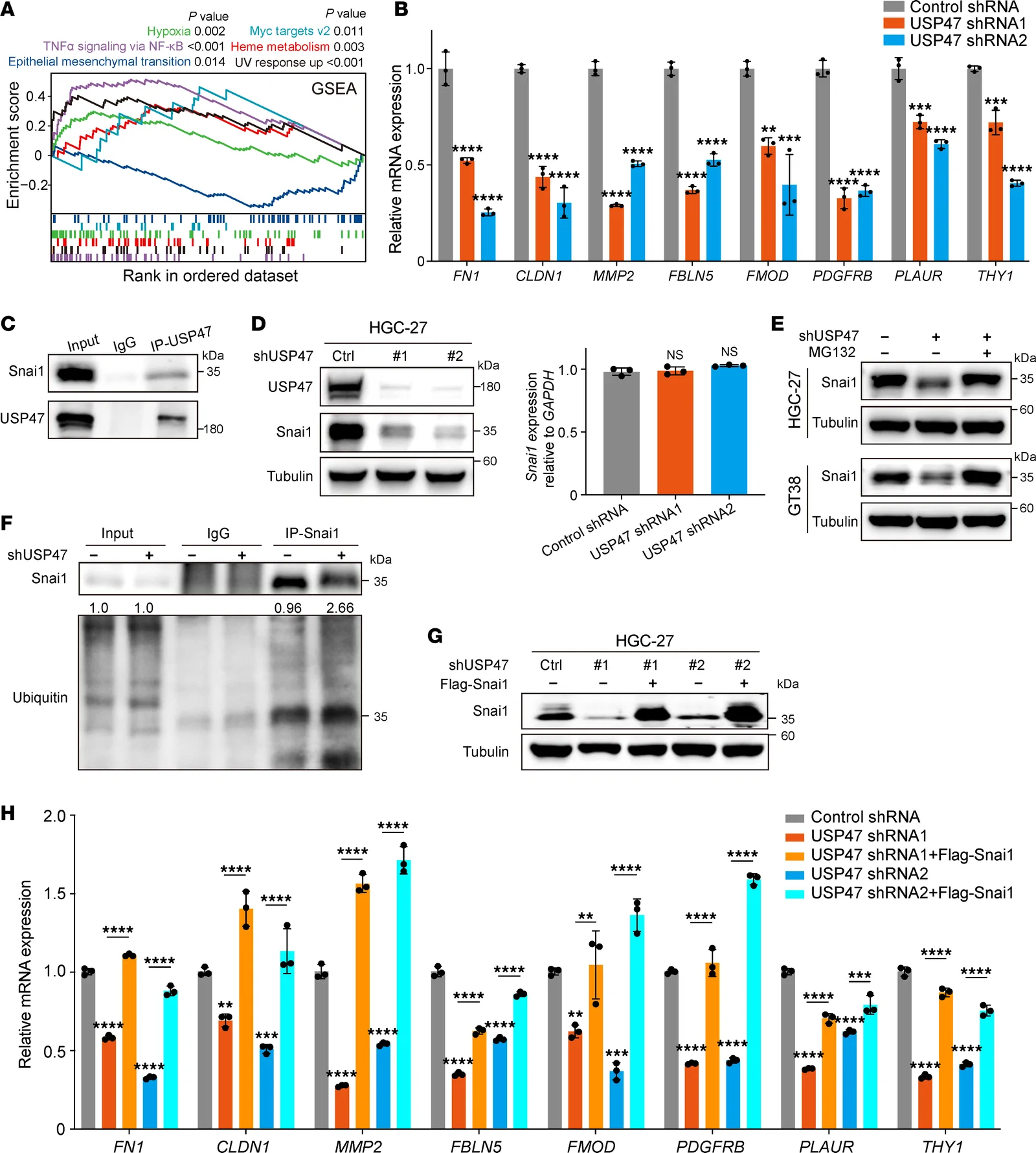
The EMT signaling pathway is regulated by USP47 via stabilization of Snai1 in GC cells. (**A**) GSEA was performed on differentially expressed genes from RNA-Seq of HGC-27 cells with *USP47* knockdown versus control. (**B**) RT-qPCR validation of EMT-related gene expression in HGC-27 cells with *USP47* knockdown and control or *USP47* shRNAs (*n* = 3). (**C**) Whole-cell lysates from HGC-27 cells were subject to IP with α-USP47 followed by immunoblotting (IB) with α-USP47 or α-Snai1 antibody. (**D**) USP47 regulates Snai1 stability. *USP47* knockdown decreased Snai1 protein levels. The relative mRNA levels of *Snai1* were quantified using RT-qPCR (*n* = 3). (**E**) *USP47* knockdown promotes Snai1 degradation in HGC-27 and GT38 cells, mitigated by MG132 proteasome inhibition. (**F**) *USP47* knockdown enhances Snai1 ubiquitination in HGC-27 cells, confirmed by co-IP and IB with antiubiquitin. (**G**) Western blot results confirming Snai1 levels in control or *USP47* shRNA transduced HGC-27 cells with or without Snai1 expression restoration. (**H**) Downregulation of EMT-related genes upon *USP47* knockdown was rescued by Snai1 restoration. Bar graph shows relative mRNA expression of key EMT genes in HGC-27 cells with control or *USP47*-specific shRNAs, with or without Snai1 restoration (*n* = 3). Data are represented as means ± SD. Statistical significance was calculated using 1-way ANOVA with Holm-Šidák multiple comparison test for panels **B**, **D**, and **H**. **P < 0.01; ***P < 0.001; ****P < 0.0001.

To explore the mechanisms by which USP47 regulates the malignant phenotypes of GC, we performed RNA-Seq analysis in both control and the stable knockdown cell lines transduced with shRNAs against *USP47* to identify the affected signaling pathways (Supplemental Figure 12A). Gene Ontology (GO) analysis of the transcriptomic profiling data indicated that the EMT signaling pathway, a key player in cancer development, is predominantly enriched in the downregulated genes, while other considerably altered pathways, including TNF-α and hypoxia pathways, are more mainly upregulated in *USP47*-knockdown cells (Figure 6A). Subsequent RT-qPCR validation of key EMT-related genes, including *FN1*, *CLDN1*, *MMP2*, *FBLN5*, *FMOD*, *PDGFRB*, *PLAUR*, and *THY1*, corroborated the RNA-Seq findings (Figure 6B). Additionally, in CRISPR/Cas9-edited HGC-27 and GT38 cells, the G/G genotype at rs72856331 was associated with upregulation of these EMT-related genes compared with the A/A genotype, suggesting a more invasive and metastatic phenotype (Supplemental Figure 12B). These results underscore the role of USP47 in promoting EMT signaling and suggest that the rs72856331 G allele enhances this effect, contributing to the aggressive behavior of GC cells.

Given the known interaction between USP47 and Snai1, a key modulator of EMT (34, 35), we hypothesized that USP47 might induce EMT activation by regulating deubiquitination and protein stability of Snai1 in GC. Co-immunoprecipitation (Co-IP) assays using a USP47 antibody confirmed a physical interaction between USP47 and Snai1 (Figure 6C). In HGC-27 cells, USP47 knockdown reduced Snai1 protein levels without affecting its mRNA expression (Figure 6D), indicating a posttranslational regulation of Snai1 by USP47. The decrease in Snai1 protein levels caused by knocking down *USP47* was rescued upon treatment with the proteasome inhibitor MG132 (Figure 6E). Moreover, the levels of ubiquitinated Snai1 were evidently higher in USP47-depleted cells, indicating the role of USP47 in deubiquitinating and stabilizing Snai1 (Figure 6F).

To investigate the dynamic control of Snai1 protein levels by USP47, we established the acute degradation cells by introducing the FKBP12^F36V^ degradation tag N-terminally at the endogenous *USP47* locus in HGC-27 cells (36). Mirroring the effects of *USP47* knockdown, its degradation via 12- and 24-hour treatments with dTAG-13 resulted in a noticeable reduction in Snai1 protein levels (Supplemental Figure 12E). Additionally, in the presence of protein translation inhibitor cycloheximide (CHX), the degradation of USP47 markedly accelerated the protein turnover of Snai1 in HGC-27 cells (Supplemental Figure 12F). Consistently, the depletion of USP47 by shRNAs enhanced Snai1 turnover in both HGC-27 and GT38 cells (Supplemental Figure 12G). Collectively, these results support that USP47 plays a crucial role in regulating the stability of Snai1 protein.

To understand the implication of Snai1 on GC development, we established *Snai1*-knockdown HGC-27 cell lines using 2 distinct *Snai1*-specific shRNAs, with knockdown efficacy verified by Western blotting and RT-qPCR (Supplemental Figure 13A). Similar to the influence of USP47 depletion on GC tumor cellular phenotype, Snai1 deficiency profoundly inhibited cell proliferation, colony-forming capability, cell migration, and invasion (Supplemental Figure 13, B–E).

**Figure 7:**
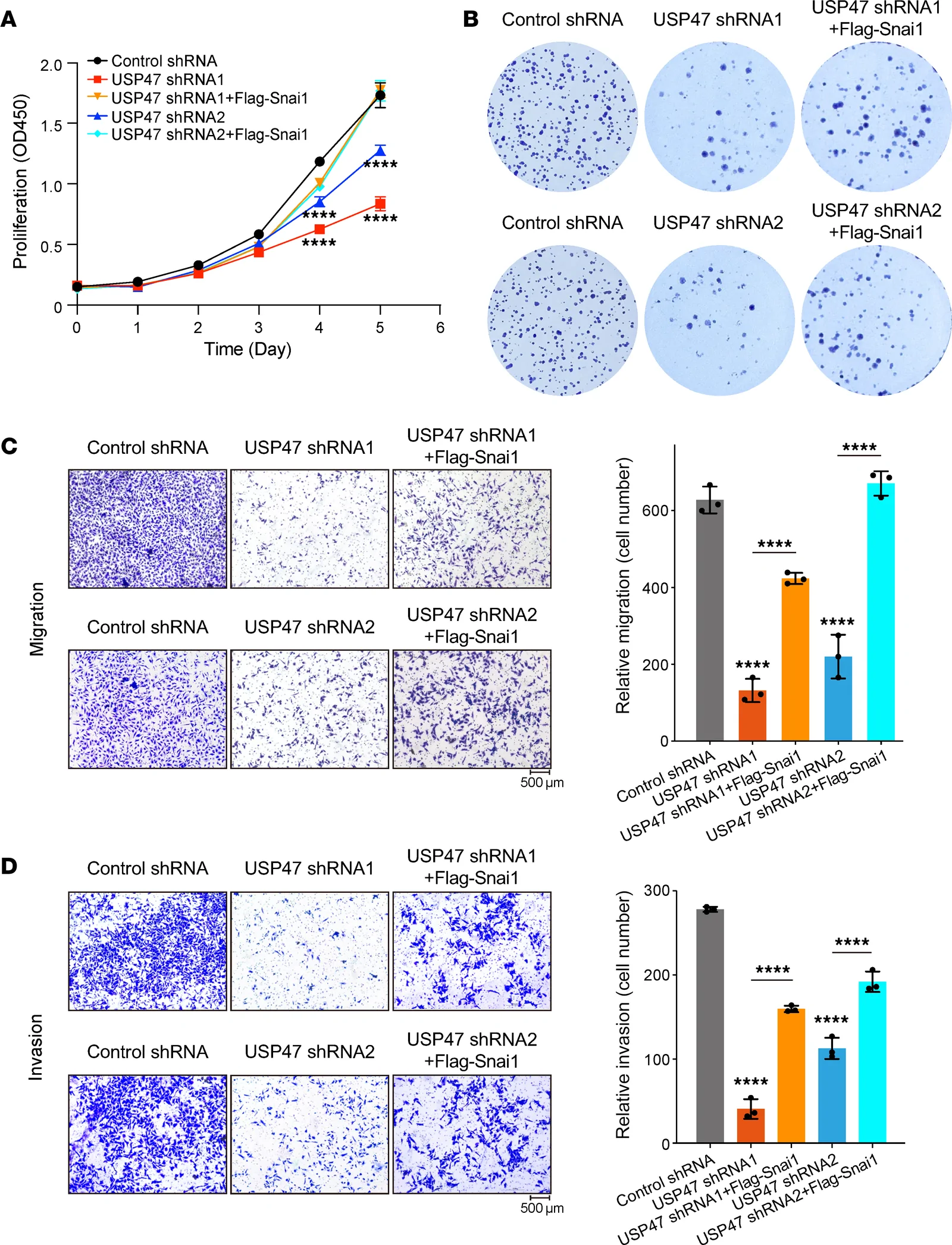
USP47-mediated stabilization of Snai1 is involved in the cancer-related phenotypes. (**A** and **B**) Overexpression of Snai1 rescued *USP47* knockdown-induced inhibition of cell proliferation indicated by cell growth (**A**) and clone-forming capability (**B**) in HGC-27 cells (*n* = 3). (**C** and **D**) Overexpression of *Snai1* rescued *USP47* knockdown-compromised inhibition of cell migration (**C**) and invasion (**D**) in HGC-27 (*n* = 3). Data are represented as means ± SD. Statistical significance was calculated using 2-way ANOVA and Holm-Šidák post hoc test for panel **A**, or 1-way ANOVA with Holm-Šidák multiple comparison test for panels **C** and **D**. ***P < 0.001; ****P < 0.0001.

Next, to determine whether USP47 promotes GC development through maintaining Snai1 levels, we induced the ectopic expression of Snai1 in USP47-depleted cells (Figure 6G and Supplemental Figure 14A). Snai1 overexpression partially rescued the GC tumor cellular phenotypes, including proliferation, colony formation, cell migration, and invasion (Figure 7 and Supplemental Figure 14, B–E). Additionally, the expression of EMT pathway–related genes, which were downregulated upon *USP47* knockdown as described above, was restored by Snai1 overexpression (Figure 6H and Supplemental Figure 14F). These findings implicate Snai1 in regulating the growth and invasiveness of GC, highlighting the crucial role of USP47 in stabilizing Snai1 and thus promoting EMT signaling and subsequent invasion of GC cells.

### Synergistic prognosis effects of rs72856331 genotype and USP47 expression on GC clinic.

**Figure 8:**
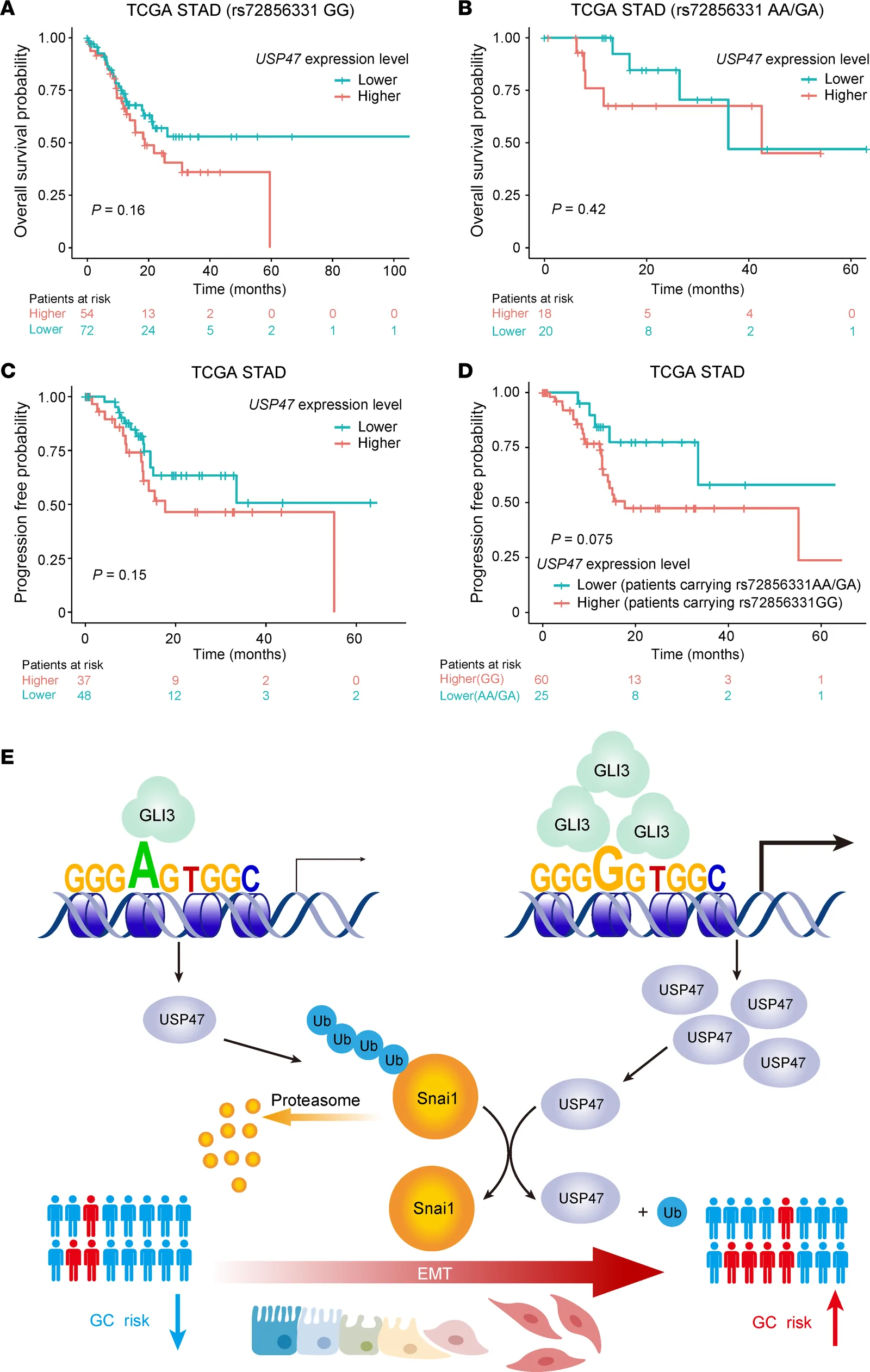
Synergistic effect of rs72856331 genotype and *USP47* expression on GC patient prognosis. (**A** and **B**) Kaplan-Meier survival curves for TCGA STAD GC patients, stratified by *USP47* expression levels and rs72856331 genotypes, show significant prognostic value for *USP47* in patients with the GG genotype (**A**) compared with AA/GA genotypes (**B**). (**C** and **D**) *USP47* expression alone shows no prognostic significance for progression-free probability (**C**), but high *USP47* levels in patients with the rs72856331 GG genotype correlate with decreased progression-free survival (**D**). (**E**) Model for the functional mechanism of the GC risk-associated promoter variant rs72856331 of *USP47*. The GC susceptibility locus rs72856331 resides in the *USP47* promoter region, and risk allele G correlates with the upregulation of *USP47* expression. Mechanically, the TF GLI3 exhibits a preference for binding to the risk allele G–containing promoter sequence, thereby promoting *USP47* expression. USP47 upregulation in turn stabilizes Snai1, therefore activating the EMT signaling pathway and contributing to cancer-related phenotypic changes. *P* values assessed by log-rank test for panels **A**–**D**.

Considering the association of the rs72856331 risk allele with elevated *USP47* mRNA levels and the link between *USP47* upregulation and GC progression, we next assessed whether the rs72856331 genotype and *USP47* expression together influence GC clinical prognosis. We employed Kaplan-Meier analysis to evaluate the impact of *USP47* expression on overall survival and progression-free probability in GC patients with different rs72856331 genotypes. Our analysis revealed that patients with the GG genotype of rs72856331, when combined with higher *USP47* mRNA levels, exhibited significantly shorter overall survival compared with those with AG or AA genotypes (Figure 8, A and B). Moreover, while *USP47* expression alone was not significantly linked to progression-free probability, the combination of the GG genotype and high *USP47* expression correlated with a substantially lower progression-free probability (Figure 8, C and D). These findings indicate that the risk GG genotype at rs72856331 and high *USP47* expression synergistically deteriorate GC clinical outcomes, underscoring the value of combining genetic and transcriptomic data for more accurate prognostic assessments in GC.

## Discussion

In recent years, GWAS have identified a subset of common variants associated with GC pathogenesis. However, stringent *P* value thresholds in these studies may have led to the omission of genuine susceptibility loci. A reevaluation of GWAS datasets, employing a hypothesis-driven approach with less stringent *P* value criteria, could help identify additional loci and elucidate their functional mechanisms underpinning GC susceptibility. USPs, a key group of DUB family members, have been implicated in tumor progression, with some emerging in development as cancer therapeutic targets (37–39). However, the associations between USP genetic variations and GC risk remains largely unexplored. In this study, we identified a SNP (rs72856331) associated with GC risk, located within the promoter region of *USP47*. We demonstrated that the risk allele G of rs72856331 correlates with enhanced promoter activity, leading to the upregulation of *USP47* expression. Mechanistically, the TF GLI3 exhibits a preference for binding to the risk allele G-containing promoter sequence, thereby promoting *USP47* expression. This elevation in *USP47* expression contributes to cancer-related phenotypes through stabilizing Snai1, which in return regulates the EMT signaling pathway. These findings together provide insights into the mechanisms by which genetic variations within promoter regions could influence GC-associated phenotypes by modulating the expression of its linked gene, thereby improving our understanding of the molecular mechanisms underpinning GC.

Most disease-associated SNPs identified by GWAS are located within noncoding regions of the genome. Several of these SNPs have been demonstrated to influence TF binding in an allele-specific manner, thereby modulating the expression of corresponding genes (40–42). In our current study, 8 SNPs within the USP family were found to be significantly associated with GC risk. Among them, rs72856331 emerged as the most prominent susceptibility locus for GC, located in the promoter region of *USP47* (OR = 0.78, 95% CI = 0.64-0.95, *P* = 0.015). Leveraging bioinformatics tools for predictive analysis, rs72856331 was also identified as possessing the greatest potential for biological functionality among the SNPs examined. This insight prompted a thorough investigation into the biological relevance and molecular mechanisms underlying rs72856331 and GC risk association, particularly focusing on its role in promoter regulatory activity and subsequent gene-expression modulation within the context of GC pathogenesis.

We showed multiple lines of evidence that rs72856331 may regulate *USP47* expression in an allele-specific manner. First, functional annotation analyses using RegulomDB and Haploreg, version 4, indicated that rs72856331 is located in a DNase I–hypersensitive site, implying potential effects on chromatin opening and TF DNA-binding alteration. Our subsequent eQTL analysis utilizing in-house stomach tissue data demonstrates a strong correlation between the GC risk allele G at rs72856331 and the elevated mRNA levels of *USP47*. Secondly, luciferase reporter assays indicate that the risk allele G exhibits higher activity compared with the nonrisk allele T, consistent with the above eQTL finding. A major challenge in mechanistically elucidating the impact of common, noncoding SNPs is providing direct evidence of their influence on gene expression. We thus particularly employed a modified CRISPR/Cas9-mediated genome-editing technique, as previously described (40, 43), to alter the genotype of rs72856331 in GC cell lines. In HGC-27 cells, the genotype was changed from A/A to G/A or G/G, while in GT38 cells, it was transitioned from G/G to G/A or A/A. Cells with the G/G genotype exhibited increased *USP47* expression at both the mRNA and protein levels compared with those with the A/A genotype. Moreover, the G/G genotype showed the phenotypic effects on promoting proliferation, colony formation, migration, and invasion in both cell lines, confirming the role of the risk G allele in driving GC progression. The rs72856331 G/A genotype cell line was subsequently subjected to allele-specific chromatin status analysis to further stress the direct impact of the variants on gene expression. We thus observed an enhanced enrichment of the active epigenetic mark (H3K27ac) at the rs72856331-G allele compared with the rs72856331-A allele. Intriguingly, ChIP-Seq analysis of GC clinical tissues from 2 patients corroborated these findings, further indicating greater transcriptional regulatory activity of the rs72856331-G allele in a clinical context. Moreover, the locus-responsible TF GLI3 also indicated allele-specific binding to the rs72856331-containing sequence in the HGC-27 AG mutant cells. In line with this, depletion of GLI3 led to reduced expression of the target gene *USP47*. These data compellingly suggest that rs72856331 influences GC by directly modulating GLI3 binding in an allele-specific manner, thereby regulating *USP47* expression.

*USP47* has been implicated in several critical cellular processes and identified as a key player in several types of cancers (44–48). Nevertheless, to date, no study has conclusively demonstrated the impact of any genetic variant within the gene regulatory region of *USP47* on its expression or functionality. Herein we identified *USP47* as a plausible target gene of the rs72856331 GC risk locus. This is based on an eQTL analysis from our in-house GC dataset and further substantiated by a series of functional experiments. In particular, we provided comprehensive in vitro and in vivo evidence demonstrating the roles of *USP47* in promoting GC cell proliferation and invasiveness as well as its marked contribution to tumor growth and metastasis in the mouse models.

Identification of the responsible TFs acting through the noncoding regulatory variants including rs72856331 is often challenging. Here we have combined bioinformatics prediction and rich functional experiments to discover *GLI3*, an integral component of the Hedgehog (Hh) signaling pathway (49), as a responsible factor to mediate the regulatory effects of the GC risk allele rs72856331. We further showed that in GC cells, depletion of *GLI3* attenuated cellular proliferation rate and invasiveness while using mouse models; the role of GLI3 driving tumor growth and metastasis in vivo was also clearly observed. Strikingly, clinical data sets corroborate an evident impact of *GLI3* on the prognostic outcomes of GC patients. Given the pivotal roles of both *USP47* and *GLI3* in GC pathogenesis, it is plausible to hypothesize that the *GLI3*-rs72856331-*USP47* axis may play a substantial role in GC development. This axis represents a potential mechanistic pathway through which genetic variants in the promoter region of *USP47*, influenced by GLI3 binding, could contribute to the progression and severity of GC.

Finally, we discovered an USP47-mediated malignant phenotype in GC via the EMT pathway. In line with a previous study (35), we mechanistically solidified the EMT regulator Snai1 as a substrate of USP47. Through Co-IP and deubiquitination assays, and notably using a novel degradation tag (dTAG) system (36) for USP47 degradation, we confirmed that USP47 plays a crucial role in maintaining Snai1 protein stability. Furthermore, the G/G genotype at the rs72856331 locus is associated with the upregulation of EMT-related genes, indicative of a more invasive and metastatic phenotype in GC cells.

Snai1, as a key EMT regulator, facilitates the transition of epithelial cells into mesenchymal-like states, promoting increased cell motility, invasion, and resistance to apoptosis (50). These processes are critical for enabling tumor cells to disseminate from primary sites, invade surrounding tissues, and establish metastatic lesions. In line with its established role, our findings reveal that Snai1 overexpression partially rescues the impaired phenotypes resulting from USP47 depletion. This underscores USP47’s function as a promoter of GC malignancy, at least in part by regulating Snai1 deubiquitination within the EMT signaling pathway.

These observations in combination with an eQTL synergistic analysis (40) using a clinical dataset offer translational value for patient benefit, demonstrating that the risk genotype of rs72856331 correlates not only with increased *USP47* expression but also directly with poorer prognoses in GC, further strengthening our initial finding for an association of rs72856331/*USP47* and GC susceptibility. This highlights the importance of integrating genetic and transcriptomic markers for prognostic assessments in GC. Such integration could deepen our understanding of disease progression and facilitate the development of more precise prognostic models, ultimately leading to better-informed treatment strategies. Moreover, identifying this risk allele as a modulator of *USP47* expression and its influence on EMT signaling pathways opens avenues for personalized therapeutic interventions. Targeting USP47 or its downstream pathways represents a promising approach for patients harboring the rs72856331 risk genotype, with the potential to mitigate the aggressive progression associated with this genetic predisposition.

There are several limitations to our study. First, previous studies have suggested a connection between USP47 and Snai1 (50, 51). Building on this foundation, our work identifies the rs72856331 SNP within the *USP47* promoter that modulates GLI3 binding, enhances *USP47* expression, and drives EMT through Snai1 stabilization, thereby contributing to GC progression. This finding offers insights into GC pathogenesis and potential clinical applications for patient stratification and personalized therapies. Second, we did not investigate the prevalence of the rs72856331 SNP in *H*. *pylori*–induced GC due to the lack of *H*. *pylori* status data during patient enrollment and the infeasibility of retrospective serum analysis. Future studies incorporating *H*. *pylori* infection data are needed to explore the interplay between genetic predisposition and environmental factors. Lastly, as our study is based on a single clinical center, future association studies would benefit from multicenter validation to improve the robustness of their findings.

In conclusion, our study identifies a GC-susceptible SNP, rs72856331, located in the promoter region of *USP47* and elucidates its causal role in cancer-related phenotypes. We discovered that the TF GLI3 binds to the rs72856331-containing promoter region, enhancing USP47 expression, thereby contributing to GC pathogenesis. This research not only enhances our understanding of GC pathogenesis but also holds potential implications for GC prevention and treatment strategies.

## Methods

### Sex as a biological variable.

Male and female human GC samples and healthy controls were analyzed. Female mice were used in all mouse studies. In this study, sex was not considered as a biological variable.

### Study subjects and genotyping.

We conducted a case-control study with 1,076 gastric GC patients and 1,041 healthy controls from Eastern China to identify functional genetic variants in the USP family. Participant demographics are provided in Supplemental Table 1. GC cases were recruited from Fudan University Shanghai Cancer Center (FUSCC) between January 2009 and March 2011, with approval from the FUSCC Institutional Review Board. Controls were selected from the Taizhou Longitudinal Study (TZL) during the same period, frequency matched by sex and age (± 5 years), as previously described (52). Genomic DNA from all subjects was genotyped using Illumina Infinium Global Screening Array, as detailed in prior publications (53). Nongenotyped SNPs were imputed using IMPUTE software (version 2.3.1) with the reference panel of the 1000 Genomes Project Phase III (October 2014 release). SNPs with MAF of less than 0.01, poor imputation quality (info score < 0.5), or significant deviation from Hardy-Weinberg equilibrium (*P* < 1 × 10^–5^) were excluded.

### RNA isolation and real time RT-qPCR.

Total RNA was extracted from 1 × 10^7^ cells using TRIzol reagent (Thermo Fisher Scientific) following the manufacturer’s instructions. Complementary DNA (cDNA) synthesis was conducted using 1 μg of the total RNA with the Script II Reverse Transcription Kit (Vazyme, R323-01). Quantitative PCR (qPCR) was subsequently performed using 2× Taq Pro Universal SYBR qPCR Master Mix (Vazyme, Q712-02-AA), adhering to the manufacturer’s guidelines. The primer sequences utilized in this study are listed in Supplemental Table 6.

### Lentivirus transduction.

*USP47*-targeting shRNAs and control shRNAs were synthesized by annealing complementary DNA oligonucleotides and cloned into the pLKO.1 plasmid. Similarly, *GLI3*-targeting sgRNAs were cloned into the lentiCRISPR, version 2, vector for lentivirus production. Lentivirus packaging was performed by cotransfecting 293T cells with shRNA or sgRNA plasmids psPAX2, and pMD2.G. Viral particles were harvested 48 hours after transfection, filtered, and used to infect target cells for 24 hours with Polybrene. Infected cells were selected with puromycin (1.5 μg/ml) for stable conversion. Knockdown efficiency was validated by Western blotting and RT-qPCR.

### Culture of cell lines.

Reagents and materials used in this study are listed in Supplemental Table 5. The GT38, HGC-27, SUN719, and SNU668 cell lines were obtained from the Cell Bank of the Chinese Academy of Sciences. These cell lines were cultured in RPMI 1640 medium (Gibco, Thermo Fisher Scientific) with 10% FBS (Yeasen) and 1× penicillin-streptomycin (Gibco, Thermo Fisher Scientific). HEK293T cells were cultured in DMEM (BasalMedia) with 10% FBS and 1× penicillin-streptomycin. All cells were maintained at 37°C in 5% CO_2_ and regularly tested for mycoplasma contamination.

### Cell viability and proliferation assays.

Cells in logarithmic growth were plated in triplicate in 96-well plates and allowed to adhere overnight. Cell viability was assessed using the CCK-8 assay (Yeasen, 40203ES80), with absorbance measured at 450 nm according to the manufacturer’s protocol. For colony formation, 5 × 10³ cells per well were seeded in 6-well plates in triplicate and incubated for 2 weeks. After fixation with 4% paraformaldehyde and staining with 0.5% crystal violet, colonies were rinsed with PBS, air-dried, and photographed.

### Invasion and migration assays.

For the invasion and migration assays, 1 × 10^5^ cells in 500 μL serum-free medium were seeded into Transwell chambers, either precoated with Matrigel (for invasion) or uncoated (for migration). The lower chamber contained 750 μL medium with 20% FBS. After 1–2 days of incubation, cells that migrated or invaded through the membrane were fixed, stained with crystal violet, and counted under a microscope.

### Functional prediction for rs72856331.

The functional scores for GC risk SNPs were computed using integrated online SNP function prediction platforms, namely HaploReg, version 4.2 (https://pubs.broadinstitute.org/mammals/haploreg/haploreg.php), and RegulomeDB (https://www.regulomedb.org/regulome-search/). Both of these resources provided regulatory scores for the candidate SNPs, encompassing parameters such as changes in transcription motifs, DNase peak activity, and eQTL associations.

### Construction of reporter plasmids and Dual-luciferase report assay.

Synthetic DNA sequences flanking rs72856331, containing either the nonrisk allele A or the risk allele G, were cloned into the pGL3 luciferase reporter vector. These target plasmids, along with a Renilla control plasmid as an internal control, were transfected into cells in 24-well plates using Lipofectamine 3000 (Thermo Fisher Scientific). Cotransfections were performed with or without sgRNAs targeting GLI3. Luciferase activity was measured 24 hours after transfection using the Dual-Glo Luciferase Assay System (Promega). The resulting Firefly luciferase activity was normalized against the Renilla luciferase values to calculate the final luciferase activity.

### Single nucleotide mutation using CRISPR/Cas9.

This experiment was conducted with slight modifications to the method previously described in a publication by Wei et al. (40, 43). Briefly, a sgRNA targeting the upstream region of rs72856331 within the USP47 locus was designed using the CHOPCHOP tool (http://chopchop.cbu.uib.no) and cloned into the PX458 vector. DNA sequences around rs72856331, with either allele A or G, were PCR amplified and cloned into a donor plasmid. HGC-27 and GT38 cells (2 × 10^6^) were electroporated with 2 μg of the Precise Integration into Target Chromosome (PITCh) sgRNA plasmid and 4 μg of the donor plasmid using the Neon system. After 2 days, puromycin (1.5 μg/ml) was added for selection over 2–3 weeks. Surviving clones were transferred to 96-well plates, identified, and validated by genotyping.

### EMSA.

Single-stranded oligonucleotides centered on rs72856331 with either the G or A allele (sequences in Supplemental Table 6) were biotin labeled and annealed into double strands. These oligonucleotides were incubated with purified His-tagged Gli3/SP1 proteins in EMSA buffer (20 mM Tris-HCl, pH 7.5, 50 mM NaCl, 5 mM MgCl2, 0.2 mM EDTA, 1 mM DTT) for 1 hour at room temperature. The DNA-protein mixtures were electrophoresed on a 6% native polyacrylamide gel and scanned with the Amersham Typhoon 5 imaging system. For competition assays, unlabeled oligonucleotides were included in the reaction.

### Chromatin IP assay.

1 × 10^7^ Cells were cross-linked with 1% formaldehyde for 20 minutes, quenched with 125 mM glycine for 5 minutes, and then washed with PBS. The pellets were resuspended in lysis buffer and sonicated to obtain chromatin fragments (200–700 bp). The lysate was centrifuged to collect sheared chromatin. This was incubated overnight with H3K27ac antibody (abclonal, A7253) at 4°C, followed by incubation with magnetic protein A/G beads for 3 hours. Chromatin was washed with high- and low-salt buffers, then eluted and crosslinks reversed in elution buffer with protease K at 65°C overnight. DNA was extracted and analyzed by qPCR or prepared for sequencing using the VAHTS Universal Plus DNA Library Prep Kit for Illumina (Vazyme). The library was sequenced on the NovaSeq 6000 platform (Annoroad Gene Technology).

### CUT&Tag.

CUT&Tag was performed with modifications from a previously described method (54). Nuclei (1–2 × 10^6^) were isolated using NE1 buffer and conjugated to ConA magpoly beads (Smart-Lifesciences, SM04102). After overnight incubation at 4°C with primary antibodies (1:50 dilution in primary antibody buffer), secondary antibodies were added for 1 hour at room temperature in dig-wash buffer. pAG-Tn5 was introduced in dig-300 buffer (1:50) and incubated for 1 hour, followed by tagmentation in dig-300 buffer with 10 mM Mg². DNA was purified with VAHTS DNA clean beads (VAHTS, N411) and amplified using the TruePrep DNA Library Prep Kit V2 for Illumina (VAHTS, TD501). Libraries were pooled and sequenced on a NovaSeq (Illumina) platform with 150 bp paired-end reads. Primary antibodies included anti-H3K27ac (Abclonal, A7253), with goat anti-rabbit IgG (Novoprotein, N269) as the secondary antibody.

Raw CUT&Tag reads were trimmed with Trim Galore, version 0.6.6, aligned to the human hg19 genome using Bowtie, version 2.4.4, and duplicate reads removed with Picard Tools, version 2.25.5. Reads were shifted for tagmentation site offsets using deepTools, version 3.5.1’s alignmentSieve function with the ‘--ATACshift’ option.

### Gene expression correlation analysis.

Pearson’s product-moment correlation and Spearman’s rank correlation rho analyses were conducted to assess the expression correlation between USP47 and key TFs including GLI3, ETS1, SP1, ETV7, and SP3. A significance threshold was set at *P* < 0.05. These correlations were calculated using data from multiple independent GC cohorts, providing a comprehensive analysis of their potential coexpression relationships.

### His-tag protein purification.

The His-tagged GLI3/SP1 plasmid was transformed into BL21(DE3) pLysS *E*. *coli* cells (TIANGEN, CB106). A single colony was cultured overnight in Luria-Bertani (LB) medium with ampicillin, then expanded in 1 L of LB medium with 100 μg/mL ampicillin. When the OD600 reached 0.6, 0.25 mM isopropyl β-D-1-thiogalactopyranoside (IPTG) was added to induce protein expression for 5 hours at 23°C. The cells were harvested, resuspended in resuspending buffer with protease inhibitor, and lysed by high-pressure homogenization. The lysate was centrifuged to collect the supernatant, and optional polyethylenimine (PEI) (Sigma-Aldrich, P3143) was added to remove bacterial DNA. His-tagged protein was purified using Ni-NTA beads 6FF (Smart-Lifesciences, SA004005) and eluted with 250 mM imidazole. The protein was dialyzed against dialysis buffer and stored with glycerol.

### Nude mice tumorigenicity and metastasis assay.

Four-week-old female BALB/C nude mice, acquired from Shanghai Model Organisms, were maintained under specific pathogen–free (SPF) conditions at the Fudan University Shanghai Cancer Center’s animal care facility. Mice were randomly assigned to 3 groups and injected with 1 × 10^6^ HCG-27 and GT38 cells with different treatments in the right flank. Tumor growth was monitored starting 7 days after injection, with measurements taken twice weekly. Tumor volume was calculated using (width^2^ × length)/2.

For metastasis assays, treated cells were injected into the tail veins of NOD/SCID mice (purchased from Shanghai Model Organisms), and bioluminescence imaging was performed and analyzed 6 weeks after injection.

### RNA-Seq and data analysis.

RNA was extracted from *USP47*-knockdown and control HGC-27 cells in triplicate using established protocols. mRNA was captured with VAHTS mRNA Capture Beads (Vazyme), and RNA-Seq libraries were prepared using the VAHTS Universal V6 RNA-Seq Library Prep Kit for Illumina (Vazyme). Following quality assessment with an Agilent 2100 Bioanalyzer, the library was sequenced on an Illumina NovaSeq 6000 platform (Annoroad Gene Technology).

RNA-Seq data were processed with a modified pipeline (55). FastQC was used for quality control, trimming nucleotides with a sequencing quality score below 20. Reads were mapped to the human hg19 genome using STAR, version 2.7.5c, with duplicates and low-quality reads removed via SAMtools, version 1.9. Differential gene expression was analyzed with DESeq2 (1.26.0), and gene set enrichment analysis (GSEA) was performed on hallmark gene sets.

### Co-IP.

Approximately 1 × 10^7^ HGC-27 cells were resuspended in lysis buffer (20 mM Tris-HCl, pH 8.0, 150 mM NaCl, 1 mM EDTA, 0.5% NP40, 10% glycerol, with protease inhibitor) and incubated for 30–60 minutes for lysis. The supernatant was collected after centrifugation and incubated with 2–5 μg of specific antibody (IgG as a control) for 2–4 hours at 4°C. Protein A/G magnetic beads, preblocked with BSA, were added and incubated for 1 hour at 4°C. After washing with lysis buffer, the samples were eluted with SDS loading buffer for Western blot analysis.

### In vitro ubiquitination assays.

HGC-27 cells were treated with the proteasome inhibitor MG132 (MCE, HY-13259) for 6 hours, lysed in buffer for 30 minutes, and subjected to overnight IP at 4°C with an anti-Snail antibody (CST, 3879) using IgG antibody (Proteintech) as a control. Ubiquitination levels were assessed by Western blot with an antiubiquitin antibody (CST, 3936S).

### Rapid degradation system for UPS47.

The *USP47*-dTAG cell line was generated with modifications to a previous method (36). HGC-27 cells (1 × 10^6^) were electroporated with PITCh plasmids using the Neon system, recovered in antibiotic-free medium for 2 days, and selected with 1 μg/ml puromycin (Meilunbio) for 2 weeks. Single clones were isolated, genotyped by PCR, and analyzed by Western blot after dTAG13 treatment for 3 hours. The clone with the highest USP47 degradation efficiency was selected for further experiments.

### Western blot assays.

Total protein was extracted from 2 × 10^6^ cells cultured in 6-well plates using lysis buffer, denatured with SDS-loading buffer at 95°C for 10 minutes, and separated by SDS-PAGE. Proteins were transferred to a nitrocellulose membrane and blocked with QuickBlock Western buffer (PS108). Primary antibodies (listed in Supplemental Table 5) were incubated overnight, followed by washing with TBST and incubation with secondary antibody (1:5000, AS014 and AS003) for 1 hour. Immunoblots were visualized using a chemiluminescence kit (EpiZyme SQ101) and quantified with ImageJ (ImageJ 1.5, NIH).

### Statistics.

We performed association analyses using PLINK software (version 1.90) for 1,273 SNPs of 23 USPs and their 2 kb upstream and downstream regions. Logistic regression was used to assess the association of each SNP with GC susceptibility. This analysis was executed under an additive genetic model, with adjustments made for potential confounding variables, specifically age and sex. Multiple-test correction was conducted using the BFDP methods, with a prior false-positive probability set at 0.1 to minimize false positives. Associations yielding *P* values of less than 0.05 and BFDP of less than 0.8 were considered as significant findings with acceptable false-positive probability.

Linear regression analyses were employed to assess eQTL effects. Kaplan-Meier survival analysis was performed to generate survival curves, and the log-rank test was used to calculate *P* values and hazard ratios (HRs) to determine the relationship between gene expression levels of rs72856331 genotypes and 5-year survival rates or progression-free probabilities in GC patients. Statistical tests for investigating the correlation between the expression levels of *USP47* or *GLI3* and clinical features, including tumor stages, lymph node stages, and grades were evaluated by the Kruskal-Wallis H test and Dunn’s post hoc tests for pairwise group comparisons. Bar charts represent means ± SDs, with a 2-tailed Student’s *t* test applied for comparisons between 2 independent groups. For multiple gene comparisons and multiple group comparisons, 1-way ANOVA or 2-way ANOVA was performed, followed by the Holm-Šidák multiple-comparison test for multiple comparisons. All experiments were conducted with 3 independent biological replicates. All statistical tests were 2-sided, and a *P* value of less than 0.05 was considered statistically significant. Statistical tests were performed using RStudio (v. 1.4.1106) with R version, version 4.1.0.

### Study approval.

All eligible participants in the present study completed a written, informed consent form. The collection of human gastric tissues and DNA samples was approved by the Institutional Review Board of Fudan University Shanghai Cancer Center. All procedures followed the National Institutes of Health’s Guide for the Care and Use of Laboratory Animals and were approved by the Fudan University Shanghai Cancer Center Ethical Committee.

### Data availability.

Publicly available data can be found in the TCGA and GEO databases. The RNA-Seq and CUT&Tag sequencing data have been deposited in the NCBI’s Gene Expression Omnibus (GEO GSE286483 and GSE286484). Our dataset used in the current study is provided in the Supporting Data Values file or is available from the corresponding authors upon reasonable request.

## Author contributions

MW and FXC designed the research studies with help from GHW and TX. BT, Zhenning Wang, and XW and conducted experiments with help from GHW and TX. BT, Zhenning Wang, and XW conducted experiments with help from JL, JW, ZC, and PZ. AS acquired and analyzed data with help from QZ, Zixian Wang, and WX. MS and YW helped with patient sample collection and processing. MW, FXC, GHW, and BT wrote the manuscript. The order of the first authors was determined by assessing the relative extent of their experimental contributions, with BT making the most substantial contributions, followed by Zhenning Wang and then XW.

## Supplementary Material

Supplemental data

Unedited blot and gel images

Supplemental tables 1-6

Supporting data values

## Version 1 02/25/2025

In-Press Preview

## Version 2 04/15/2025

Electronic publication

## Version 3 09/29/2026

Corrected supplemental figure 7J and associated supporting data values.
