# Corrigendum to An inherited predisposition allele promotes gastric cancer via enhancing deubiquitination-mediated activation of epithelial-to-mesenchymal transition signaling

**DOI:** 10.1172/JCI212379

**Published:** 2026-10-01

**Authors:** Bolin Tao, Zhenning Wang, Xuanyi Wang, Aixia Song, Jiaxian Liu, Jianan Wang, Qin Zhang, Zhaolin Chen, Zixian Wang, Wenjie Xu, Menghong Sun, Yanong Wang, Ping Zhang, Tao Xu, Gong-Hong Wei, Fei Xavier Chen, Mengyun Wang

Original citation: *J Clin Invest.* 2025;135(8):e179617. https://doi.org/10.1172/JCI179617

Citation for this corrigendum: *J Clin Invest*. 2026;136(19):e212379. https://doi.org/10.1172/JCI212379

During a recent reexamination of their original datasets, the authors identified the following error in Supplemental Figure 7J: tumor-volume values were duplicated in columns 2 and 4 for days 9 through 19. The tumor growth curves have been generated from the original measurements using the formula V = l × w^2^/2 and the statistical analyses repeated.

The HTML and PDF versions, including the Supplemental material and Supporting data values, have been updated online.

The authors regret the error.

## Version 1 10/01/2026

Electronic publication

