# Supplementary material for: An inherited predisposition allele promotes gastric cancer via enhancing deubiquitination-mediated activation of epithelial-to-mesenchymal transition signaling
Source: J Clin Invest. 2025 Feb 25;135(8):e179617. doi: 10.1172/JCI179617 (PMC11996917; doi:10.1172/JCI179617)
Supplement: Supplemental data [file jci-135-179617-s068.pdf]

# Tao.et al.Figure S1

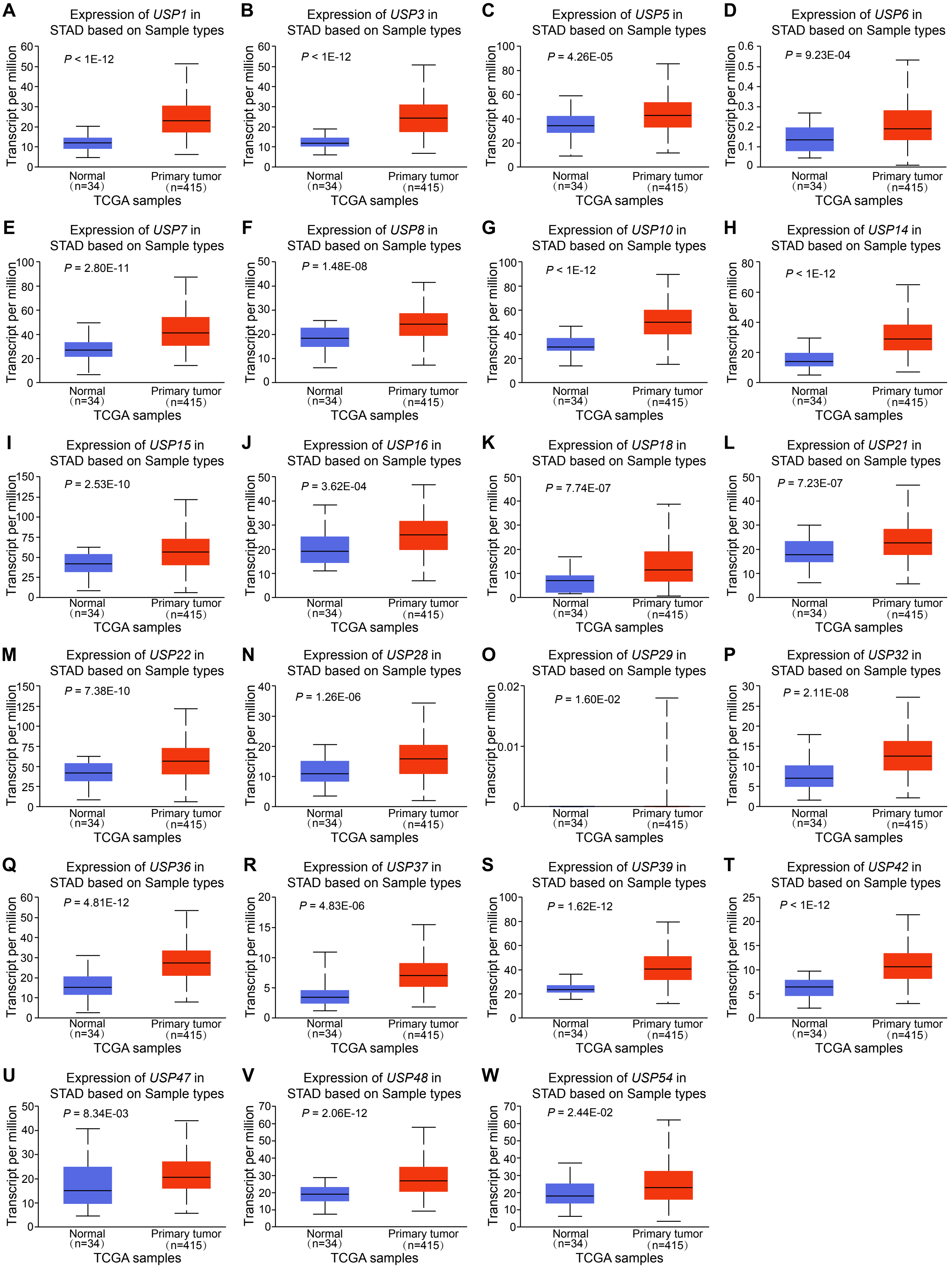

**Supplemental Figure 1. 23 USPs characterized as oncogenes with elevated mRNA expression in GC tissues relative to normal gastric counterparts within the GEPIA dataset.**

**A-W**. The GEPIA dataset showed significantly elevated expression in GC tissues of 23 USPs characterized as oncogenes, in gastric cancer tissues compared to normal tissues. Data represented as means ± SD, statistically assessed by a two-tailed t-test.

**Tao.et al.Figure S2**

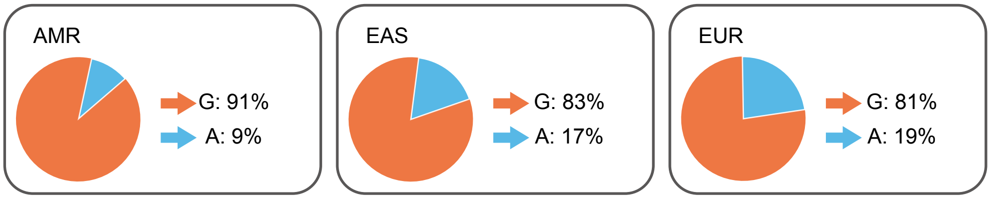

**Supplemental Figure 2. The minor allele frequency (MAF) of rs72856331 across different populations.**

AMR: American population; EAS: East Asian population; EUR: European population.

# Tao.et al.Figure S3

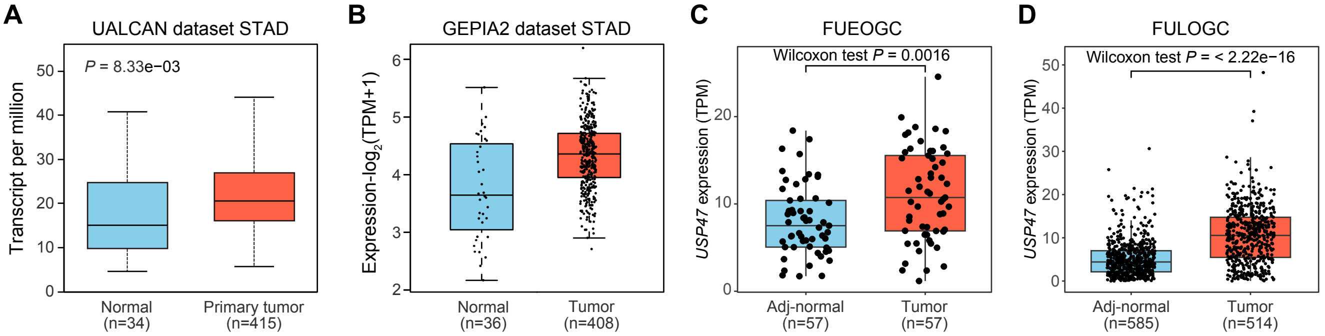

**Supplemental Figure 3. Comparative analysis of *USP47* expression in gastric cancer tissues versus normal tissues across multiple datasets.**

**A**. *USP47* expression data from the UALCAN database.

**B**. *USP47* expression data from the GEPIA2 database.

**C** and **D**. *USP47* expression data from our in-house cohorts of early-onset (FUEOGC) and late-onset (FULOGC) gastric cancer. Statistical significance was assessed using the Wilcoxon test, with $P < 0.05$ considered significant.

# Tao.et al.Figure S4

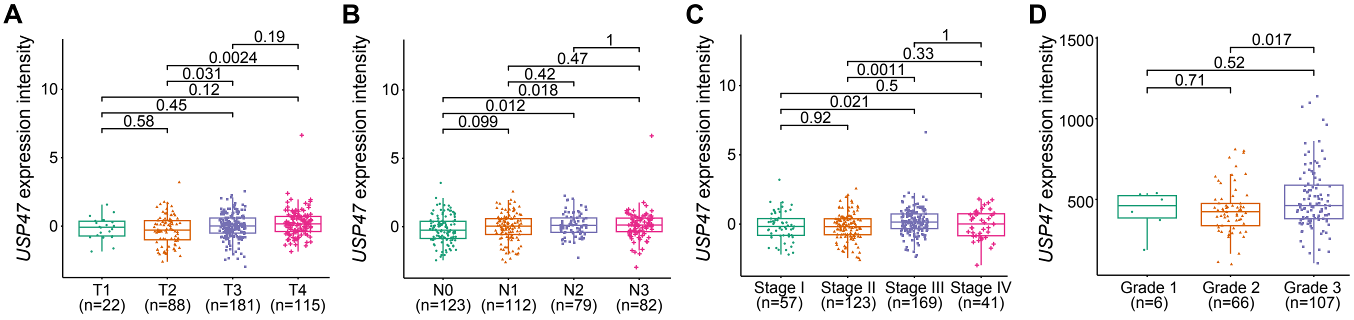

**Supplemental Figure 4. Pairwise comparisons of *USP47* expression levels across clinical status of gastric cancer in the TCGA STAD cohort.**

**A**-**D**: Pairwise comparisons of *USP47* expression levels in relation to GC clinical features, including (**A**) tumor invasiveness (n=406); (**B**) lymph node metastasis (n=394); (**C**) tumor stages (n=390), and (**D**) tumor grade (n=179). Statistical significance was determined by Dunn's post-hoc tests, with a significance threshold set at $P < 0.05$.

# Tao.et al.Figure S5

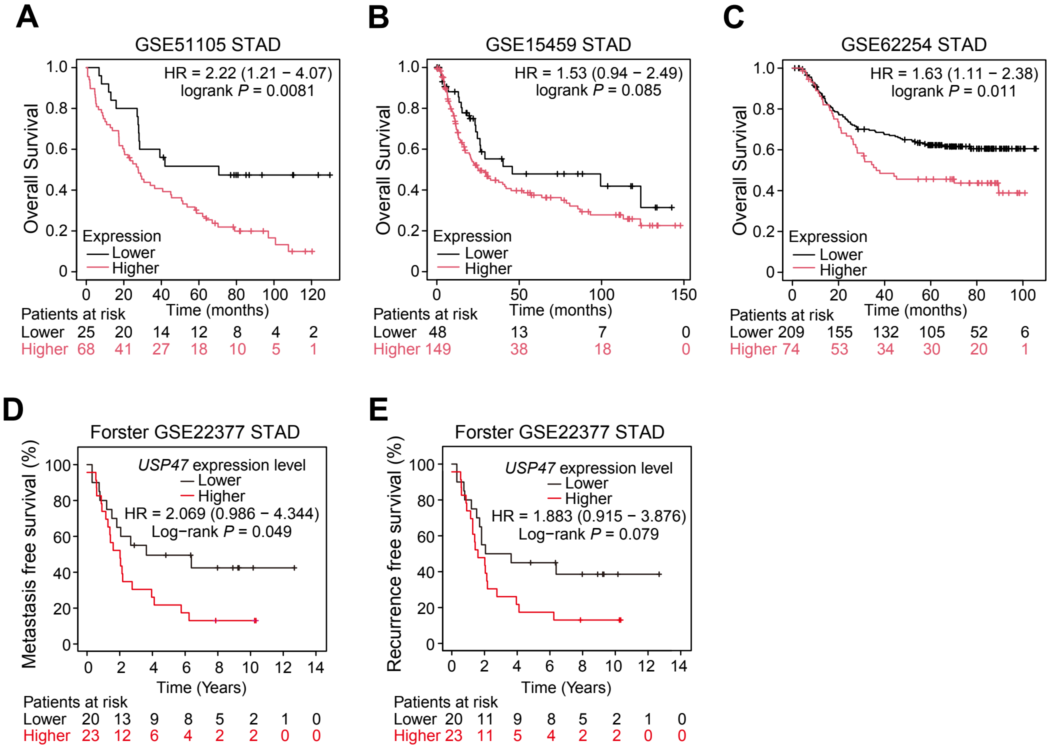

**Supplemental Figure 5. Elevated *USP47* expression is associated with poorer survival outcomes across multiple gastric cancer cohorts.**

**A**-**C**: Kaplan-Meier survival curves demonstrate that higher *USP47* expression is significantly associated with poorer overall survival in the GSE51105 (**A**), GSE15459 (**B**), and GSE62254 (**C**) gastric cancer cohorts.

**D** and **E**. Elevated *USP47* expression is linked to reduced metastasis-free survival (**D**) and recurrence-free survival (**E**) in the GSE22377 cohort. *P*-values were calculated using the log-rank test.

# Tao.et al.Figure S6

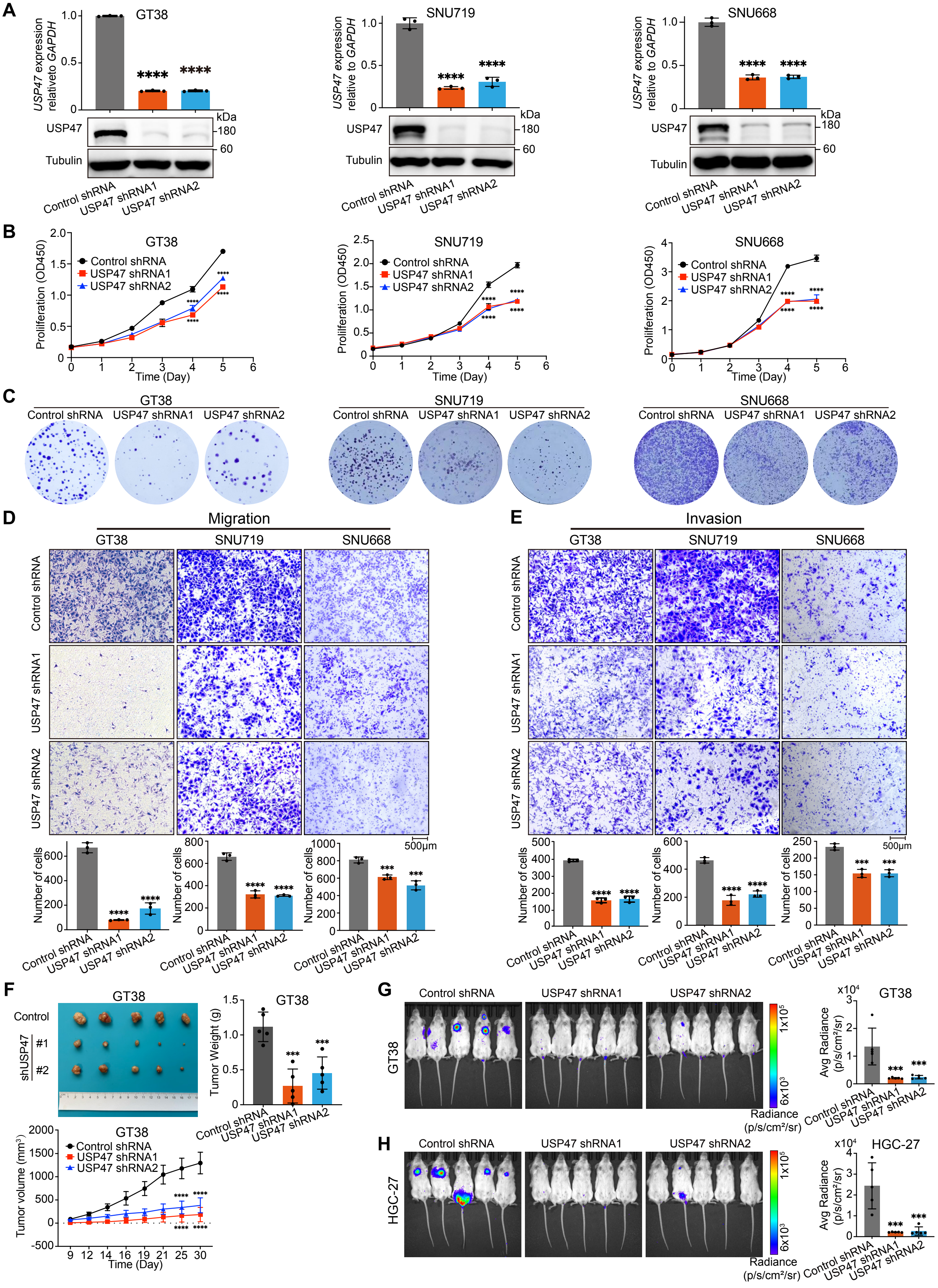

**Supplemental Figure 6. Impact of *USP47* knockdown on gastric cancer growth, metastasis, and tumorigenesis.**

**A**. Knockdown efficiency of *USP47* in GT38, SNU719, and SNU668 cell lines validated by RT-qPCR and Western blotting using two independent *USP47*-specific shRNAs (n=3).

**B**-**E**. *USP47* knockdown suppresses gastric cancer cell phenotypes, as demonstrated by CCK-8 proliferation assays (**B**), colony formation assays (**C**), migration (**D**) and invasion assays (**E**) in GT38, SNU719, and SNU668 cells under different treatments (n=3). Scale bars: 500 μm.

**F**. Representative images, growth curves, and tumor weights of xenograft tumors derived from nude mice injected with GT38 cells with *USP47* knockdown or control cells, assessed after a 4-week experimental period (n=5 per group). Significance assessed by 2-way ANOVA and Holm-Šidák post hoc test for growth curves and 1-way ANOVA with Holm-Šidák multiple comparison test for tumor weights.

**G** and **H**: *USP47* knockdown suppresses in vivo metastatic potential in gastric cancer cells. Bioluminescence images were captured and quantitatively analyzed 6 weeks after tail vein injection of GT38 cells (**G**) and HGC-27 cells (**H**) transfected with either control shRNA or *USP47*-specific shRNAs into NOD/SCID mice (n=5 per group).

Data are presented as mean ± SD. Statistical significance was calculated using 2-way ANOVA and Holm-Šidák post hoc test for panel **B** or 1-way ANOVA with Holm-Šidák multiple comparison test for panels **D, E, G** and **H.** $^{**}P < 0.01$, $^{***}P < 0.001$, $^{****}P < 0.0001$.

# Tao.et al.Figure S7

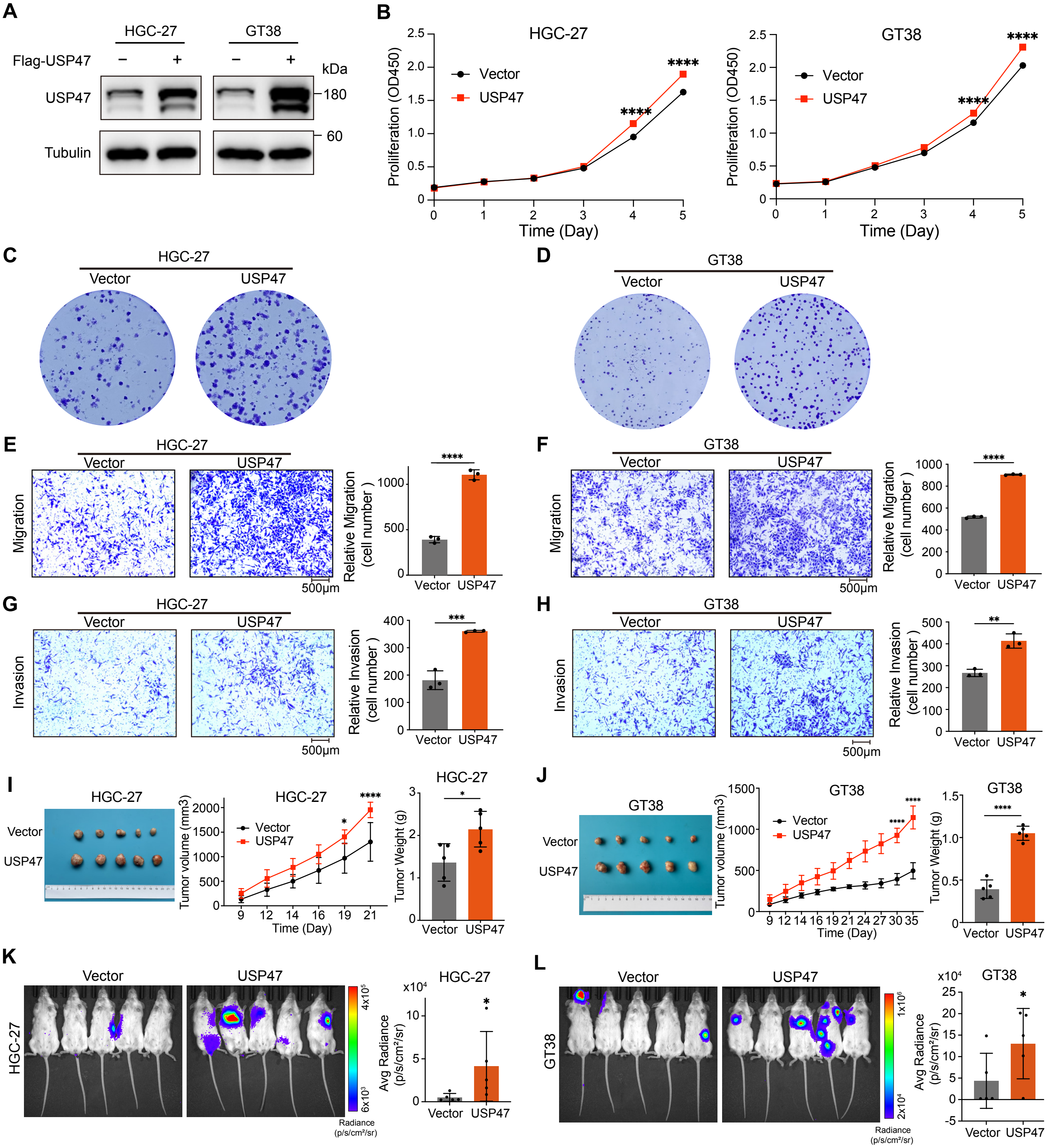

**Supplemental Figure 7.** ***USP47*** **overexpression promotes gastric cancer growth, metastasis, and tumorigenesis.**

**A**. Western blot analysis validating *USP47* overexpression in HGC-27 and GT38 cells.

**B**-**H**. *USP47* overexpression enhances gastric cancer cell phenotypes, including proliferation (**B**), colony formation (**C** and **D**), migration (**E** and **F**), and invasion (**G** and **H**) in HGC-27 and GT38 cells (n=3). Scale bars: 500 μm.

**I** and **J**. *USP47* overexpression significantly accelerates tumor growth in xenograft models using HGC-27 (**I**) and GT38 (**J**) cells, as shown by tumor volume measurements over time (n=5 per group). Significance assessed by 2-way ANOVA for growth curves and a two-tailed student's t-test for tumor weights.

**K** and **L**. Bioluminescence imaging and quantitative analysis of 6 weeks post tail vein injection of *USP47*-overexpressing HGC-27 (**K**) and GT38 (**L**) cells into NOD/SCID mice demonstrate enhanced metastatic potential (n=5 per group).

Data are presented as mean ± SD. Statistical significance was calculated using a two-tailed Student's t-test for panels **E**, **F**, **G**, **H**, **K** and **L**, or two-way ANOVA for panel **B**: *$P < 0.05$, **$P < 0.01$, ***$P < 0.001$, ****$P < 0.0001$.

**Tao.et al.Figure S8**

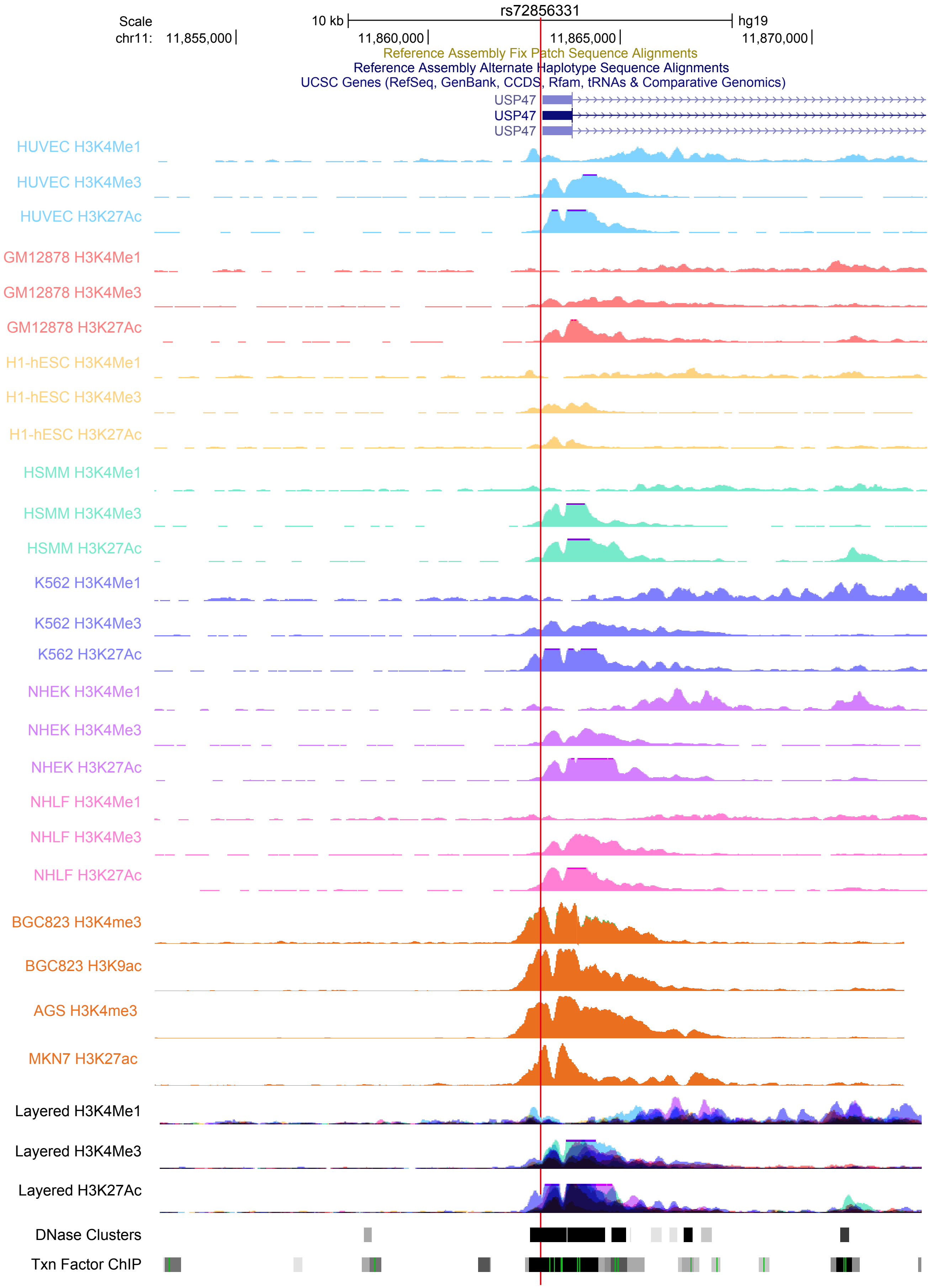

**Supplemental Figure 8. The 72856331-containing region is a potential transcriptional regulatory element across gastric cancer cell lines.**

The diagram illustrates the distribution of histone modifications (H3K4me1, H3K27ac, H3K4me3, H3K9ac), transcription factor binding sites, and DNase I hypersensitive sites surrounding rs72856331 (highlighted by a red line) in multiple gastric cancer cell lines, as sourced from the UCSC Genome Browser dataset.

# Tao.et al.Figure S9

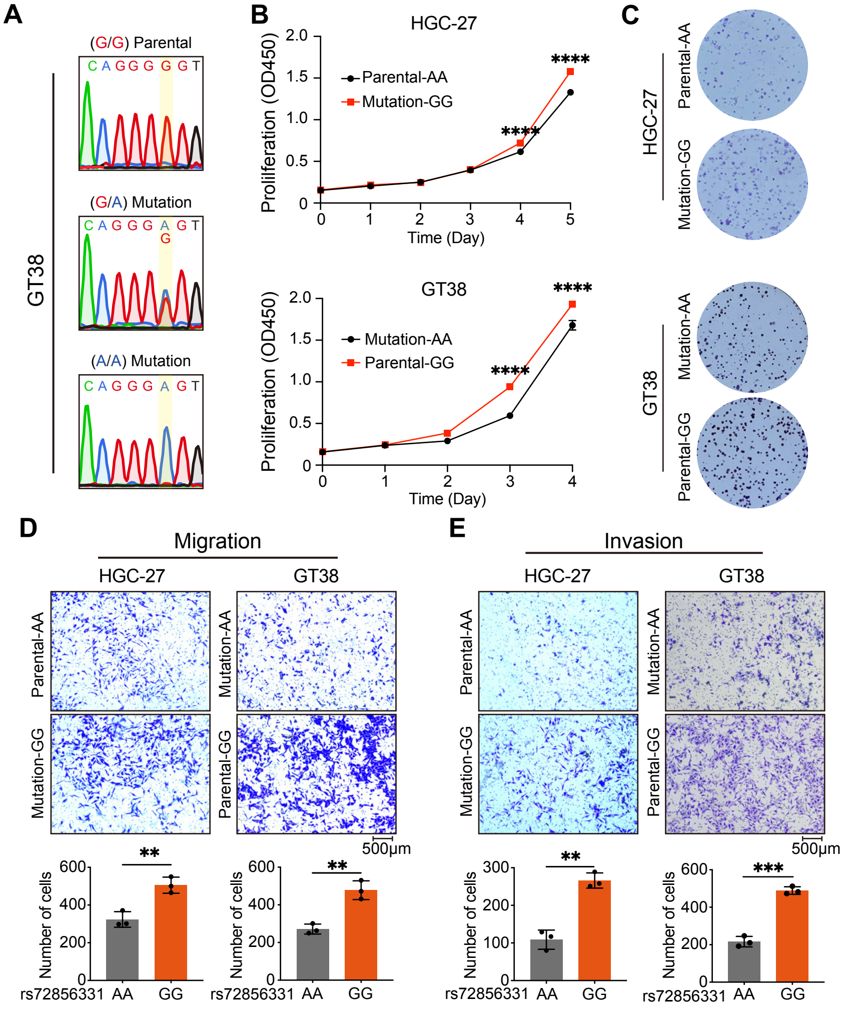

**Supplemental Figure 9. The impact of rs72856331 genotype on gastric cancer cell growth, migration, and invasion.**

**A**. Sanger sequencing confirming CRISPR/Cas9-modified genotypes in GT38 cells compared to parental controls.

**B**. Proliferation analysis of CRISPR/Cas9-modified HGC-27 and GT38 cells compared to parental cells (n=3).

**C**. Representative images of colony formation assays for HGC-27 and GT38 cells with different rs72856331 genotypes.

**D** and **E**. Migration (**D**) and invasion (**E**) assays for HGC-27 and GT38 cells with varying genotypes (n=3). Scale bars = 500 μm.

Data are presented as mean ± SD. Statistical significance was determined using two-tailed Student's t-test for panels **D** and **E**, or two-way ANOVA for panel **B**: $^{**}P < 0.01$, $^{***}P < 0.001$, $^{****}P < 0.0001$.

# Tao.et al.Figure S10

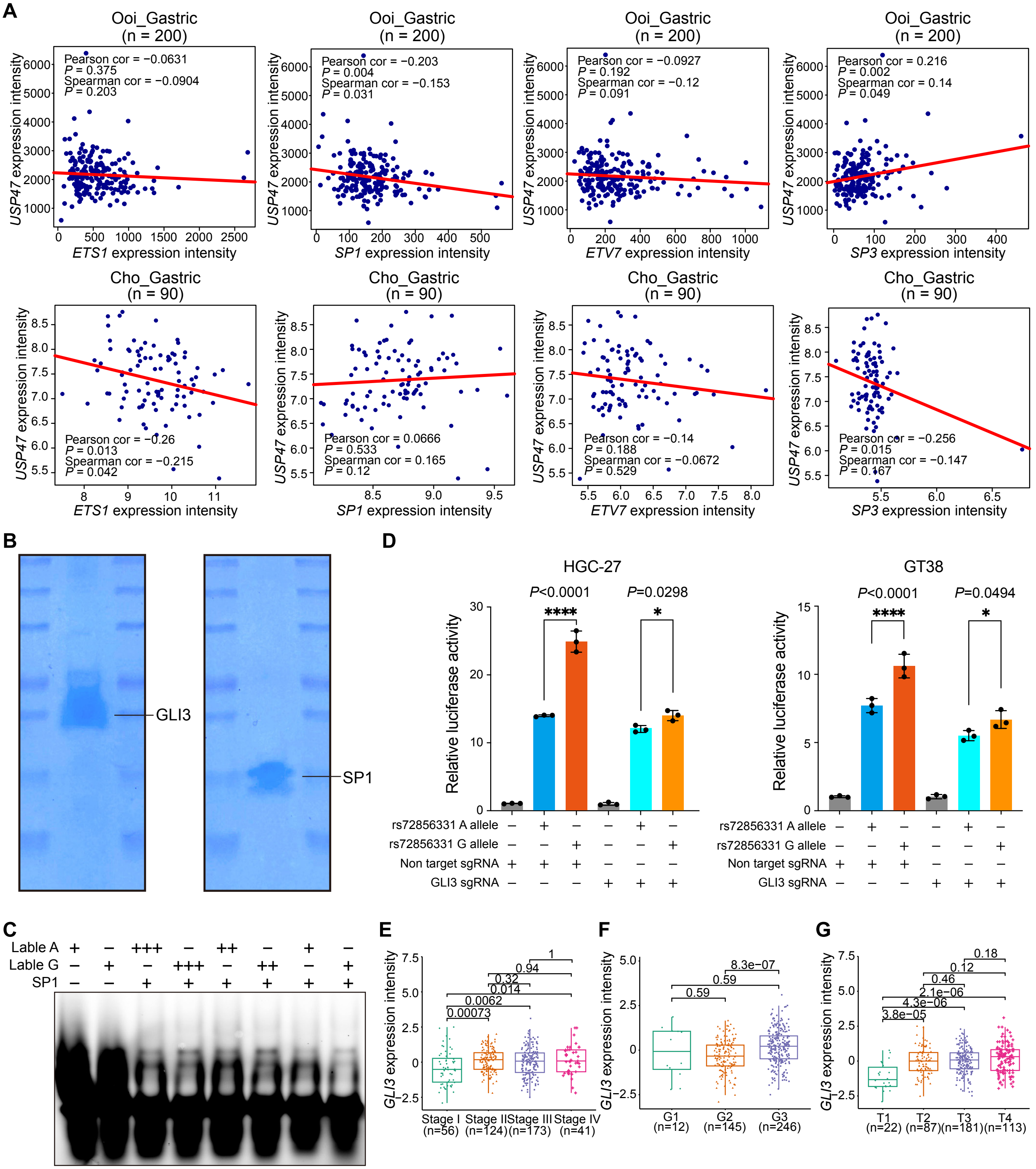

**Supplemental Figure 10. Four transcription factors predicted by the Enhancer Element Locator (EEL) algorithm showed diminished potential to regulate *USP47* expression.**

**A**. Scatterplots showing an expression correlation between four predicted transcription factors and *USP47* in the two independent GC cohorts. *P* values assessed by the Pearson correlation test.

**B**. Coomassie brilliant blue staining confirming the purity of his-tagged GLI3 and SP1 proteins used for subsequent experiments.

**C**. Electrophoretic mobility shift assay (EMSA) using purified SP1 protein demonstrates no differential binding affinity between the rs72856331 G allele and the A allele.

**D**. Relative luciferase activity in HGC-27 and GT38 cells transfected with reporter plasmids containing either the G or A allele of rs72856331, with or without GLI3-targeting sgRNAs (n=3). A statistical significance was calculated using 1-way ANOVA with Holm-Šidák multiple comparison test. $*P < 0.05$, $****P < 0.0001$.

**E-G**. Pairwise comparisons of GLI3 expression levels with GC clinical features in the TCGA STAD cohort: (**E**) tumor stages (n=394), (**F**) tumor grade (n=403), and (**G**) tumor invasiveness (n=403). Statistical significance was determined by Dunn's post-hoc tests, with a significance threshold set at $P < 0.05$.

# Tao.et al.Figure S11

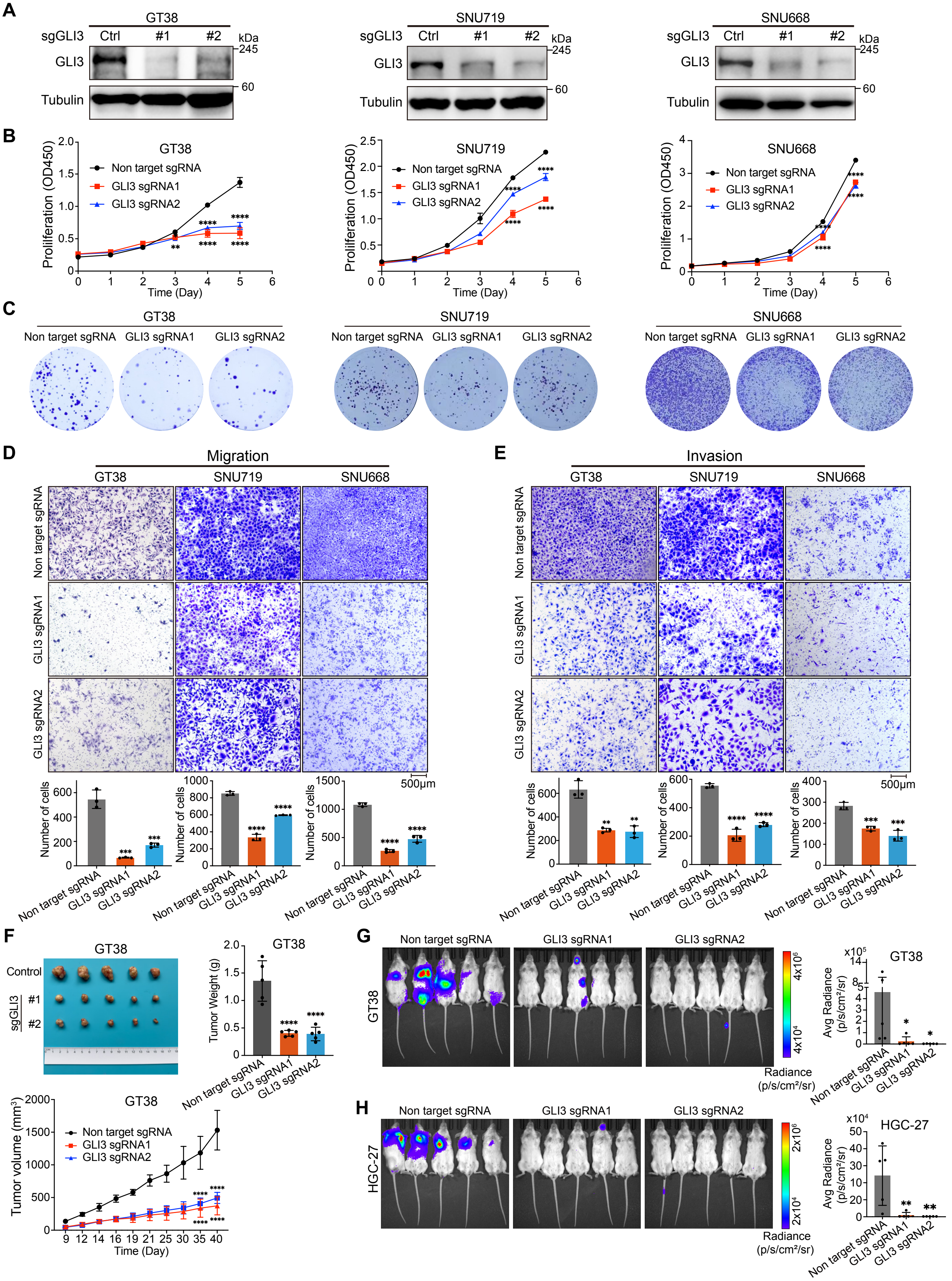

**Supplemental Figure 11. Impact of GLI3 knockout on gastric cancer growth, metastasis, and tumorigenesis.**

**A**. Knockout efficiency of GLI3 in GT38, SNU719, and SNU668 cell lines was validated by Western blotting using two independent GLI3-specific sgRNAs (n=3).

**B**-**E**. GLI3 knockout suppresses gastric cancer cell phenotypes, as demonstrated by CCK-8 proliferation assays (**B**), colony formation assays (**C**), migration (**D**) and invasion assays (**E**) in GT38, SNU719, and SNU668 cells under different treatments (n=3). Scale bars: 500 μm.

**F**. Representative images, growth curves, and tumor weights of xenograft tumors derived from nude mice injected with GLI3-knockout GT38 cells or control cells, assessed after a 6-week experimental period (n=5 per group). Significance assessed by 2-way ANOVA and Holm-Šidák post hoc test for growth curves and 1-way ANOVA with Holm-Šidák multiple comparison test for tumor weights.

**G** and **H**. Bioluminescence images were captured and quantitatively analyzed 6 weeks after tail vein injection of GT38 cells (**G**) and HGC-27 cells (**H**), transfected with control or GLI3-specific sgRNAs, into NOD/SCID mice.

Data are presented as mean ± SD. Statistical significance was calculated using 2-way ANOVA and Holm-Šidák post hoc test for panel **B,** or 1-way ANOVA with Holm-Šidák multiple comparison test for panels **D, E, G** and **H.** $^{*}P < 0.05$, $^{**}P < 0.01$, $^{***}P < 0.001$, $^{****}P < 0.0001$.

# Tao.et al.Figure S12

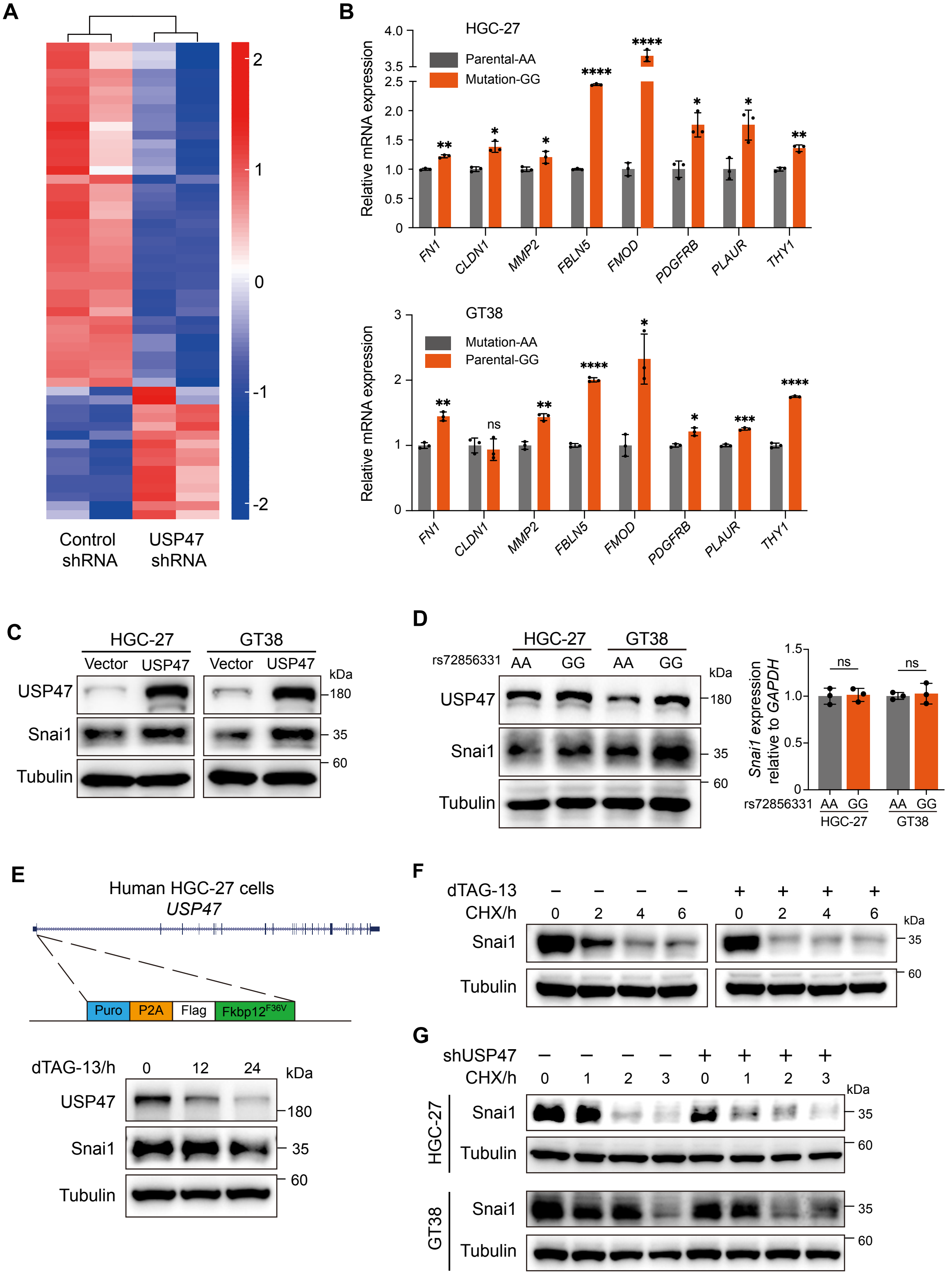

**Supplemental Figure 12. USP47 regulation of Snai1 stability and its impact on EMT pathways and gene expression in gastric cancer**

**A**. Heatmap visualization of RNA sequencing results from HGC-27 cells transduced with control shRNA compared to cells transduced with shRNAs targeting *USP47*.

**B**. RT-qPCR analysis of EMT-related genes in HGC-27 and GT38 cells with different genotypes of rs72856331 (n=3).

**C**. Western blot analysis of USP47 and Snai1 protein levels in HGC-27 and GT38 cells with USP47 overexpression.

**D**. Western blot and RT-qPCR analysis of USP47 and Snai1 expression in HGC-27 and GT38 cells with different rs72856331 genotypes at the rs72856331 locus (n=3).

**E**. Schematic representation of the dTAG system targeting USP47. Treatment with dTAG-13 for 12 and 24 hours results in significant degradation of USP47, leading to a marked decrease in Snai1 protein levels.

**F**. Western blots showing Snai1 protein levels in HGC-27 cells treated with cycloheximide (CHX), with or without USP47 knockdown.

**G**. Western blot analysis showing the effect of *USP47* knockdown on Snai1 protein stability in HGC-27 and GT38 cell lines following treatment with cycloheximide (CHX).

Data are presented as mean ± SD. Statistical significance was calculated using a two-tailed Student's t-test. *$P < 0.05$, **$P<0.01$, ***$P < 0.001$, ****$P < 0.0001$.

# Tao.et al.Figure S13

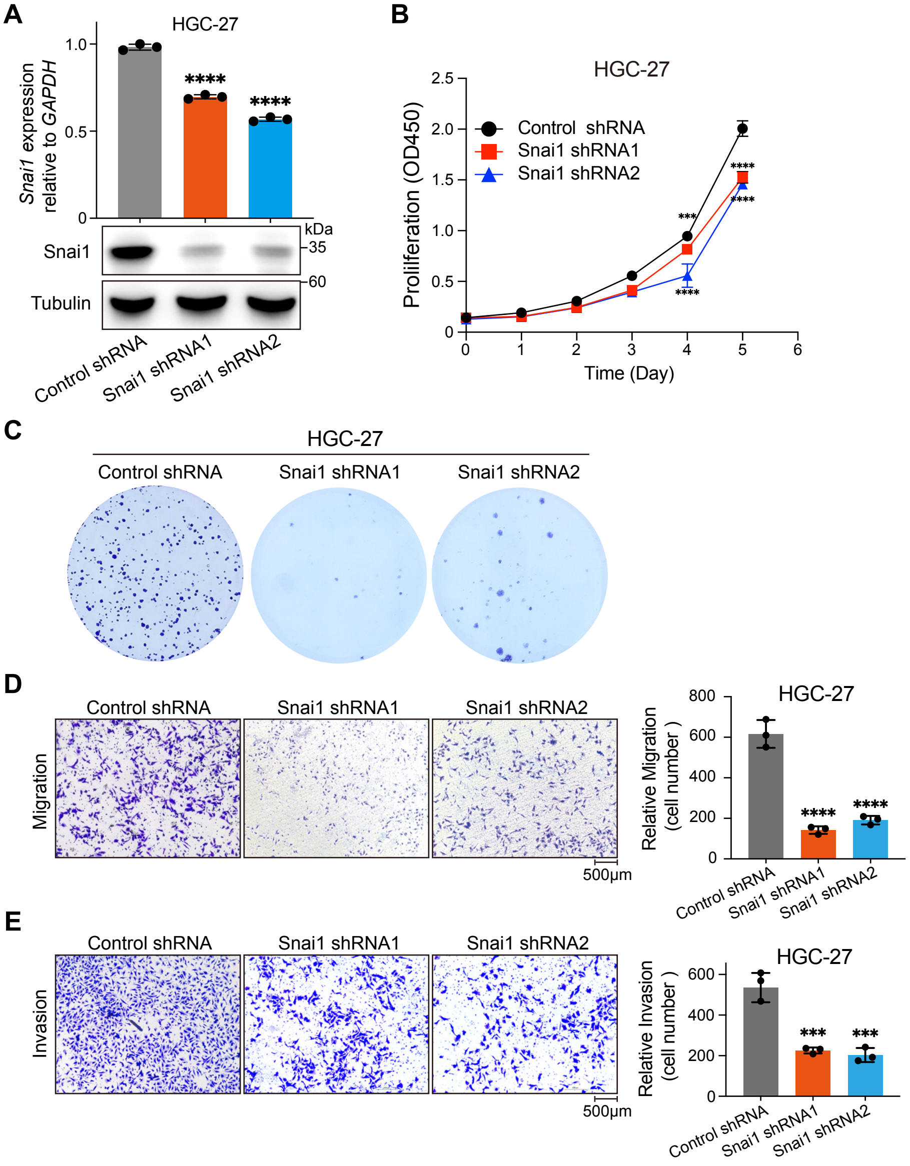

**Supplemental Figure 13. Downregulation of Snai1 reduces malignant phenotypes in HGC-27 gastric cancer cells.**

**A**. Quantitative PCR and Western blot analyses showing Snai1 mRNA and protein levels in HGC-27 cells after knockdown with two distinct Snai1-specific shRNAs.

**B**. Knockdown of Snai1 suppresses gastric cancer cell growth, as measured by the CCK-8 assay in HGC-27 cells transduced with control shRNA or Snai1-targeted shRNAs (n=3).

**C**-**E**. Representative images of colony formation (**C**), migration (**D**), and invasion (**E**) assays in HGC-27 cells transduced with control or Snai1-targeted shRNAs (n=3). Scale bars = 500 μm.

Data are presented as mean ± SD. Statistical significance was calculated using 2-way ANOVA and Holm-Šidák post hoc test for panel **B** or 1-way ANOVA with Holm-Šidák multiple comparison test for panels **D** and **E.** ***$P < 0.001$, ****$P < 0.0001$.

# Tao.et al.Figure S14

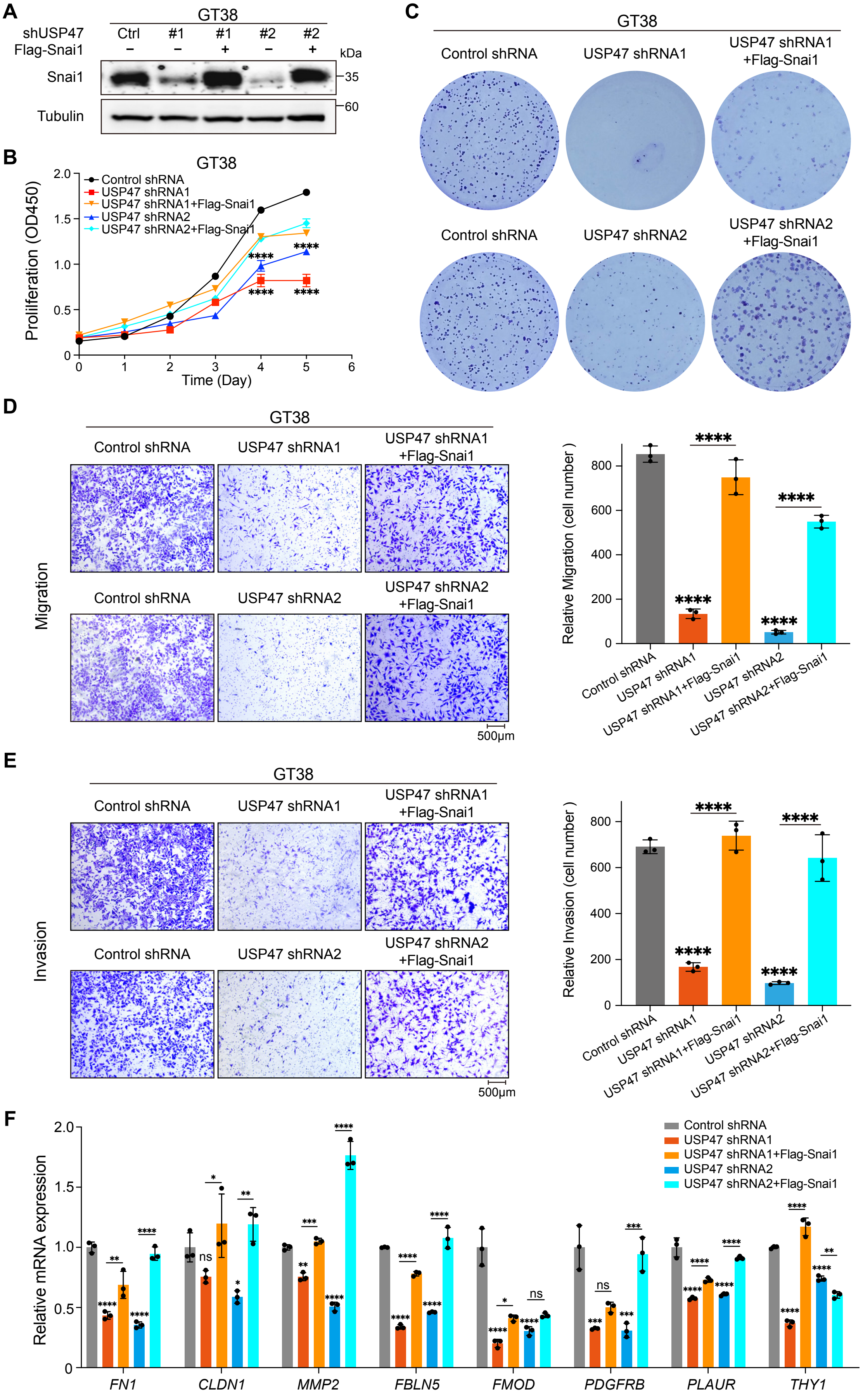

**Supplemental Figure 14. USP47 promotes gastric cancer cell proliferation, migration, and invasion by regulating Snai1.**

**A**. Western blot analysis confirming the knockdown efficiency of *USP47* using two independent shRNAs and the overexpression of Snai1 following co-transfection with Flag-Snai1.

**B**-**E**. Functional assays evaluating the effect of Snai1 overexpression on cancer cell phenotypes after USP47 depletion in GT38 cells: cell proliferation (**B**), colony formation (**C**), migration (**D**), and invasion (**E**) (n=3). Representative images are shown for the colony formation, migration, and invasion assays. Scale bars = 500 μm.

(**F**). Relative mRNA expression of EMT-related genes in *USP47*-knockdown gastric cancer cells with or without Snai1 overexpression (n=3).

Data are presented as mean ± SD. Statistical significance was calculated using 2-way ANOVA and Holm-Šidák post hoc test for panel **B** or 1-way ANOVA with Holm-Šidák multiple comparison test for panels **D, E** and **F.** *$P < 0.05$, **$P<0.01$, ***$P < 0.001$, ****$P < 0.0001$.
