# Supplementary material for: An inherited predisposition allele promotes gastric cancer via enhancing deubiquitination-mediated activation of epithelial-to-mesenchymal transition signaling
Source: J Clin Invest. 2025 Feb 25;135(8):e179617. doi: 10.1172/JCI179617 (PMC11996917; doi:10.1172/JCI179617)

## Full unedited gel for Figure 1E

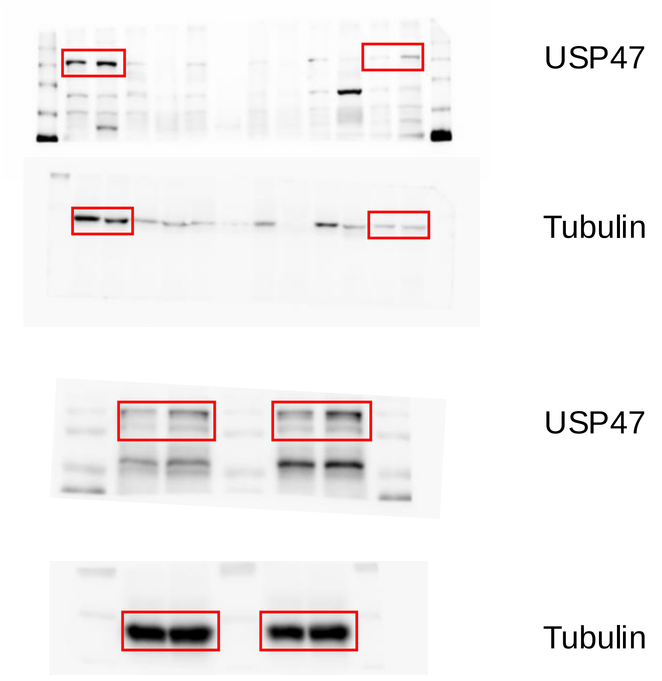


## Full unedited gel for Figure 2A

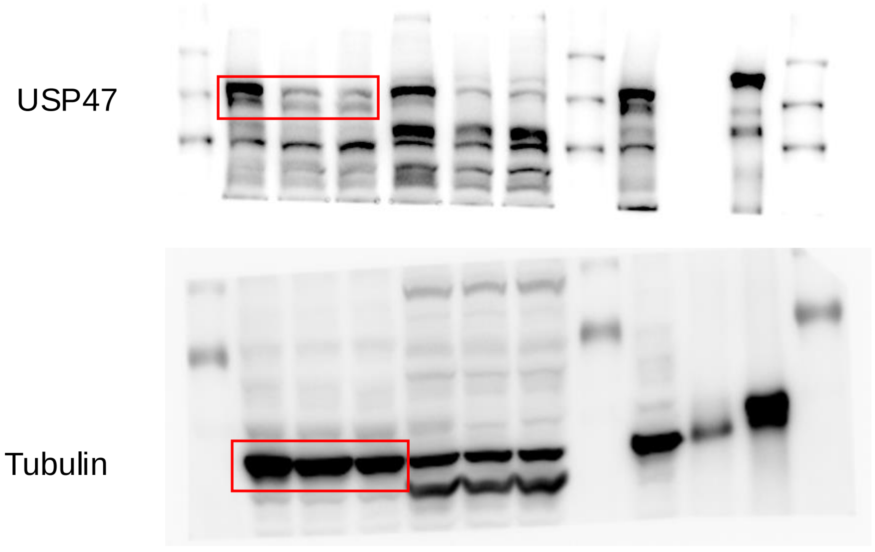

# Full unedited gel for Figure 3F

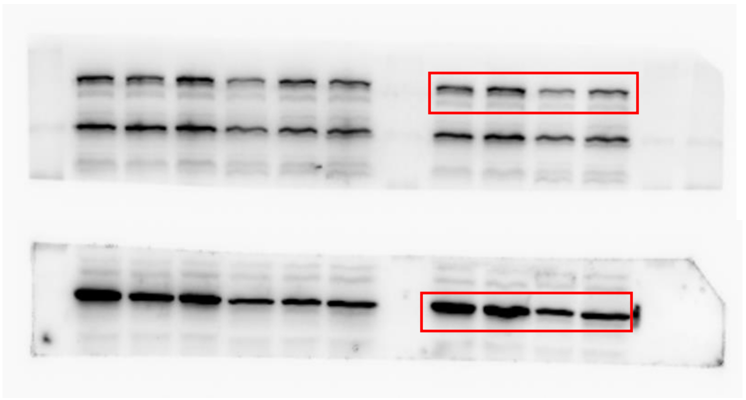

USP47

Tubulin

# Full unedited gel for Figure 4H

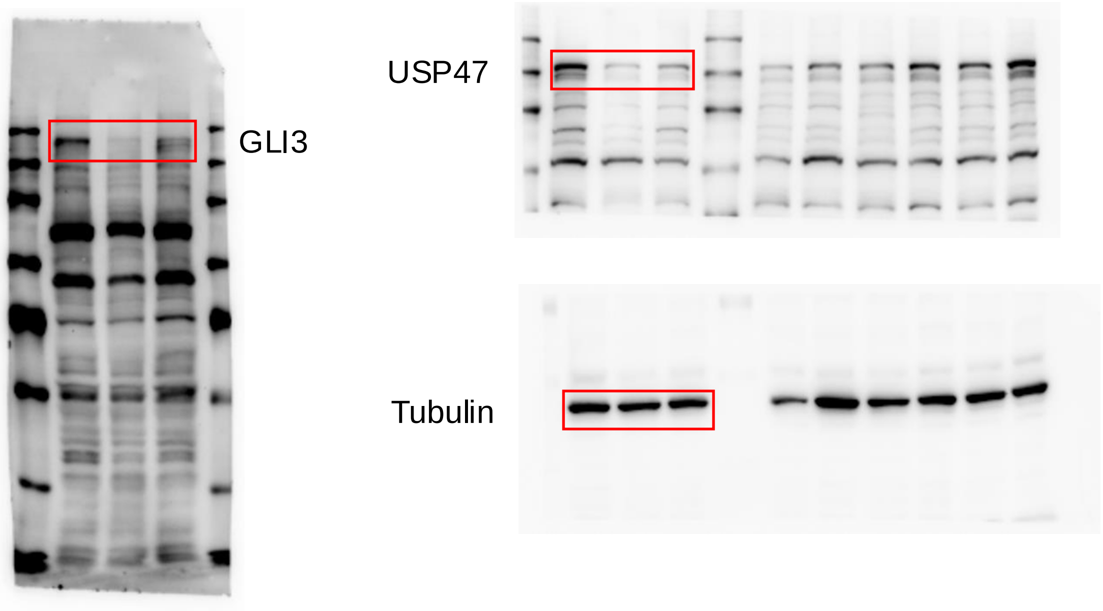


# Full unedited gel for Figure 4E

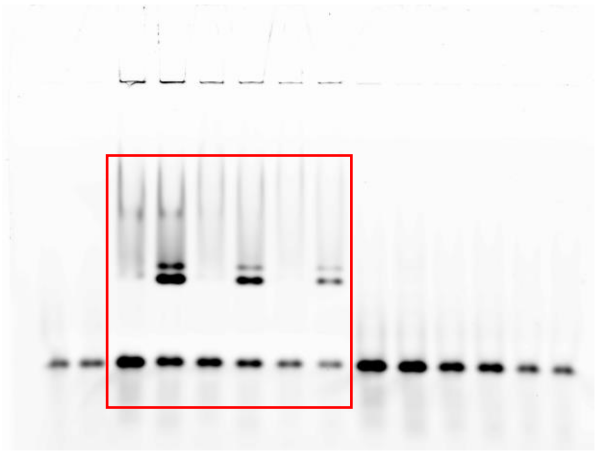

## Full unedited gel for Figure 5H

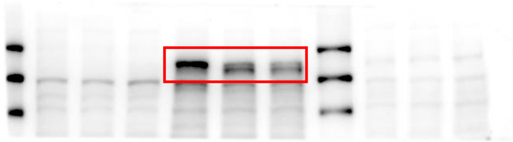

GLI3

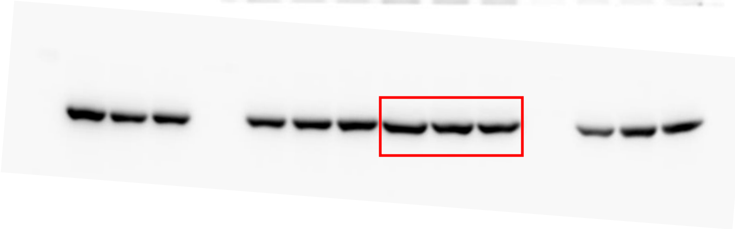

Tubulin

## Full unedited gel for Figure 6D

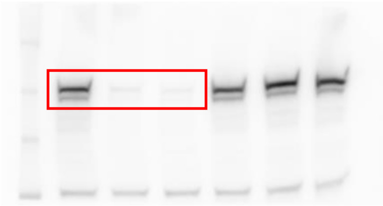

USP47

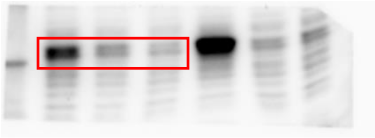

Snai1

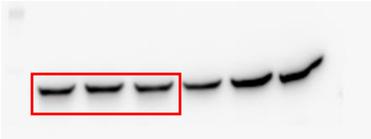

Tubulin

## Full unedited gel for Figure 6C

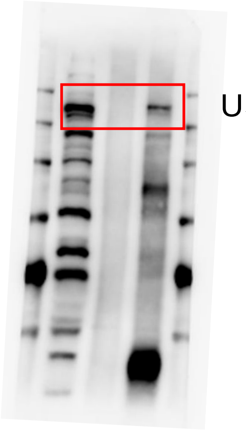

USP47

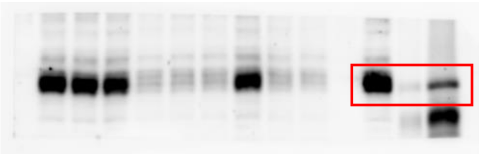

Snai1

## Full unedited gel for Figure 6E

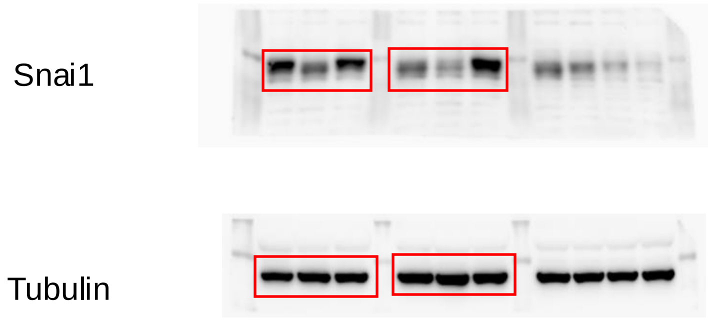


## Full unedited gel for Figure 6F

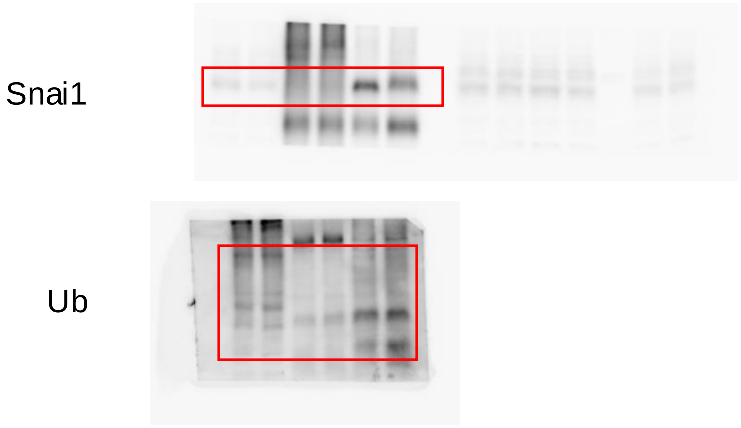


## Full unedited gel for Figure 6G

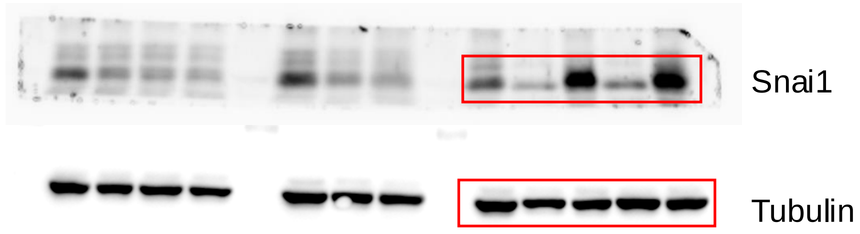

## Full unedited gel for Figure S6A

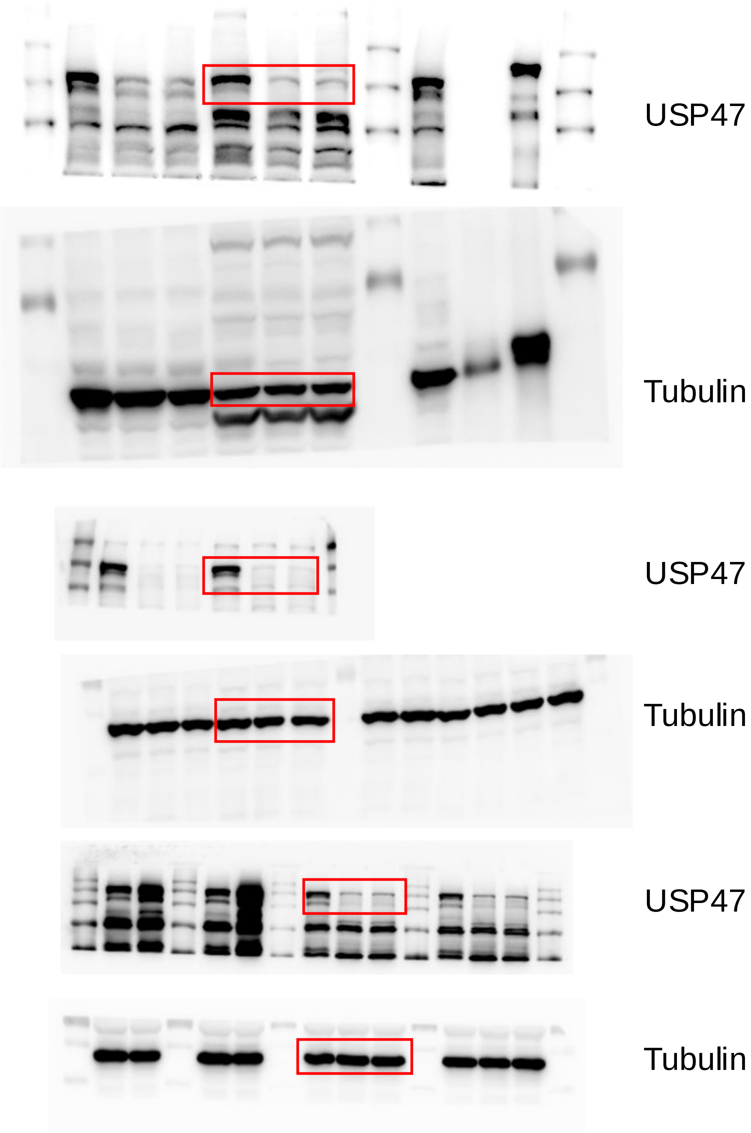


## Full unedited gel for Figure S7A

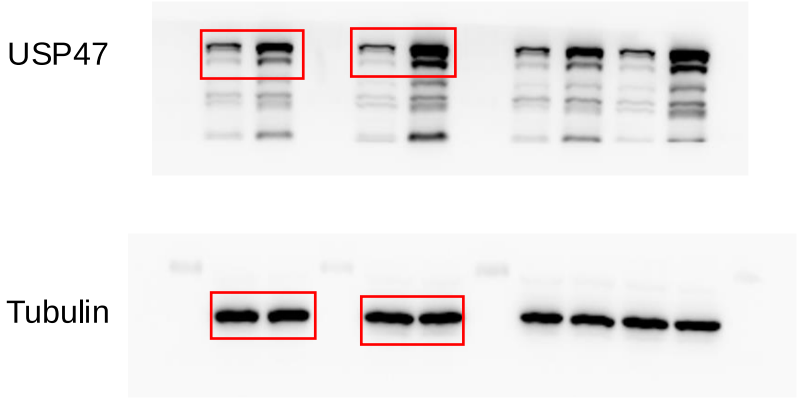

Full unedited gel for Figure S10B

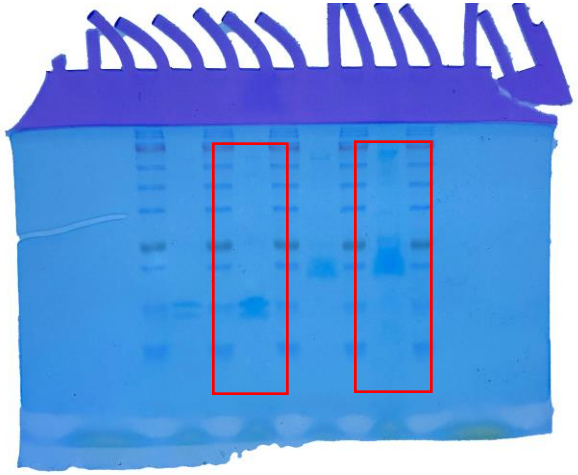

Full unedited gel for Figure S10C

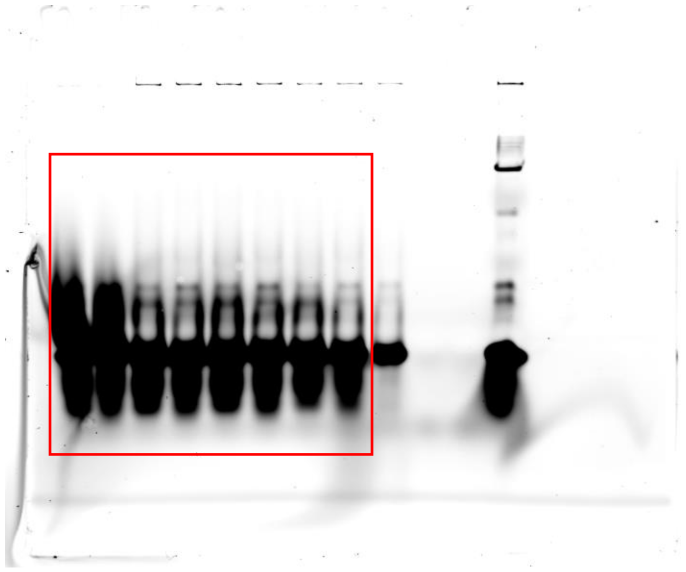

Full unedited gel for Figure S11A

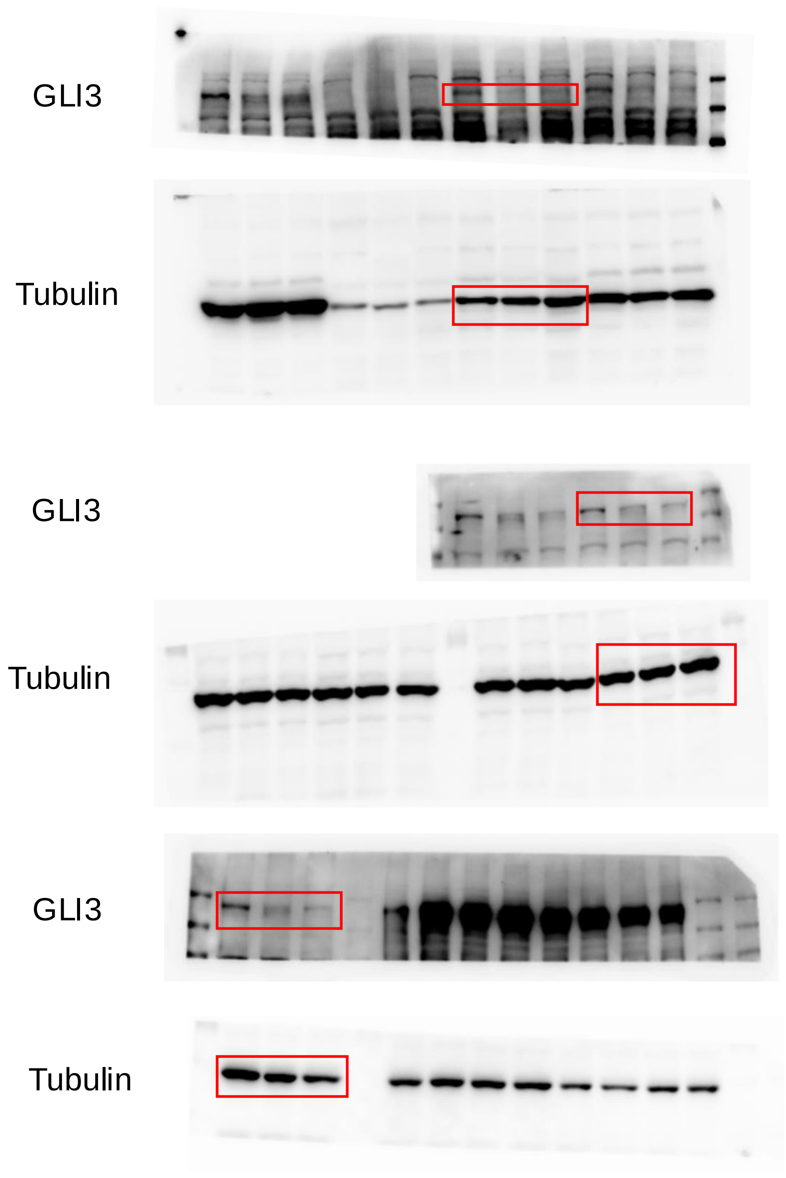

## Full unedited gel for Figure S12C

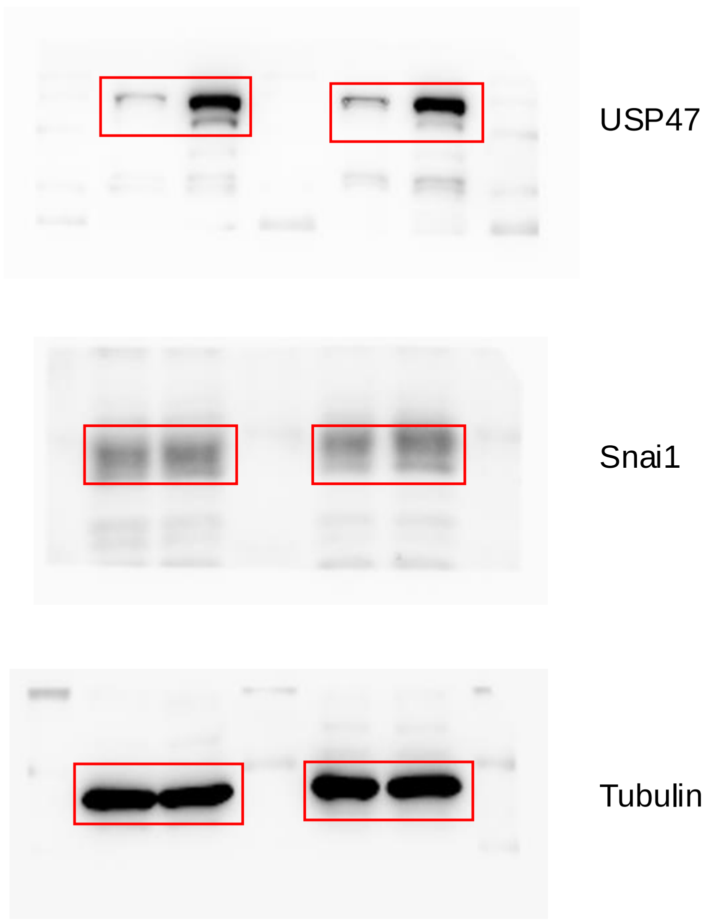


## Full unedited gel for Figure S12D

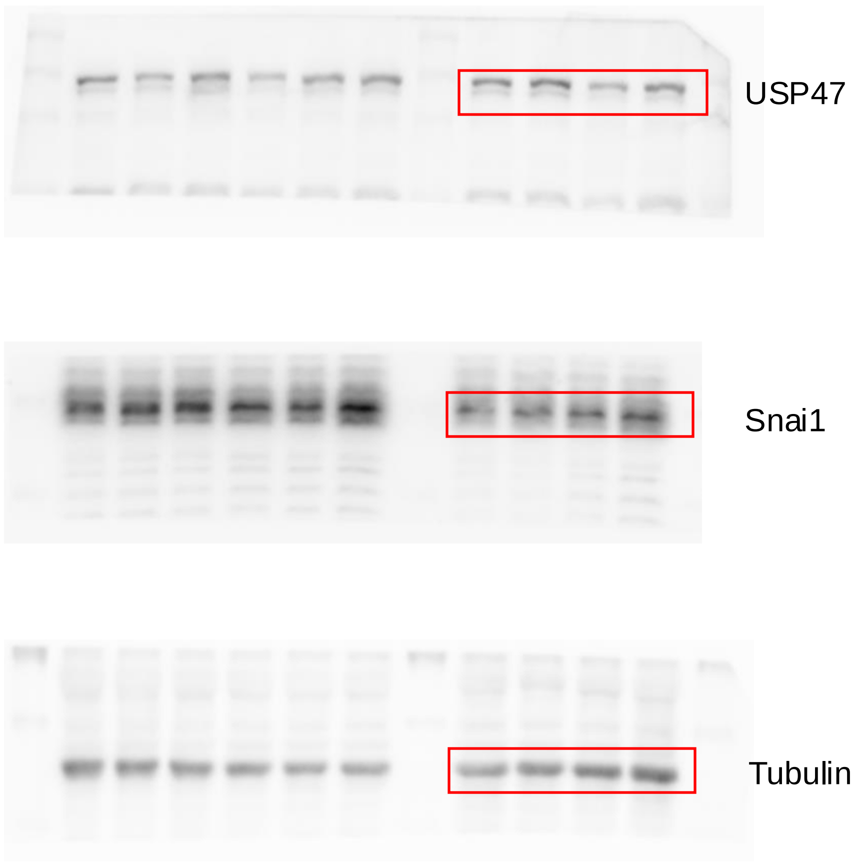

Full unedited gel for Figure S12E

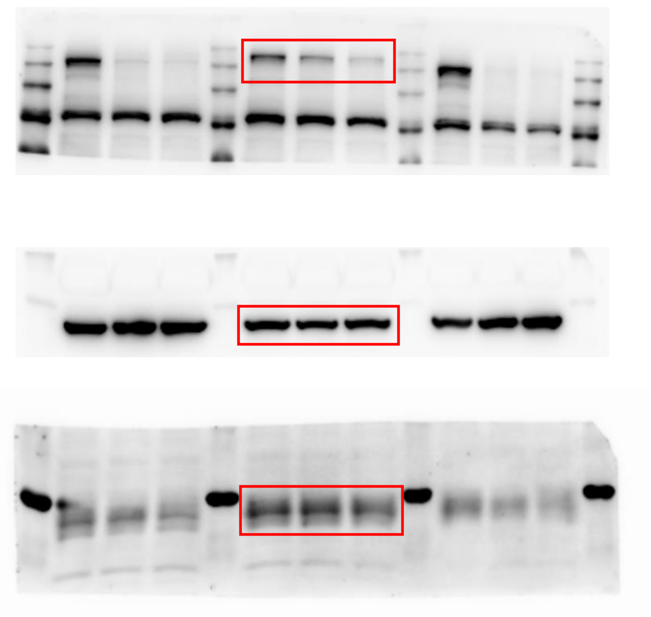


Full unedited gel for Figure S12F

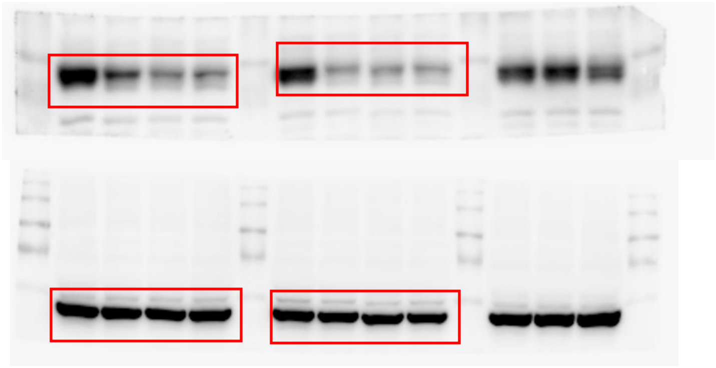

## Full unedited gel for Figure S12G

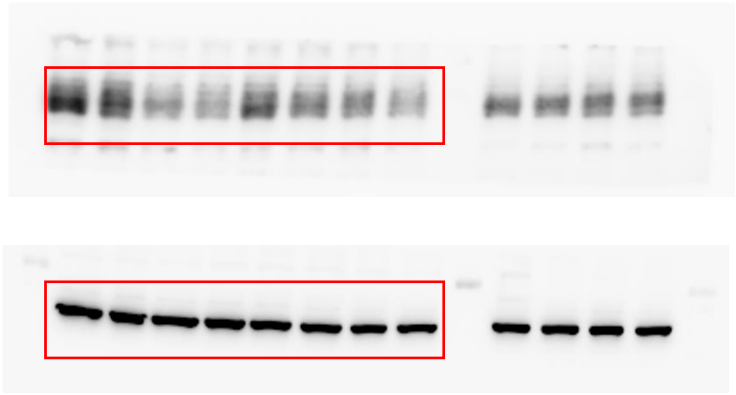

Snai1

Tubulin

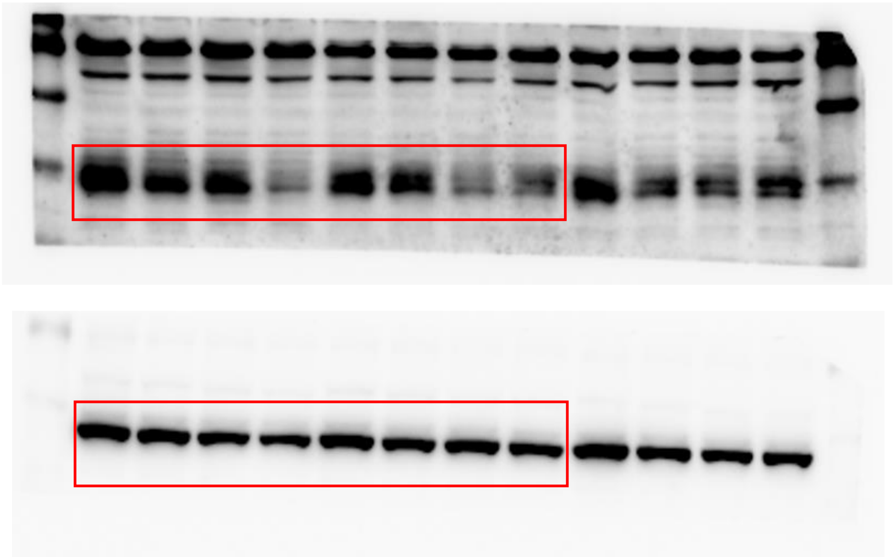

Snai1

Tubulin

## Full unedited gel for Figure 13A

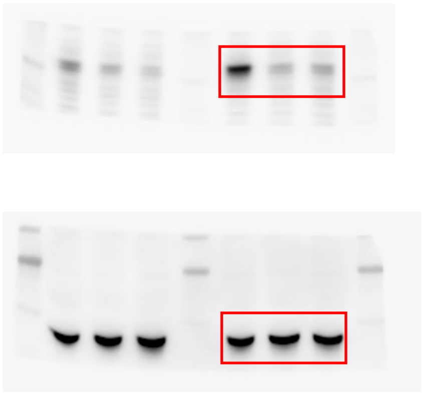

Snai1

Tubulin

## Full unedited gel for Figure S14A

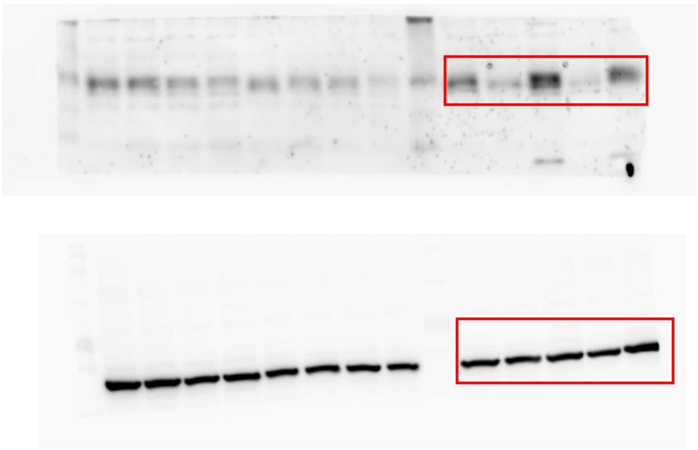

Snai1

Tubulin

Full unedited image for Figure 2C

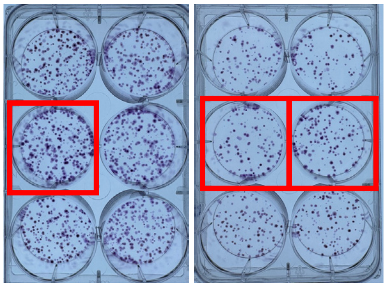

Full unedited image for Figure 5J

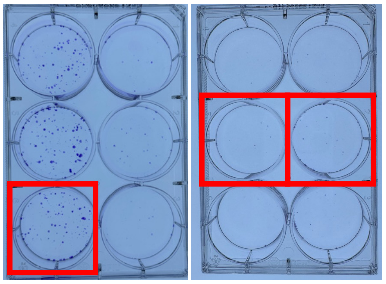

Full unedited image for Figure 7B

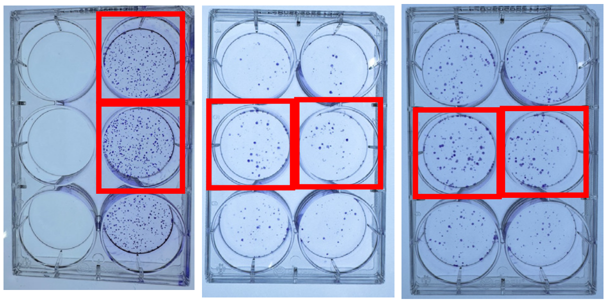

Full unedited image for Figure S6C

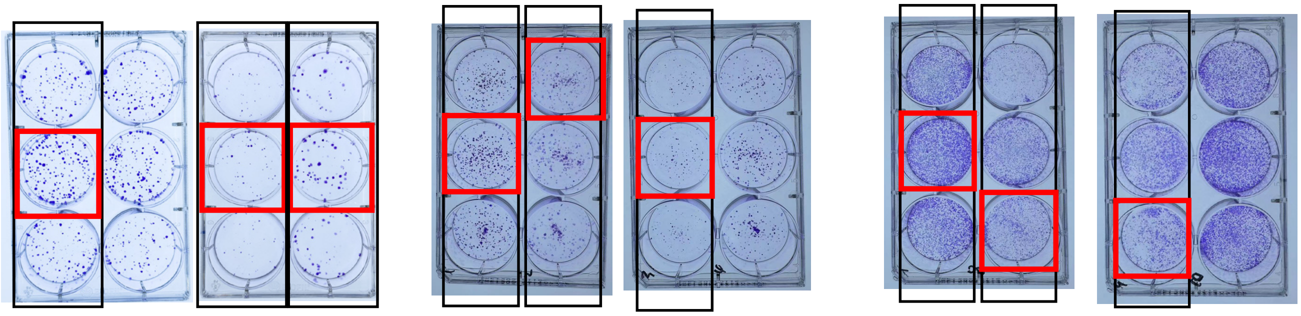

Full unedited image for Figure S7C

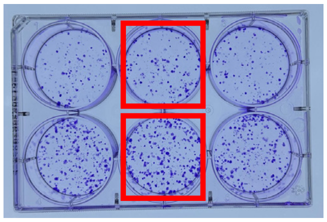

Full unedited image for Figure S7D

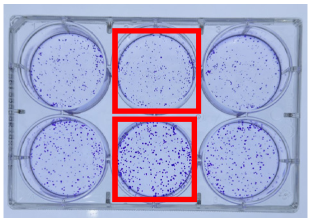

Full unedited image for Figure S9C

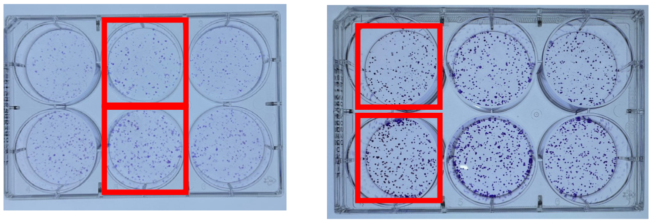

Full unedited image for Figure S11C

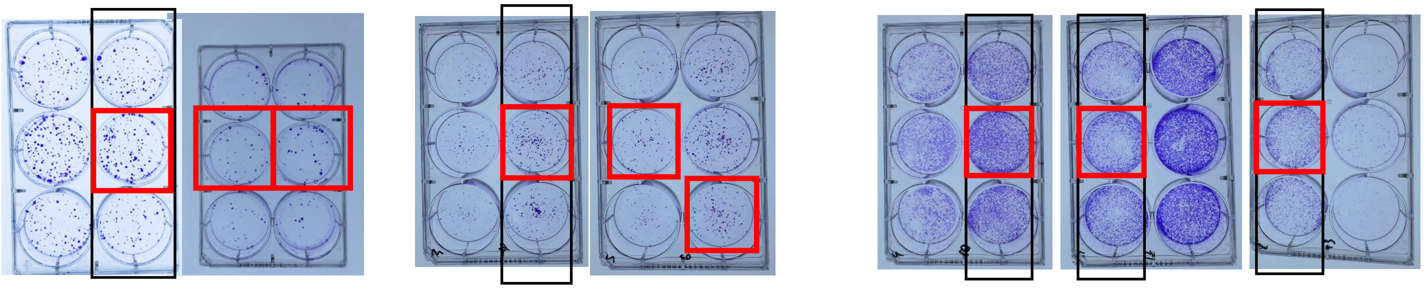

Full unedited image for Figure S13C

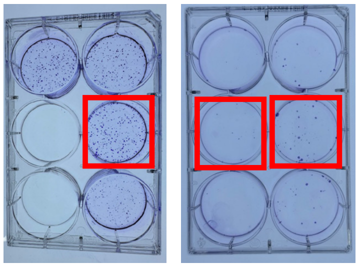

Full unedited image for Figure S14C

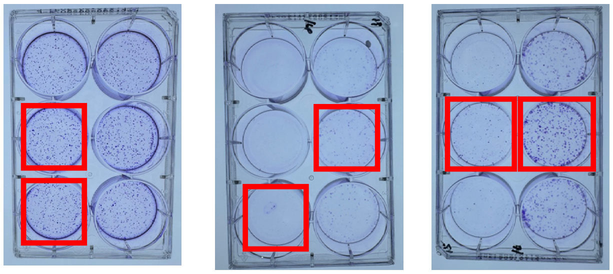

Full unedited image for Figure 2D

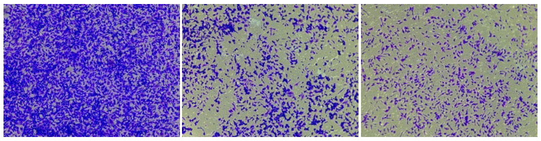

Full unedited image for Figure 2E

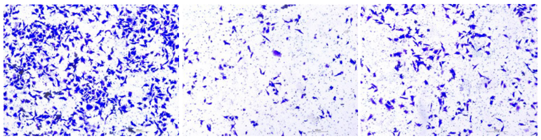

Full unedited image for Figure 5K

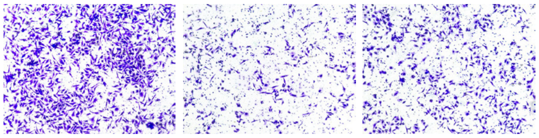

Full unedited image for Figure 5L

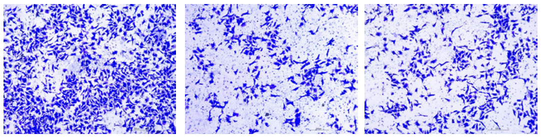

Full unedited image for Figure 7C

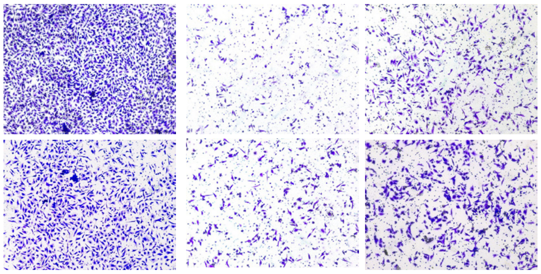

Full unedited image for Figure 7D

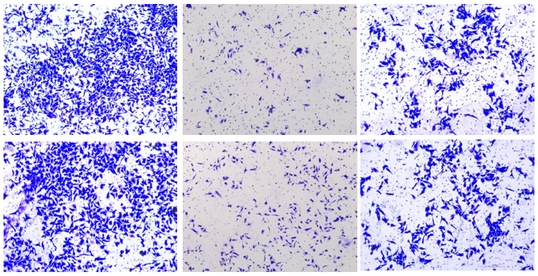

Full unedited image for Figure S6D

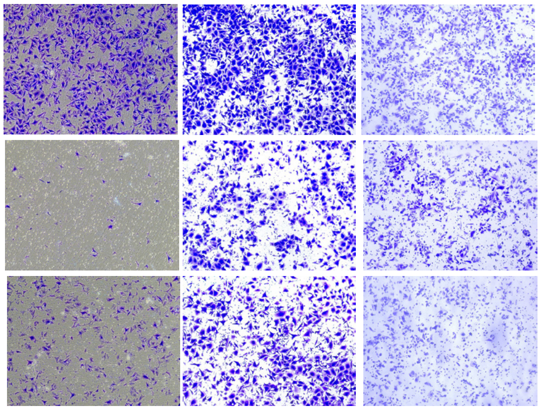

Full unedited image for Figure S6E

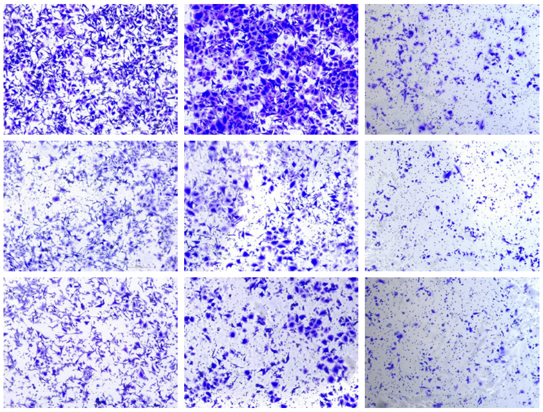

Full unedited image for Figure S7E

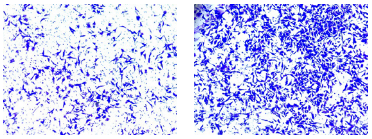

Full unedited image for Figure S7F

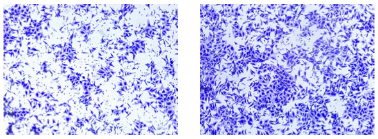

Full unedited image for Figure S7G

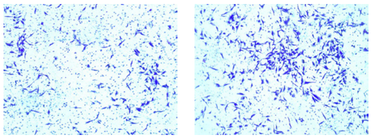

Full unedited image for Figure S7H

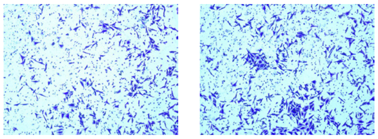

Full unedited image for Figure S9D

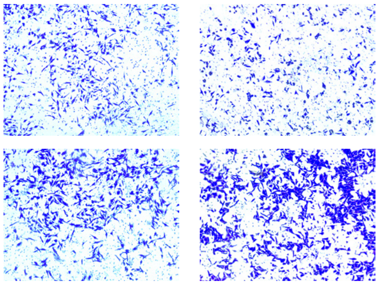

Full unedited image for Figure S9E

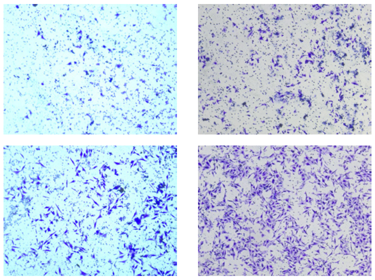

Full unedited image for Figure S11D

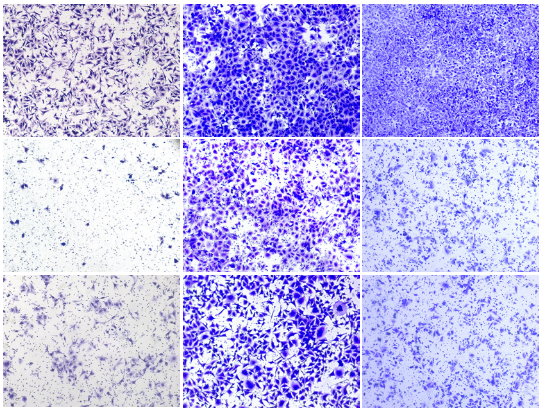

Full unedited image for Figure S11E

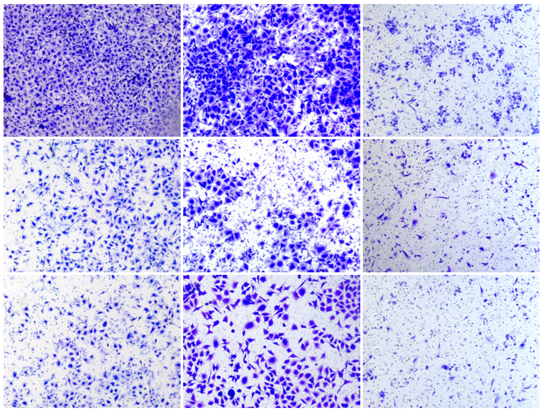

Full unedited image for Figure S13D

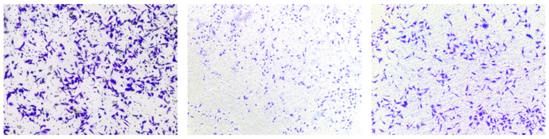

Full unedited image for Figure S13E

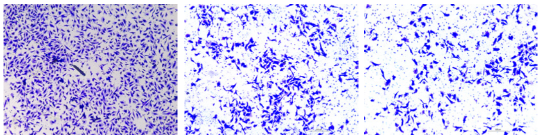

Full unedited image for Figure S14D

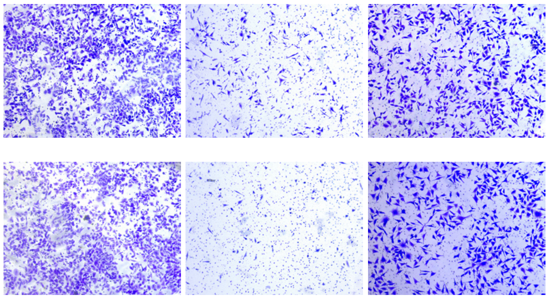

Full unedited image for Figure S14E

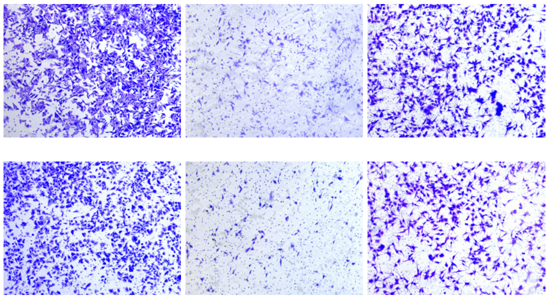

Full unedited image for Figure 2F

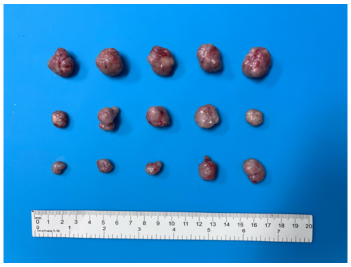

Full unedited image for Figure 5M

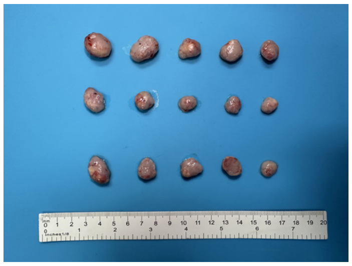

Full unedited image for Figure S6F

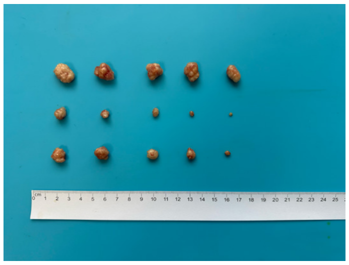

Full unedited image for Figure S7I

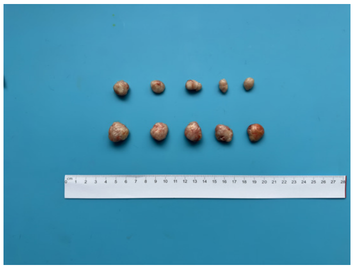

Full unedited image for Figure S7J

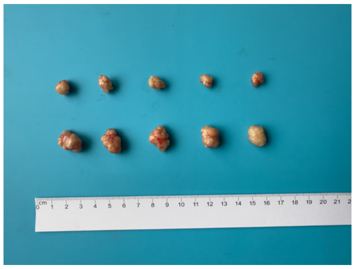

Full unedited image for Figure S11F

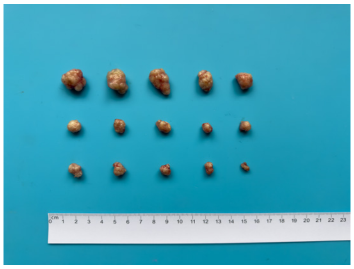

Full unedited image for Figure S6G

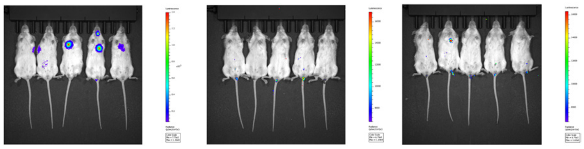

Full unedited image for Figure S6H

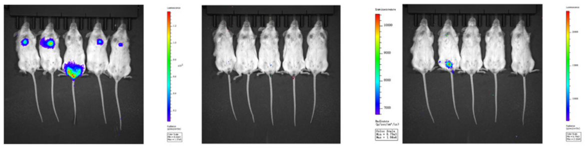

Full unedited image for Figure S7K

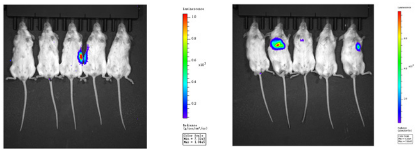

Full unedited image for Figure S7L

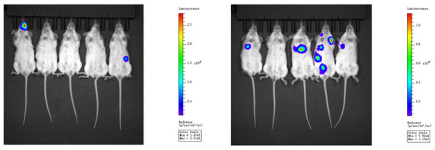

Full unedited image for Figure S11G

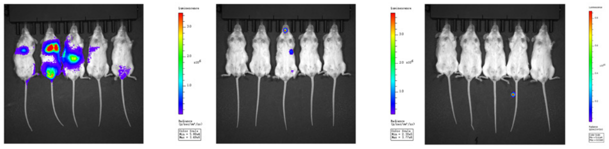

Full unedited image for Figure S11H

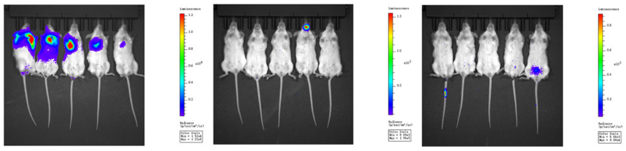

Supplement: Unedited blot and gel images [file jci-135-179617-s069.pdf]
