# Supplementary material for: An inherited predisposition allele promotes gastric cancer via enhancing deubiquitination-mediated activation of epithelial-to-mesenchymal transition signaling
Source: J Clin Invest. 2025 Feb 25;135(8):e179617. doi: 10.1172/JCI179617 (PMC11996917; doi:10.1172/JCI179617)
Supplement: Supplementary file 3 [file jci-135-179617-s024.pdf]

Tao.et al.Figure S1

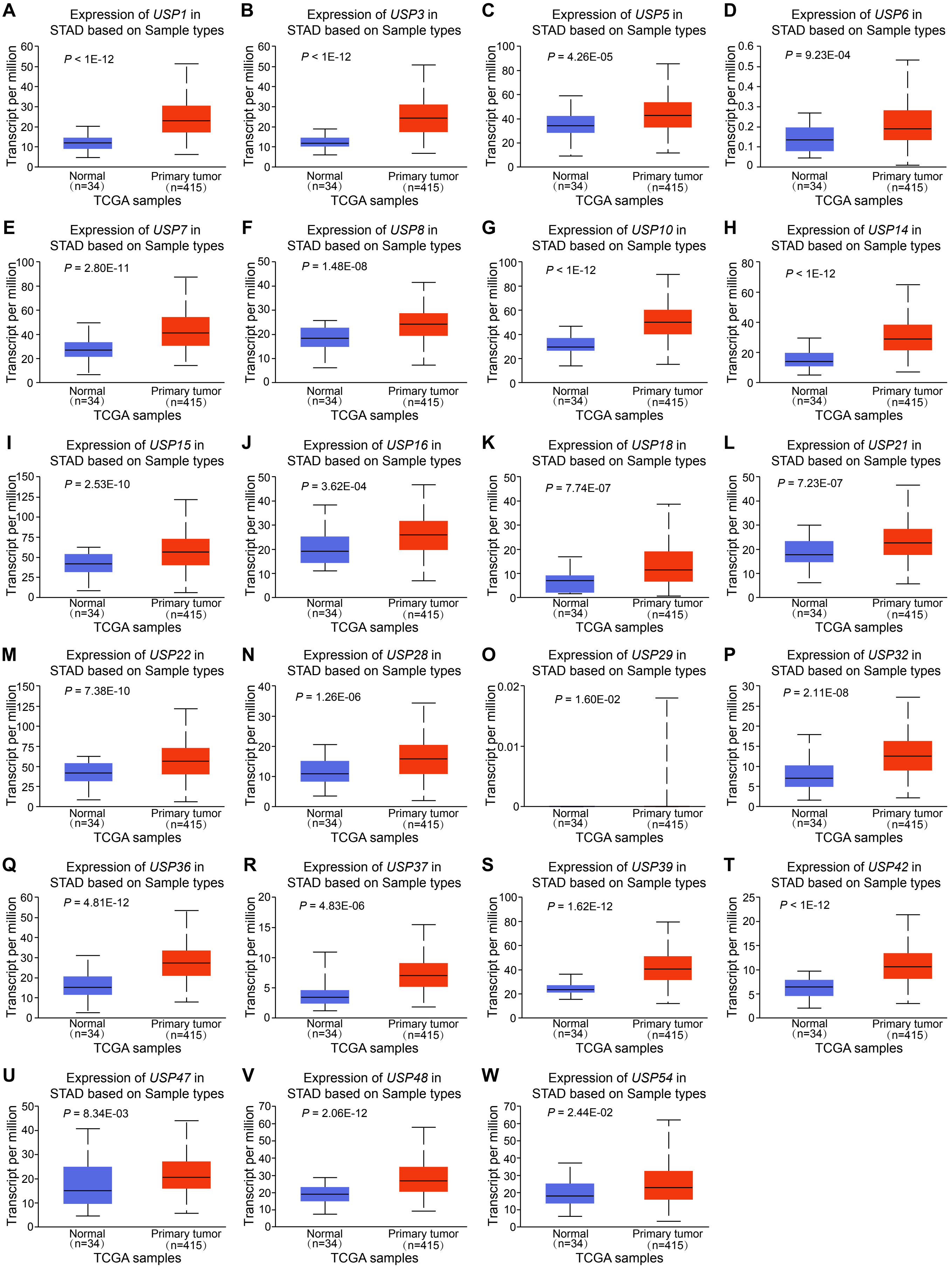

Supplemental Figure 1. 23 USPs characterized as oncogenes with elevated mRNA expression in GC tissues relative to normal gastric counterparts within the GEPIA dataset.

A-W. The GEPIA dataset showed significantly elevated expression in GC tissues of 23 USPs characterized as oncogenes, in gastric cancer tissues compared to normal tissues. Data represented as means ± SD, statistically assessed by a two-tailed t-test.

Tao.et al.Figure S2

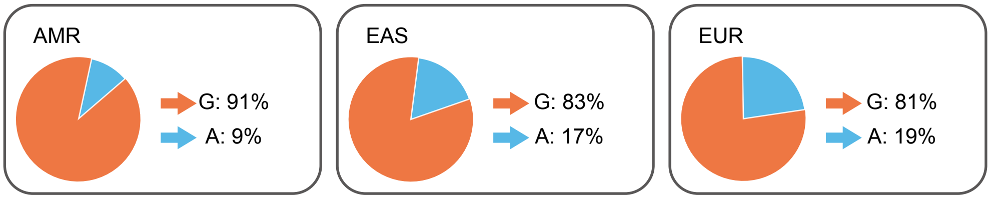

Supplemental Figure 2. The minor allele frequency (MAF) of rs72856331 across different populations.

AMR: American population; EAS: East Asian population; EUR: European population.

Tao.et al.Figure S3

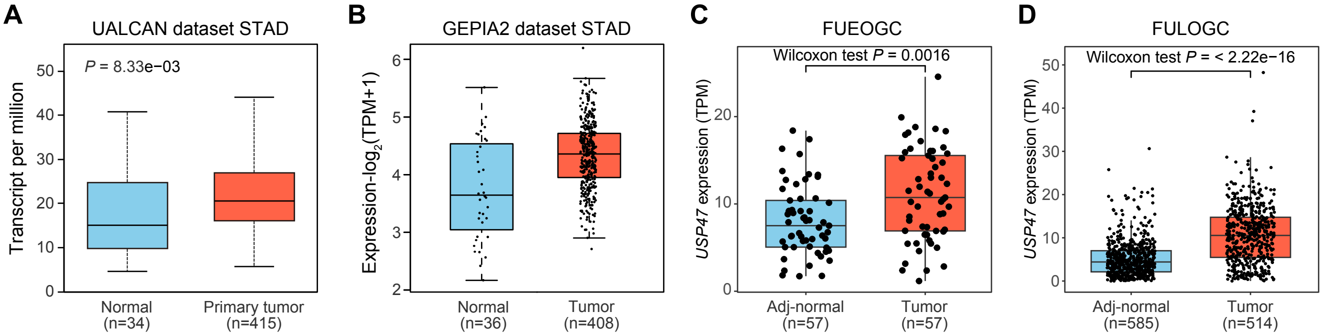

Supplemental Figure 3. Comparative analysis of *USP47* expression in gastric cancer tissues versus normal tissues across multiple datasets.

A. *USP47* expression data from the UALCAN database.

B. *USP47* expression data from the GEPIA2 database.

C and **D**. *USP47* expression data from our in-house cohorts of early-onset (FUEOGC) and late-onset (FULOGC) gastric cancer. Statistical significance was assessed using the Wilcoxon test, with $P < 0.05$ considered significant.

Tao.et al.Figure S4

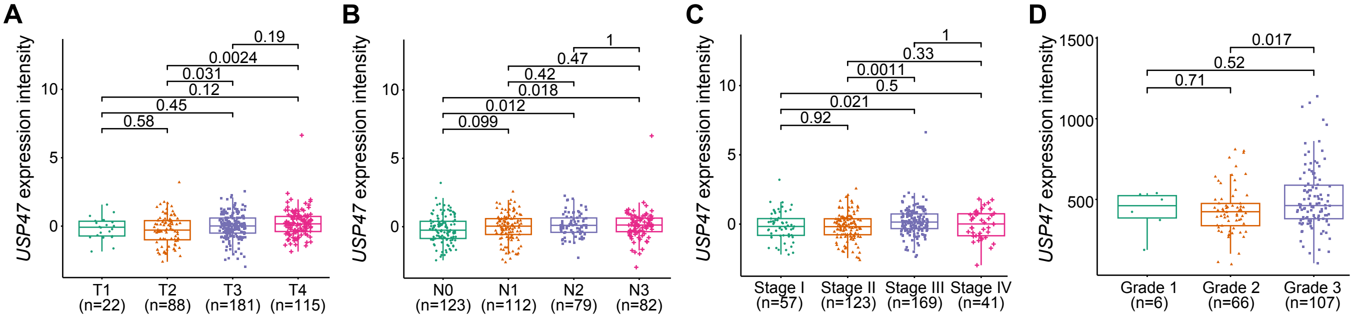

Supplemental Figure 4. Pairwise comparisons of *USP47* expression levels across clinical status of gastric cancer in the TCGA STAD cohort.

A-**D**: Pairwise comparisons of *USP47* expression levels in relation to GC clinical features, including (**A**) tumor invasiveness (n=406); (**B**) lymph node metastasis (n=394); (**C**) tumor stages (n=390), and (**D**) tumor grade (n=179). Statistical significance was determined by Dunn's post-hoc tests, with a significance threshold set at $P < 0.05$.

Tao.et al.Figure S5

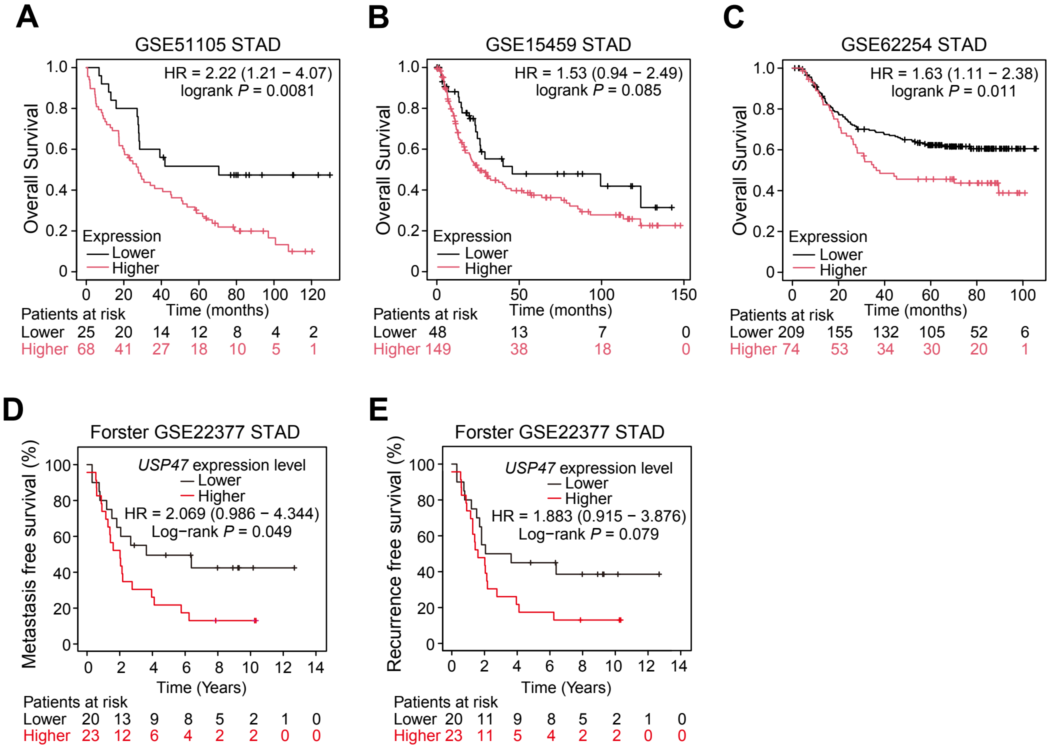

Supplemental Figure 5. Elevated *USP47* expression is associated with poorer survival outcomes across multiple gastric cancer cohorts.

A-**C**: Kaplan-Meier survival curves demonstrate that higher *USP47* expression is significantly associated with poorer overall survival in the GSE51105 (**A**), GSE15459 (**B**), and GSE62254 (**C**) gastric cancer cohorts.

D and **E**. Elevated *USP47* expression is linked to reduced metastasis-free survival (**D**) and recurrence-free survival (**E**) in the GSE22377 cohort. *P*-values were calculated using the log-rank test.

Tao.et al.Figure S6

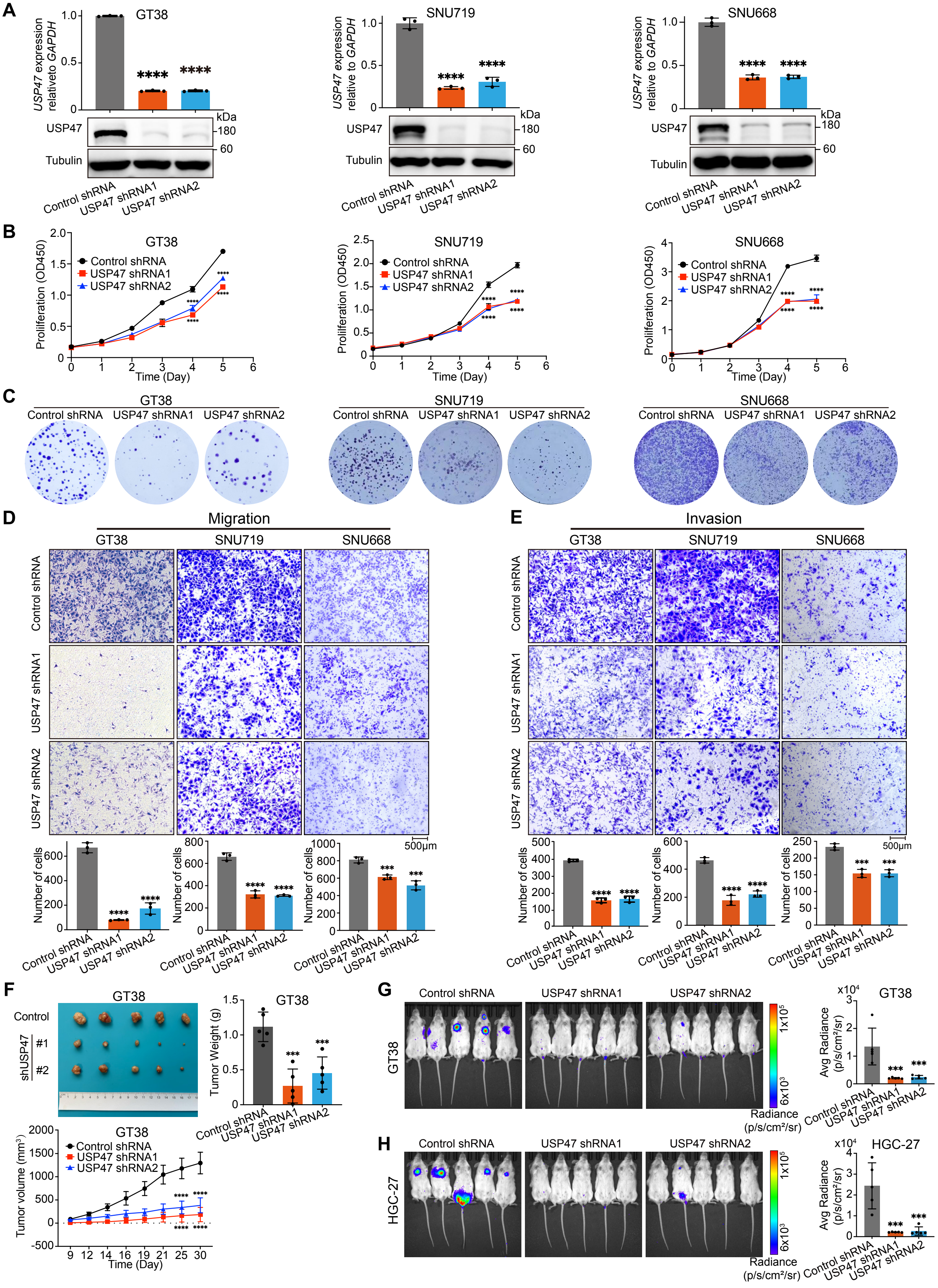

Supplemental Figure 6. Impact of *USP47* knockdown on gastric cancer growth, metastasis, and tumorigenesis.

A. Knockdown efficiency of *USP47* in GT38, SNU719, and SNU668 cell lines validated by RT-qPCR and Western blotting using two independent *USP47*-specific shRNAs (n=3).

B-**E**. *USP47* knockdown suppresses gastric cancer cell phenotypes, as demonstrated by CCK-8 proliferation assays (**B**), colony formation assays (**C**), migration (**D**) and invasion assays (**E**) in GT38, SNU719, and SNU668 cells under different treatments (n=3). Scale bars: 500 μm.

F. Representative images, growth curves, and tumor weights of xenograft tumors derived from nude mice injected with GT38 cells with *USP47* knockdown or control cells, assessed after a 4-week experimental period (n=5 per group). Significance assessed by 2-way ANOVA and Holm-Šidák post hoc test for growth curves and 1-way ANOVA with Holm-Šidák multiple comparison test for tumor weights.

G and **H**: *USP47* knockdown suppresses in vivo metastatic potential in gastric cancer cells. Bioluminescence images were captured and quantitatively analyzed 6 weeks after tail vein injection of GT38 cells (**G**) and HGC-27 cells (**H**) transfected with either control shRNA or *USP47*-specific shRNAs into NOD/SCID mice (n=5 per group).

Data are presented as mean ± SD. Statistical significance was calculated using 2-way ANOVA and Holm-Šidák post hoc test for panel **B** or 1-way ANOVA with Holm-Šidák multiple comparison test for panels **D, E, G** and **H.** $^{**}P < 0.01$, $^{***}P < 0.001$, $^{****}P < 0.0001$.

Tao.et al.Figure S7

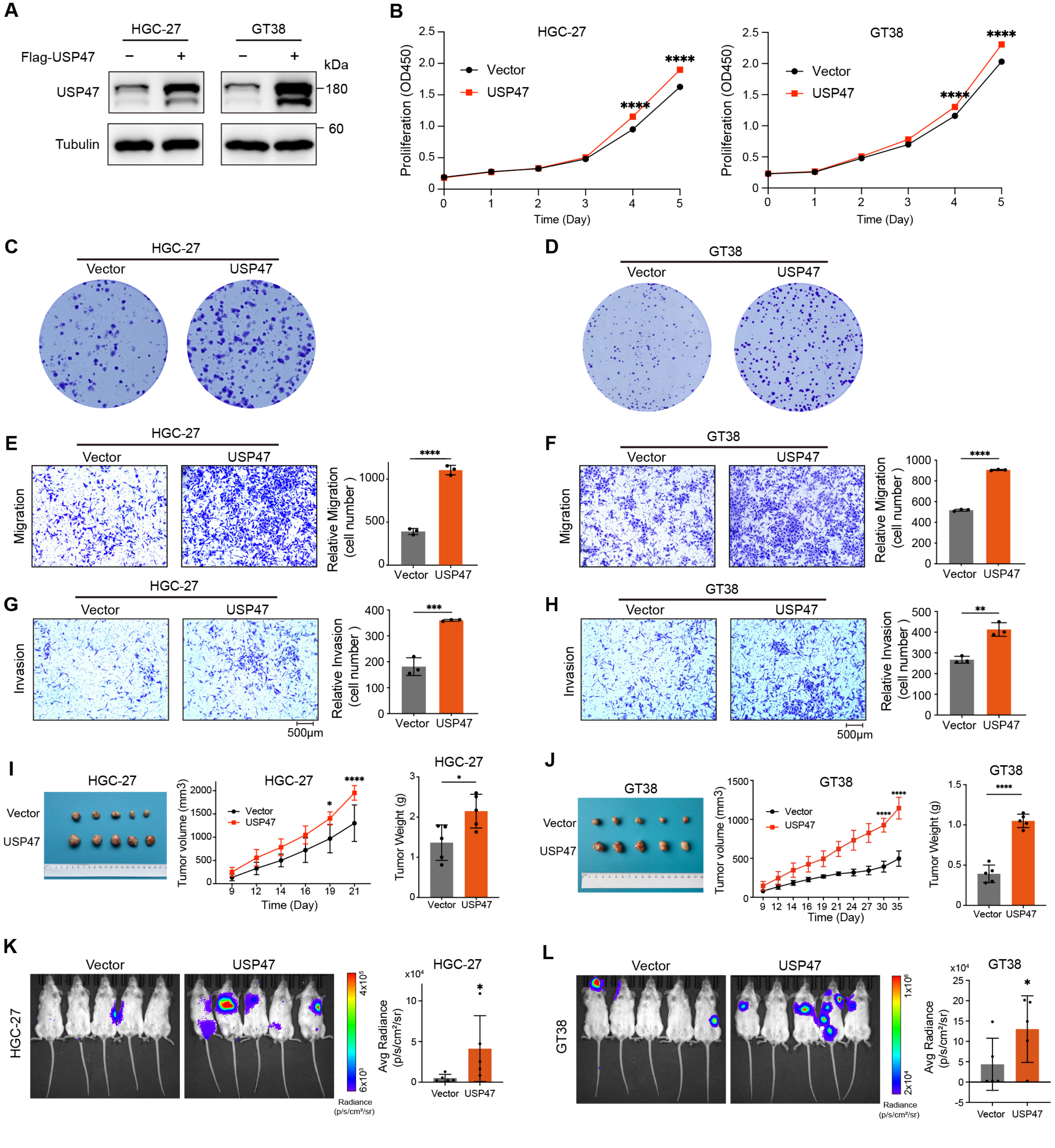

Updated image 10/1/2026

Supplemental Figure 7. *USP47* overexpression promotes gastric cancer growth, metastasis, and tumorigenesis.

A. Western blot analysis validating *USP47* overexpression in HGC-27 and GT38 cells.

B-**H**. *USP47* overexpression enhances gastric cancer cell phenotypes, including proliferation (**B**), colony formation (**C** and **D**), migration (**E** and **F**), and invasion (**G** and **H**) in HGC-27 and GT38 cells (n=3). Scale bars: 500 μm.

I and **J**. *USP47* overexpression significantly accelerates tumor growth in xenograft models using HGC-27 (**I**) and GT38 (**J**) cells, as shown by tumor volume measurements over time (n=5 per group). Significance assessed by 2-way ANOVA for growth curves and a two-tailed student's t-test for tumor weights.

K and **L**. Bioluminescence imaging and quantitative analysis of 6 weeks post tail vein injection of *USP47*-overexpressing HGC-27 (**K**) and GT38 (**L**) cells into NOD/SCID mice demonstrate enhanced metastatic potential (n=5 per group).

Data are presented as mean ± SD. Statistical significance was calculated using a two-tailed Student's t-test for panels **E**, **F**, **G**, **H**, **K** and **L**, or two-way ANOVA for panel **B**: $*P < 0.05$, $**P < 0.01$, $***P < 0.001$, $****P < 0.0001$.

Tao.et al.Figure S8

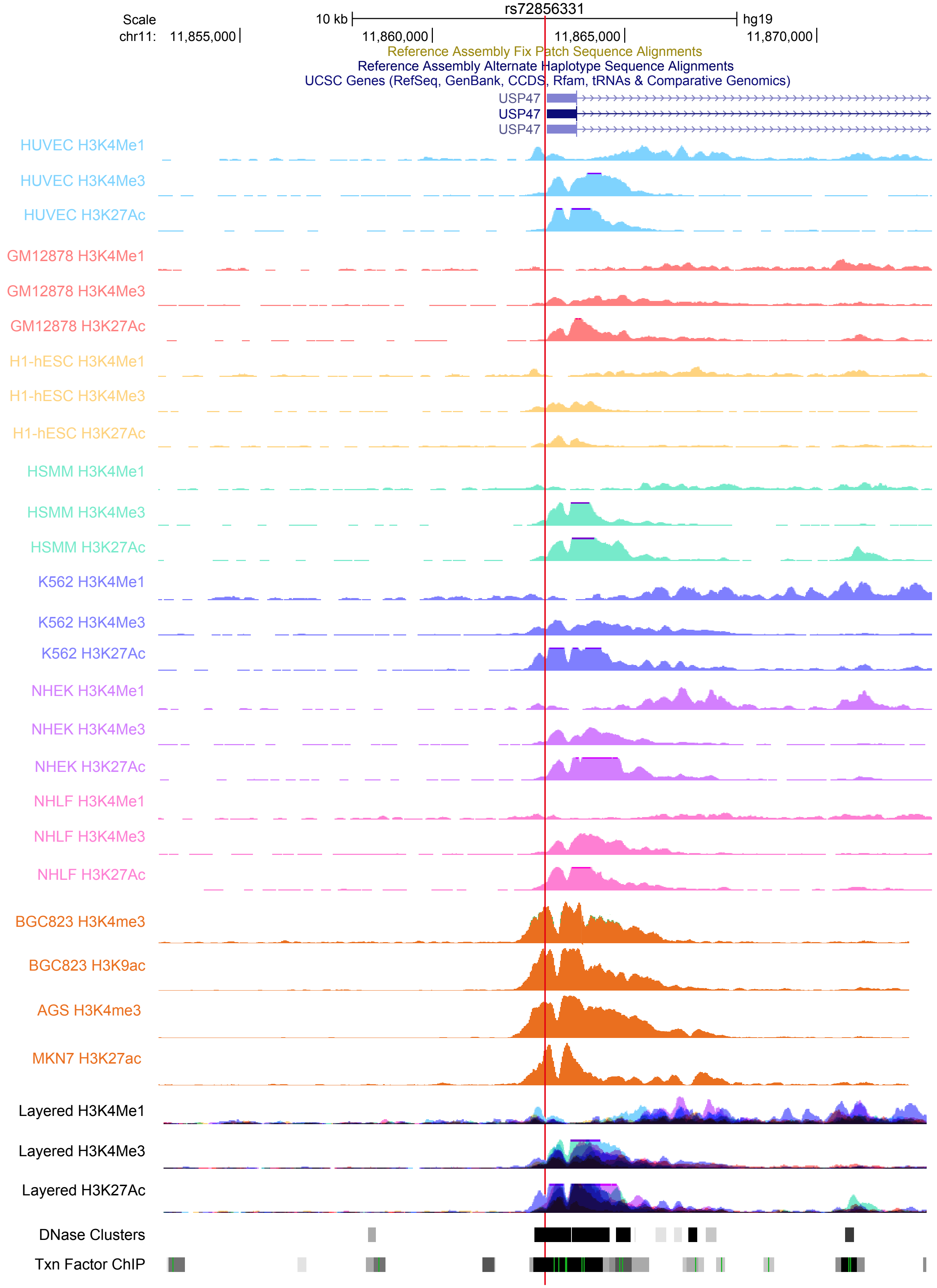

Supplemental Figure 8. The 72856331-containing region is a potential transcriptional regulatory element across gastric cancer cell lines.

The diagram illustrates the distribution of histone modifications (H3K4me1, H3K27ac, H3K4me3, H3K9ac), transcription factor binding sites, and DNase I hypersensitive sites surrounding rs72856331 (highlighted by a red line) in multiple gastric cancer cell lines, as sourced from the UCSC Genome Browser dataset.

Tao.et al.Figure S9

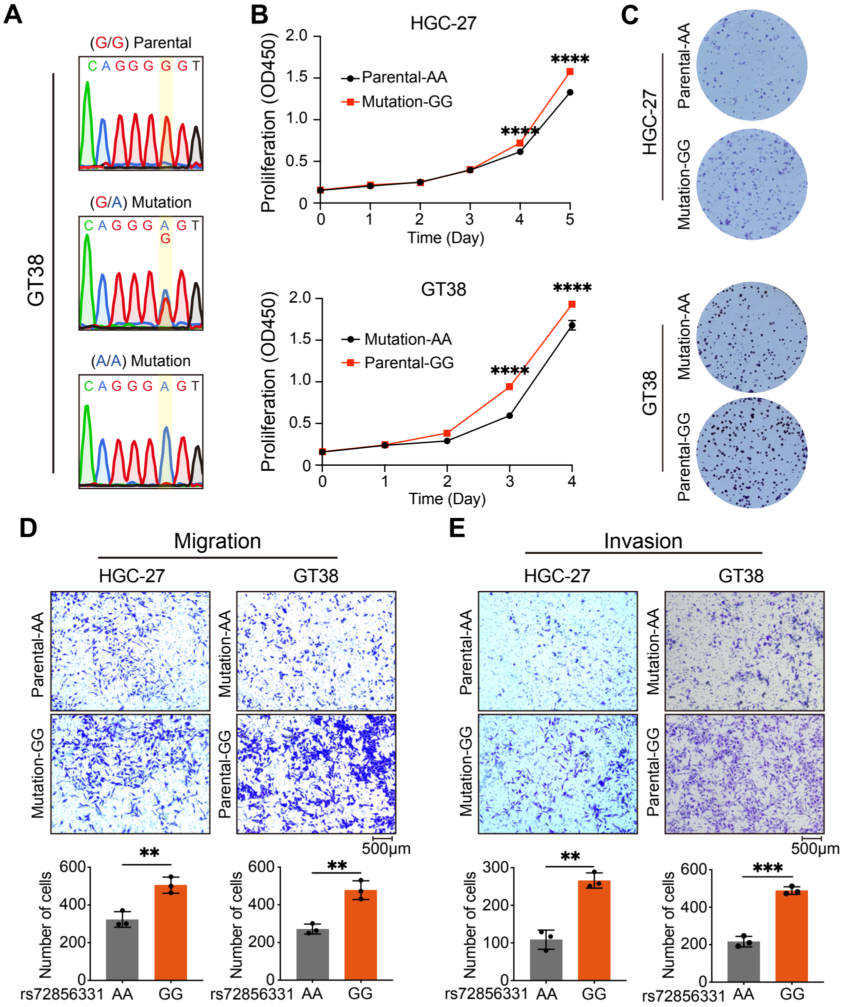

Supplemental Figure 9. The impact of rs72856331 genotype on gastric cancer cell growth, migration, and invasion.

A. Sanger sequencing confirming CRISPR/Cas9-modified genotypes in GT38 cells compared to parental controls.

B. Proliferation analysis of CRISPR/Cas9-modified HGC-27 and GT38 cells compared to parental cells (n=3).

C. Representative images of colony formation assays for HGC-27 and GT38 cells with different rs72856331 genotypes.

D and **E**. Migration (**D**) and invasion (**E**) assays for HGC-27 and GT38 cells with varying genotypes (n=3). Scale bars = 500 μm.

Data are presented as mean ± SD. Statistical significance was determined using two-tailed Student's t-test for panels **D** and **E**, or two-way ANOVA for panel **B**: $^{**}P < 0.01$, $^{***}P < 0.001$, $^{****}P < 0.0001$.

Tao.et al.Figure S10

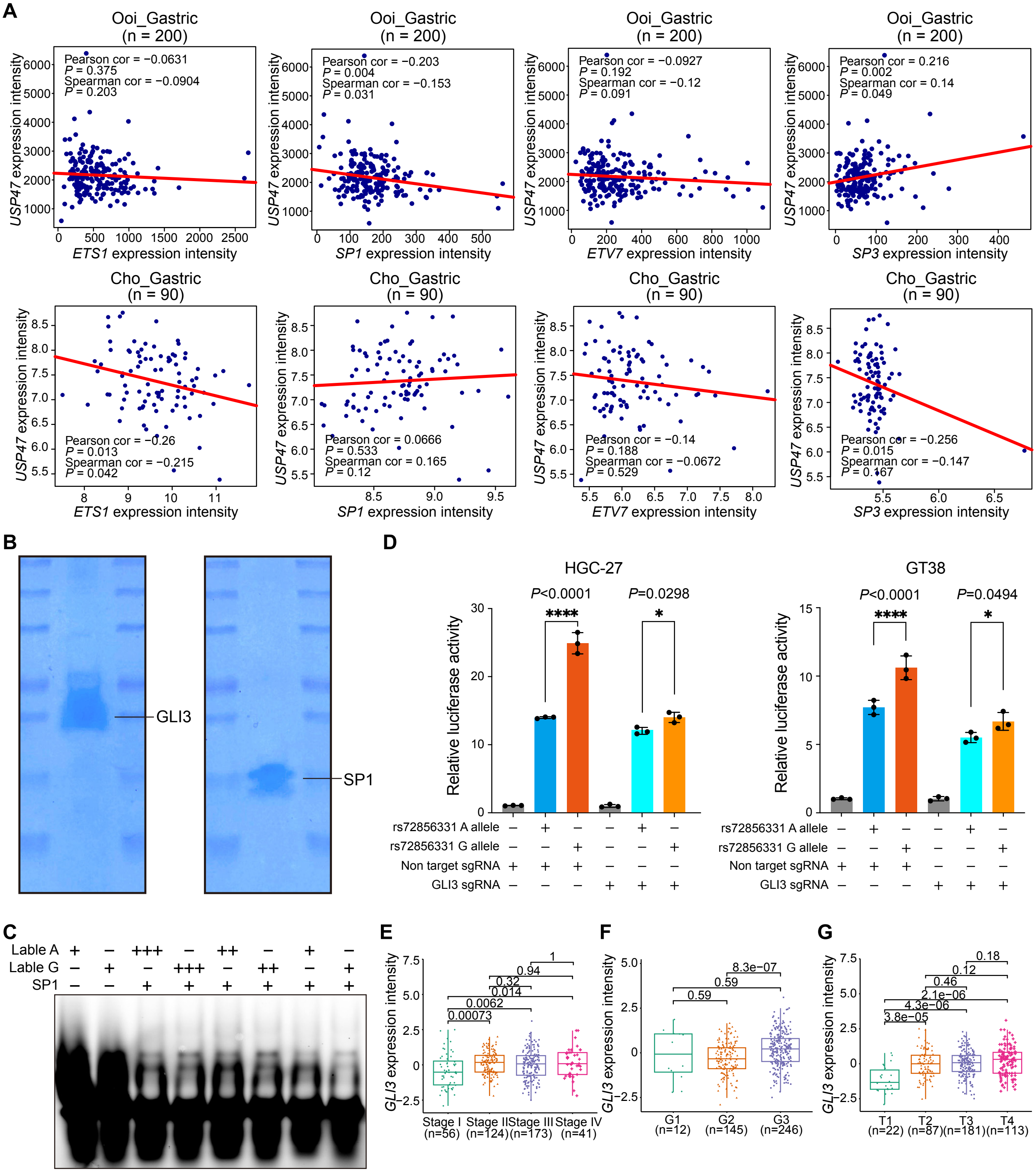

Supplemental Figure 10. Four transcription factors predicted by the Enhancer Element Locator (EEL) algorithm showed diminished potential to regulate *USP47* expression.

A. Scatterplots showing an expression correlation between four predicted transcription factors and *USP47* in the two independent GC cohorts. *P* values assessed by the Pearson correlation test.

B. Coomassie brilliant blue staining confirming the purity of his-tagged GLI3 and SP1 proteins used for subsequent experiments.

C. Electrophoretic mobility shift assay (EMSA) using purified SP1 protein demonstrates no differential binding affinity between the rs72856331 G allele and the A allele.

D. Relative luciferase activity in HGC-27 and GT38 cells transfected with reporter plasmids containing either the G or A allele of rs72856331, with or without GLI3-targeting sgRNAs (n=3). A statistical significance was calculated using 1-way ANOVA with Holm-Šidák multiple comparison test. *$P < 0.05$, ****$P < 0.0001$.

E-G. Pairwise comparisons of GLI3 expression levels with GC clinical features in the TCGA STAD cohort: (**E**) tumor stages (n=394), (**F**) tumor grade (n=403), and (**G**) tumor invasiveness (n=403). Statistical significance was determined by Dunn's post-hoc tests, with a significance threshold set at $P < 0.05$.

Tao.et al.Figure S11

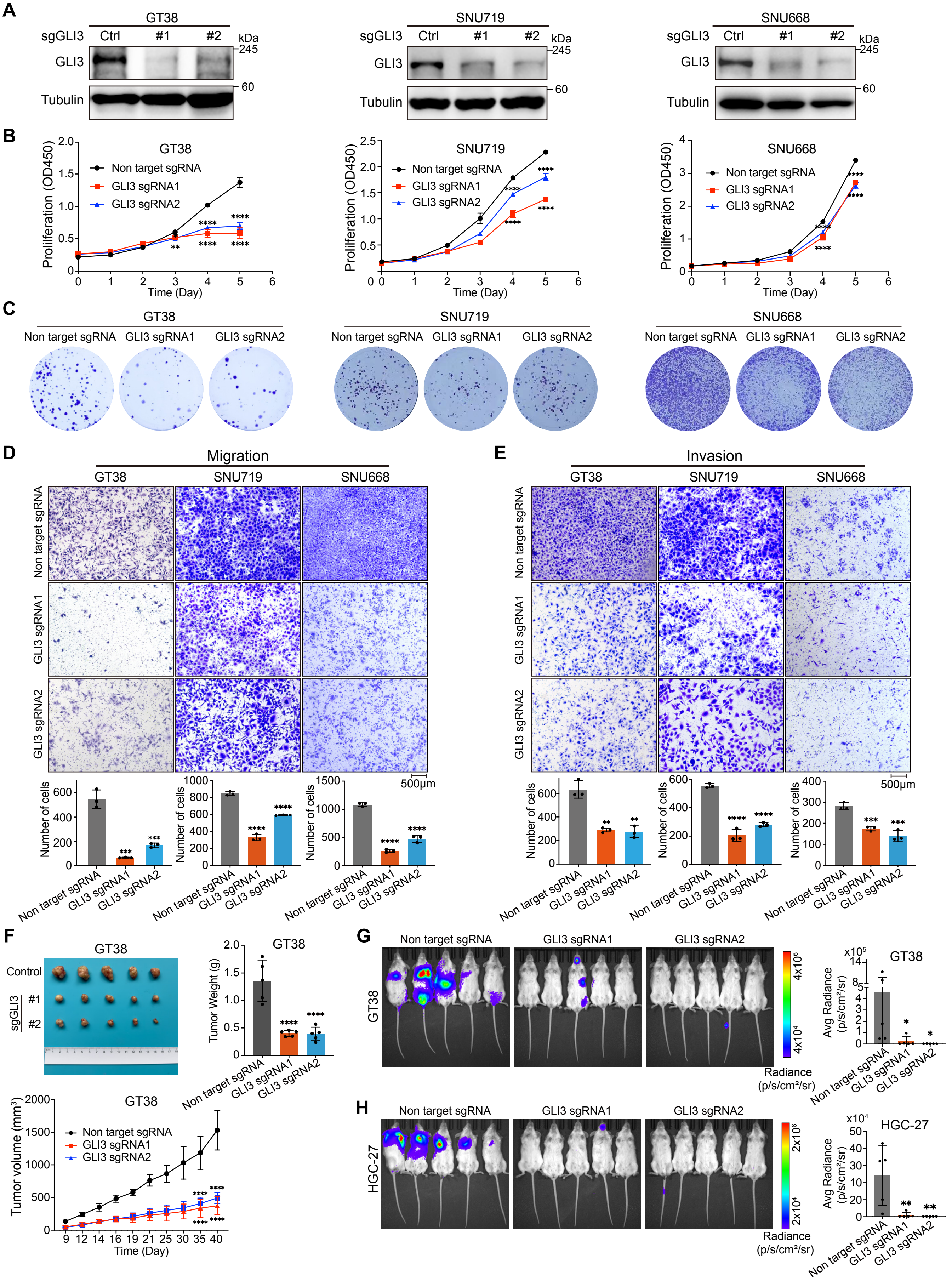

Supplemental Figure 11. Impact of GLI3 knockout on gastric cancer growth, metastasis, and tumorigenesis.

A. Knockout efficiency of GLI3 in GT38, SNU719, and SNU668 cell lines was validated by Western blotting using two independent GLI3-specific sgRNAs (n=3).

B-**E**. GLI3 knockout suppresses gastric cancer cell phenotypes, as demonstrated by CCK-8 proliferation assays (**B**), colony formation assays (**C**), migration (**D**) and invasion assays (**E**) in GT38, SNU719, and SNU668 cells under different treatments (n=3). Scale bars: 500 μm.

F. Representative images, growth curves, and tumor weights of xenograft tumors derived from nude mice injected with GLI3-knockout GT38 cells or control cells, assessed after a 6-week experimental period (n=5 per group). Significance assessed by 2-way ANOVA and Holm-Šidák post hoc test for growth curves and 1-way ANOVA with Holm-Šidák multiple comparison test for tumor weights.

G and **H**. Bioluminescence images were captured and quantitatively analyzed 6 weeks after tail vein injection of GT38 cells (**G**) and HGC-27 cells (**H**), transfected with control or GLI3-specific sgRNAs, into NOD/SCID mice.

Data are presented as mean ± SD. Statistical significance was calculated using 2-way ANOVA and Holm-Šidák post hoc test for panel **B,** or 1-way ANOVA with Holm-Šidák multiple comparison test for panels **D, E, G** and **H.** $^{*}P < 0.05$, $^{**}P < 0.01$, $^{***}P < 0.001$, $^{****}P < 0.0001$.

Tao.et al.Figure S12

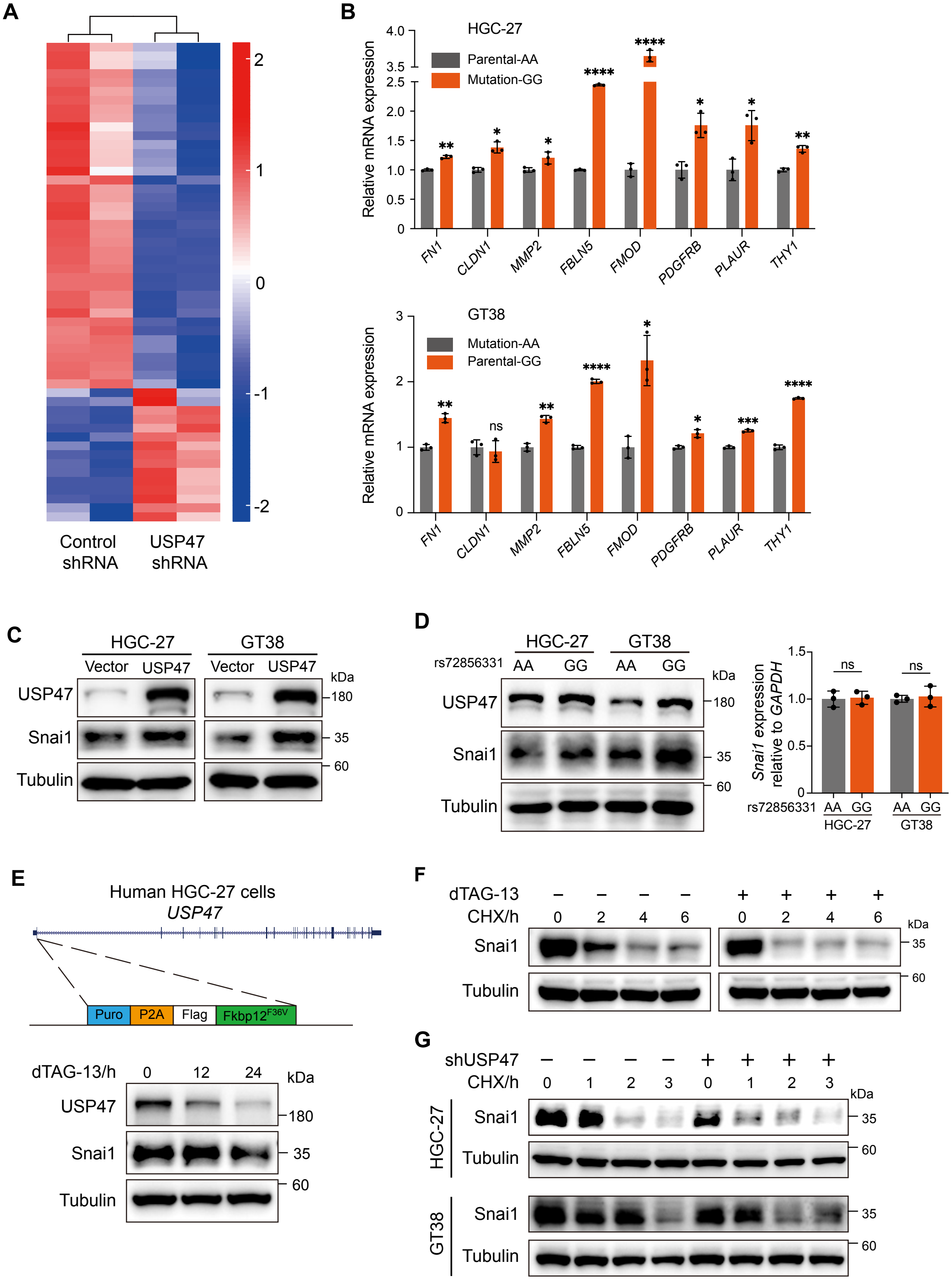

Supplemental Figure 12. USP47 regulation of Snai1 stability and its impact on EMT pathways and gene expression in gastric cancer

A. Heatmap visualization of RNA sequencing results from HGC-27 cells transduced with control shRNA compared to cells transduced with shRNAs targeting *USP47*.

B. RT-qPCR analysis of EMT-related genes in HGC-27 and GT38 cells with different genotypes of rs72856331 (n=3).

C. Western blot analysis of USP47 and Snai1 protein levels in HGC-27 and GT38 cells with USP47 overexpression.

D. Western blot and RT-qPCR analysis of USP47 and Snai1 expression in HGC-27 and GT38 cells with different rs72856331 genotypes at the rs72856331 locus (n=3).

E. Schematic representation of the dTAG system targeting USP47. Treatment with dTAG-13 for 12 and 24 hours results in significant degradation of USP47, leading to a marked decrease in Snai1 protein levels.

F. Western blots showing Snai1 protein levels in HGC-27 cells treated with cycloheximide (CHX), with or without USP47 knockdown.

G. Western blot analysis showing the effect of *USP47* knockdown on Snai1 protein stability in HGC-27 and GT38 cell lines following treatment with cycloheximide (CHX).

Data are presented as mean ± SD. Statistical significance was calculated using a two-tailed Student's t-test. *$P < 0.05$, **$P<0.01$, ***$P < 0.001$, ****$P < 0.0001$.

Tao.et al.Figure S13

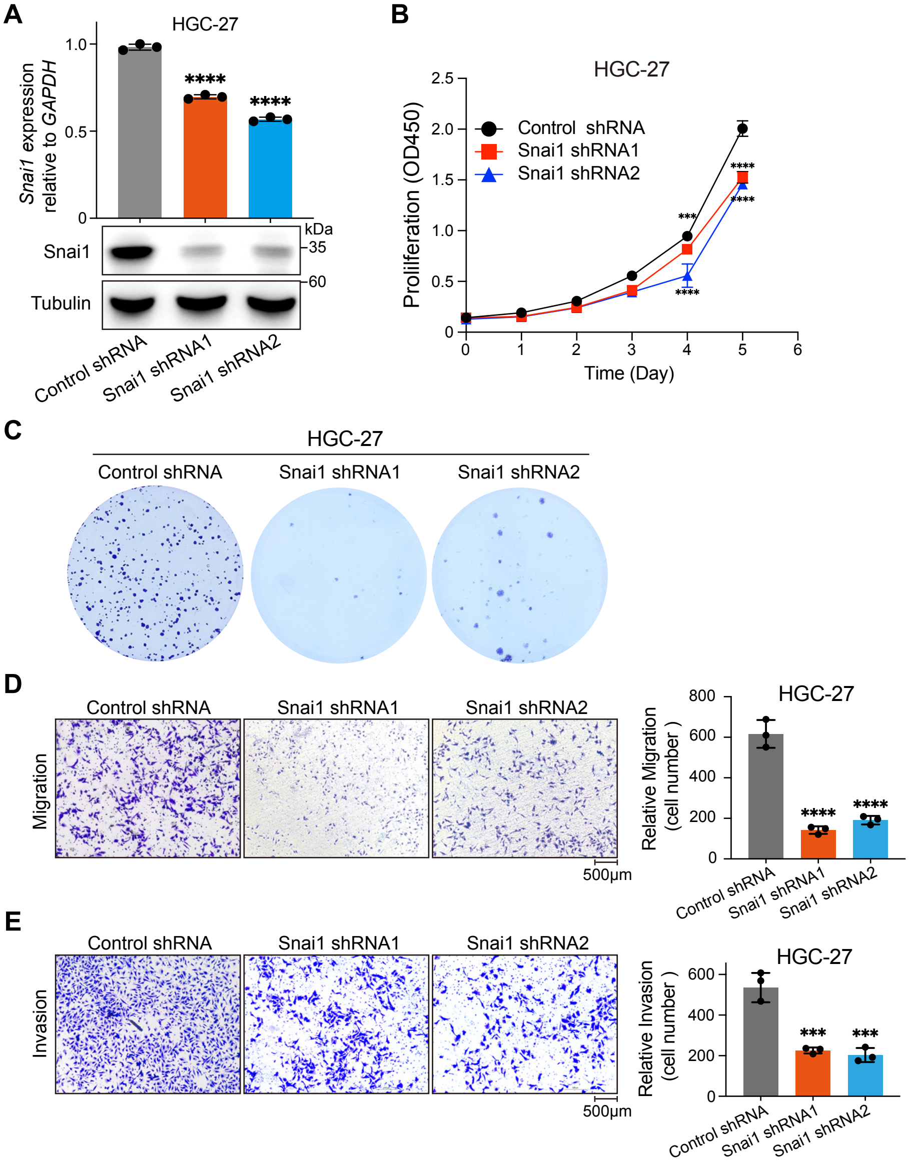

Supplemental Figure 13. Downregulation of Snai1 reduces malignant phenotypes in HGC-27 gastric cancer cells.

A. Quantitative PCR and Western blot analyses showing Snai1 mRNA and protein levels in HGC-27 cells after knockdown with two distinct Snai1-specific shRNAs.

B. Knockdown of Snai1 suppresses gastric cancer cell growth, as measured by the CCK-8 assay in HGC-27 cells transduced with control shRNA or Snai1-targeted shRNAs (n=3).

C-**E**. Representative images of colony formation (**C**), migration (**D**), and invasion (**E**) assays in HGC-27 cells transduced with control or Snai1-targeted shRNAs (n=3). Scale bars = 500 μm.

Data are presented as mean ± SD. Statistical significance was calculated using 2-way ANOVA and Holm-Šidák post hoc test for panel **B** or 1-way ANOVA with Holm-Šidák multiple comparison test for panels **D** and **E.** ***$P < 0.001$, ****$P < 0.0001$.

Tao.et al.Figure S14

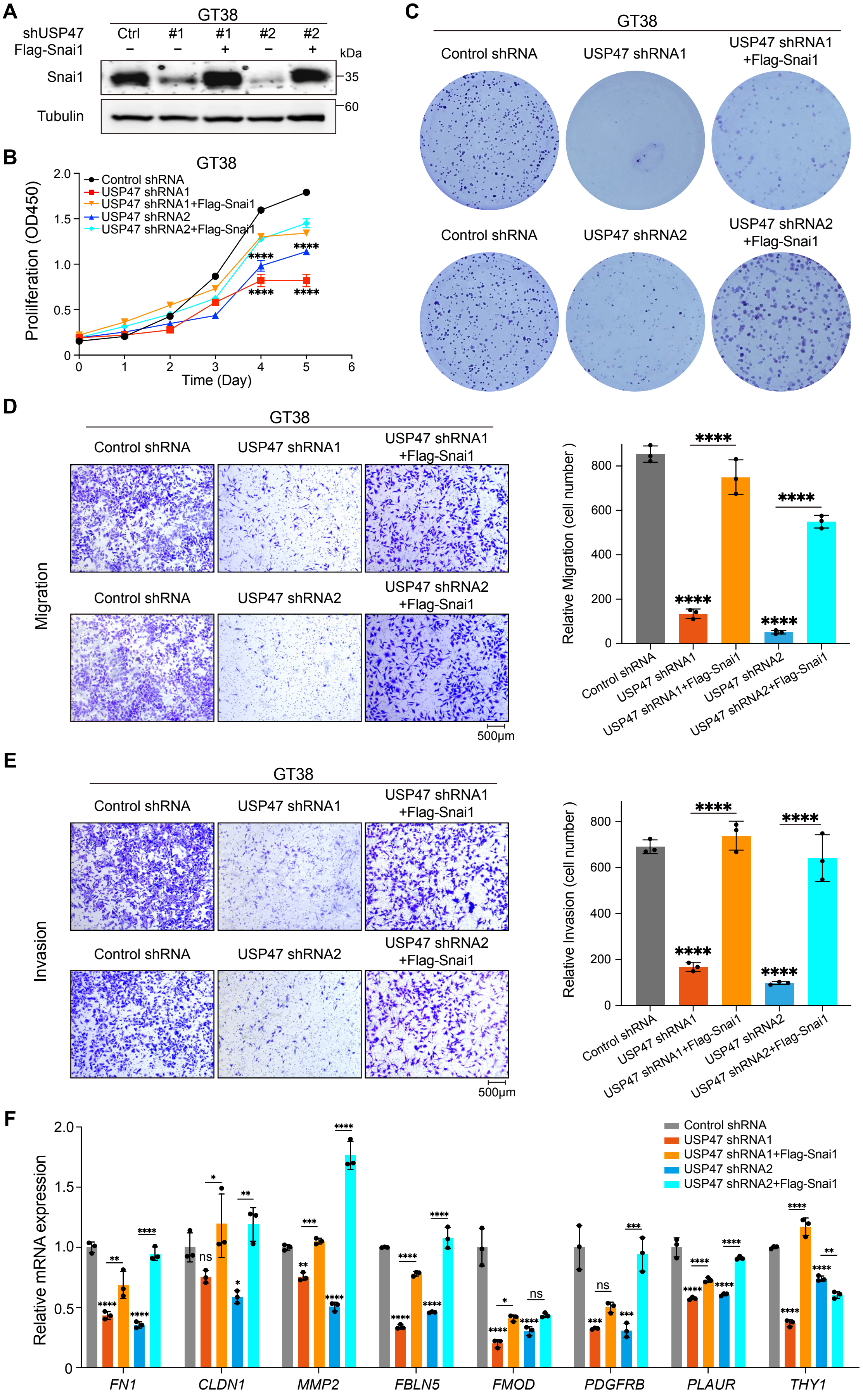

Supplemental Figure 14. USP47 promotes gastric cancer cell proliferation, migration, and invasion by regulating Snai1.

A. Western blot analysis confirming the knockdown efficiency of *USP47* using two independent shRNAs and the overexpression of Snai1 following co-transfection with Flag-Snai1.

B-**E**. Functional assays evaluating the effect of Snai1 overexpression on cancer cell phenotypes after USP47 depletion in GT38 cells: cell proliferation (**B**), colony formation (**C**), migration (**D**), and invasion (**E**) (n=3). Representative images are shown for the colony formation, migration, and invasion assays. Scale bars = 500 μm.

(**F**). Relative mRNA expression of EMT-related genes in *USP47*-knockdown gastric cancer cells with or without Snai1 overexpression (n=3).

Data are presented as mean ± SD. Statistical significance was calculated using 2-way ANOVA and Holm-Šidák post hoc test for panel **B** or 1-way ANOVA with Holm-Šidák multiple comparison test for panels **D, E** and **F.** $^*P < 0.05$, $^{**}P<0.01$, $^{***}P < 0.001$, $^{****}P < 0.0001$.
